# Robust extraction of functional signals from gene set analysis using a generalized threshold free scoring function

**DOI:** 10.1186/1471-2105-10-307

**Published:** 2009-09-23

**Authors:** Petri Törönen, Pauli J Ojala, Pekka Marttinen, Liisa Holm

**Affiliations:** 1 The Holm Group, Biocenter II, Institute of Biotechnology, PO Box 56, 00014 University of Helsinki, Finland; 2 Finnish Red Cross Blood Service Research and Development, Kivihaantie 7, 00310 Helsinki, Finland; 3 Department of Mathematics and Statistics, P.O. Box 68, 00014 University of Helsinki, Finland; 4 Department of Biological and Environmental Sciences, P.O. Box 56, 00014 University of Helsinki, Finland

## Abstract

**Background:**

A central task in contemporary biosciences is the identification of biological processes showing response in genome-wide differential gene expression experiments. Two types of analysis are common. Either, one generates an ordered list based on the differential expression values of the probed genes and examines the tail areas of the list for over-representation of various functional classes. Alternatively, one monitors the average differential expression level of genes belonging to a given functional class. So far these two types of method have not been combined.

**Results:**

We introduce a scoring function, Gene Set Z-score (GSZ), for the analysis of functional class over-representation that combines two previous analysis methods. GSZ encompasses popular functions such as correlation, hypergeometric test, Max-Mean and Random Sets as limiting cases. GSZ is stable against changes in class size as well as across different positions of the analysed gene list in tests with randomized data. GSZ shows the best overall performance in a detailed comparison to popular functions using artificial data. Likewise, GSZ stands out in a cross-validation of methods using split real data. A comparison of empirical p-values further shows a strong difference in favour of GSZ, which clearly reports better p-values for top classes than the other methods. Furthermore, GSZ detects relevant biological themes that are missed by the other methods. These observations also hold when comparing GSZ with popular program packages.

**Conclusion:**

GSZ and improved versions of earlier methods are a useful contribution to the analysis of differential gene expression. The methods and supplementary material are available from the website http://ekhidna.biocenter.helsinki.fi/users/petri/public/GSZ/GSZscore.html.

## Background

The analysis of differential gene expression between two sample types, such as pathological and healthy tissues, is one of the cornerstones of the modern biomedical science. Here typically the up or down-regulation of each gene in the pathological samples is measured. The obtained expression data can be considered as *Ordered Gene List, OGL*, by sorting it according to gene regulation. The upper end of the OGL represents the strongest up-regulation and the lower end the strongest down-regulation in the pathological sample. The middle area of the list represents genes with insignificant regulation. Similar gene lists can also be generated with various other data sources, like sequence similarity searches, high throughput screening of gene knock-outs, or protein expression arrays.

Originally, the analysis of differential expression focused on the few genes with the most extreme up or down regulation between the samples. This is sensitive to potential measurement errors, and prone to produce false positive findings, such as genes reacting to any sample handling. Furthermore, it cannot detect biological processes showing consistent but weak regulation between the two datasets. A central improvement was to carry out the analysis using pre-defined gene classes or gene sets, such as functional classifications [1-3]. The pre-defined gene classes usually show more stable behaviour as a group than at the single gene level, and classes also simplify the biological analysis [4,5]. Functional classes are typically used to study a subset of the genes, like the ones whose expression exceeds a pre-defined threshold. This subset is usually analyzed for over-represented functional classes, using Fisher's exact test. Currently several web tools, such as DAVID [6], implement over-representation analysis.

Although the utilization of the gene annotation classes moves the focus away from the actual single gene level to the more robust biological process level, it is strongly dependent on the definition of the used threshold. This has motivated many authors to propose threshold free gene set analysis methods (also called as Gene Set Enrichment Analysis [5] and Threshold free ontological analysis [7]). These methods monitor differential gene expression at the class level. They can be roughly divided into two categories: *Signal summary methods *take the whole gene list into account and calculate a mean or median based score for all the member genes of the class [8,9]. *Ranked list based methods *[5,10,11] analyze over-representation of the gene class by going through the whole OGL and test every possible position for the threshold. Usually the threshold with the strongest score is selected. The Kolmogorov-Smirnov score (KS) [5] and the hypergeometric p-value [11,12] represent two popular test scores for this analysis. The latter, hence called here iGA (iterative Group Analysis, as in [11]) represents a simple extension of the standard threshold based analysis. We refer to these functions, used to monitor differential gene expression at class level, as *class level scoring functions*.

Both types of methods have different drawbacks. Signal summary scoring functions assume that the whole gene set will show homogeneous behaviour. This is violated, for instance, when the gene set constitutes a pathway with genes repressing and inducing the amount of end-result, or when the gene set includes a significant amount of genes misclassified as members of the functional class. On the other hand, the ranked list based methods omit the score associated with each gene, and consider the step between consecutive genes always constant. Yet, in the real datasets one can see very drastic regulation at the tails of the gene list with clear differences between genes and small differences in the middle region with more or less random gene ordering. Indeed, one of the ranked list statistics has been criticized for being too sensitive to the signals in the central area of the gene list [13].

Here, we propose an improvement that avoids these problems by merging the two method groups. The main principle is to apply a statistic that is based on under or over-representation and to analyze the whole list for the strongest signal. The difference to standard ranked list methods is that the actual scores for the differential expression, assigned to each gene, are taken into account. A similar idea has already been presented [13], but the unique feature here is the derivation of the mean and the standard deviation of the score, allowing direct normalization as a Z-score (Gene Set Z-score, GSZ-score). As a result, the regularized version of the GSZ-score shows very stable behaviour, especially when compared to the similar earlier method [13]. GSZ-score can be linked with two standard test scores, the hypergeometric test and correlation between the gene set labels and the expression scores, and with two recent scoring functions, Random Sets by Newton et al. [14] and max-mean used by Gene Set Analysis [15]. Thus GSZ-score represents a novel unification between these different relevant scoring functions. It allows monitoring over and under-representation of the gene class within the subset of the gene list and also consistent shift of the gene class as a whole. The regularized version of the obtained GSZ-score is stable across different threshold positions in the gene list and across the different GO class sizes. To our knowledge, this is the first publication to analyze these stabilities. Furthermore, we present extreme value distribution (EVD) as an asymptotic distribution for GSZ-score p-value calculation. The presented method and test datasets are available from our web site http://ekhidna.biocenter.helsinki.fi/users/petri/public/GSZ/GSZscore.html.

We comprehensively evaluate the performance of GSZ-score and various other frequently used class level scoring functions. We focus our testing on class level scoring functions, by keeping everything else in the analysis pipeline identical. This ensures that the observed differences come from the evaluated functions. The same cannot be guaranteed when comparing software packages. Similar modular analysis of gene set analysis methods was recently recommended elsewhere [16]. We first test class scoring functions with artificial datasets. Differently from earlier similar work [7,10], we tested scoring functions with classes showing up-regulation and also with classes showing simultaneous up- and down-regulation. GSZ-score shows the best overall performance in these tests. In addition, we show optimal regions from the artificial signals for different scoring functions. Here, GSZ-score has a good performance with signal types that were considered important in earlier publications [5,7].

Next, we do repetitive testing of class scoring functions on real data set. As the correct positive and negative classes are not known in real data, we used the evaluated functions to predict them. We used *Cross validation *style evaluation, where data was split to halves, with the first half used to predict the positive and negative classes and the other half used to test the competing methods. Test situation was replicated by (*i*) using each function itself to select the positive classes from the other half and (*ii*) by combining the results of all the methods from the other half and using this same set for all methods. Both tests were repeated with different number of positive classes. In addition, we further confirm our results by monitoring the correlation between the test and control results. GSZ constantly shows the best performance over different test situations, whereas the performance of other functions varies between the test situations.

We further evaluate the analyzed class scoring functions by generating large set of randomizations. Two very different datasets were used here. We monitored the magnitude of different empirical p-values, in order to see the risk of potential false positive findings for each function. Results showed the best performance for GSZ with small difference on one dataset and with very clear difference on the other dataset. We point out that all the observed differences in numerical data evaluations resulted from the class scoring functions, establishing GSZ-score as outstanding in this group. In addition, real data analysis shows weak performance by some of the popular scoring functions. This is, to our knowledge, one of the most detailed numerical evaluations of class scoring functions on real datasets.

We also carried out a detailed biological evaluation of scoring functions. This was done by ordering GO classes with empirical p-values and monitoring top GO classes. Next, we defined biologically relevant classes for both datasets and analyzed how different methods were able to find them. GSZ-score clearly shows outstanding performance in finding the important biological themes on both datasets. Again some of the competing scoring functions evidently show weak performance on one or both of the datasets. Results were further analyzed by monitoring GO classes with largest pairwise difference between the GSZ-score and competing scoring function. Classes where GSZ-score outperformed others showed stronger differences in p-values and were clearly linked to the research setup.

We also performed a comparison with some of the most widely used software packages in this research topic. This analysis is more difficult to evaluate due to the other potential sources of differences between software packages. However, this ensures that we evaluate the state of the art implementations of class scoring functions. We concentrate here on the evaluation of the reported list of GO classes, and mostly omit the reported p-values. GSZ again showed best performance on two tested datasets, when evaluated with biologically relevant classes.

## Results

A typical pipeline for gene set analysis starts by looking at the differences in the gene's behaviour in the two datasets, like in treatment and control. This can be done, for example, by calculating a score based on the difference between the mean expression values, like t-test score, with standard t-test or its variations [17,18]. We call these primary tasks *differential expression tests *and their scores as *differential expression test scores *for clarity. The obtained gene list is then sorted using the selected score. In the next step, gene classes, one at the time, are taken to the analysis and the gene list is analyzed using either rank based methods or signal summary methods. We call these tests *class scoring functions *and their results *class expression scores*. Our aim is to improve the existing class scoring functions. For more information on this topic see earlier publications [5,10,11,13].

### Novel Gene Set Z-score

We start with a ranked list based analysis, where a threshold is placed repetitively in between every consecutive pair of genes in the ordered list. The obtained subset is then used to calculate a selected statistical score. This test score can be a difference between two empirical cumulative distributions like in Kolmogorov-Smirnov statistics [5] or a log(p-value) calculated from Hypergeometric distribution (iGA, [11]) among others.

We propose to improve these tests by taking the differential expression scores into account. Let *M *denote the total number of genes, and let *X*_*i*_, *i *= 1,..., *M*, denote the differential expression test score for the *i*th gene. Furthermore, let *S*_*N *_be a subset of *N *genes from the upper end of the OGL (i.e. *N *genes with the highest differential expression test score values *X*_*i*_). We use a simple function:(1)

This denotes a difference between the sums of scores for members (positive gene group, *pos*) and non-members of a gene class (negative gene group, *neg*), among the *N *genes with the highest differential expression test scores. This will then be calculated separately with each threshold position *N*, and analogously for the lower part of the OGL. To simplify the notation, the subset *S*_*N *_will be kept implicit in our notation by dropping the subscript *N *in the sequel. A similar but more complex idea has been proposed in the article that motivated this research [13] (see also Methods in supplementary text S1 [additional file 1]). However, we decided to use the simpler equation as:

• Z-score normalization is easier to perform for the plain difference.

• The obtained Gene Set Z-score (GSZ-score) includes other popular methods as limiting cases (discussed below).

• The resulting score is only affected by differential expression test score values within the selected subset, whereas in the equation in [13], the whole distribution of class member gene scores affects the weighting of the class members. Our idea should therefore, in principle, be more efficient in observing simultaneous up and down-regulation.

• As a more theoretical benefit, the obtained GSZ-score treats both class members and class non-members similarly. So the results stay identical if we flip the class member and class non-member classifications.

The selection of the starting equation 1 is still somewhat an open issue and our main emphasis is rather on the Z-score normalization for gene set presented later. The similarities with other class scoring functions are discussed more in detail in the supplementary text S2 [see additional file 2], where we show that GSZ-score links together Random Sets scoring function [14], max-mean for Gene Set Analysis [15], hypergeometric Z-score and correlation between the class labels and the gene expression scores.

The raw difference is an unusable statistic due to the biases caused by different sizes of classes, different sizes of subsets, and different variances of the scores in different subsets. However, these can be corrected by calculating estimates for the expected value (mean, *E*(*Diff*)) and variance (*D*^2^(*Diff*)) for the eq. 1 under the null hypothesis, that the class members and non-members are distributed randomly across the list. This allows us to use a Z-score normalization with equation:(2)

where *E*(*Diff*) = 2*E*(*X*)*E*(*N*) - *ME*(*X*) and(3)

where *N *is the number of positive genes in the subset, *M *is the size of the subset, *E*(*N*) is the mean and *D*^2^(*N*) is the variance of the hypergeometric distribution of the number of positive genes *N *for the analyzed subset. *D*^2^(*X*) and *E*(*X*) are the variance and the mean of the differential expression test scores for the subset. The derivation of *E*(*Diff*) and *D*^2^(*Diff*) is shown in Methods. As a further improvement to our GSZ-score we consider a regularized version:(4)

This is otherwise similar to eq. (2) but we add a *prior variance k *to the variance estimate inside the square root. This was done due to the too unstable behaviour with small subsets (see supplementary text S1, [additional file 1]). The regularization represented here should not be mixed to normalization that is performed later with the randomizations. Note that prior variance is regularly used with t-test in the gene expression data analysis [17,18]. Supplementary text S1 [additional file 1] shows that the regularized version has a more stable behaviour. We have used two rules for defining the prior variance *k*: We take the median of the variance estimates, obtained with the analyzed class across the whole gene list, and multiply it with a weight, w2 (0 ≤ *w*2 ≤ 1). We also take the median of the variance estimates, obtained with the class of size 10 across the whole gene list, and multiply it with a weight, w1 (0 ≤ *w*1 ≤ 1). Next the weighted variance medians are summed to obtain a value for *k*. Class size of 10 is used here as a reference class size, penalizing especially classes smaller than 10. This class size selection is 'ad hoc', but it has performed well in our analysis. We tested 12 different combinations for these two weights, with artificial data, and selected the best performing parameter values for real dataset analysis. The calculated GSZ-scores form a profile of score values over the OGL obtained from each cut-off position, including also the values corresponding to the whole gene list as a subset. From the obtained scores we select the maximum value similarly to earlier works [5,11,13]. Other options are stated in discussion.

### Stability of various class scoring functions with row and column randomizations

First we tested the stability of various class scoring functions with randomized real datasets. The tested scoring functions in the results are: GSZ-score, standard and modified Kolmogorov-Smirnov test (KS, modKS [5,13]), threshold free implementation of hypergeometric test (iGA, [11]) and t-test (with added prior variance) calculated between the expression values of class members and class non-members. T-test reflects here the behaviour of signal summary methods [8,9]. We use two types of randomizations: in *row randomization *the gene classifications associated with dataset rows are randomized, and in *column classifications *the sample labels associated with data columns are randomized. The aim was to see what type of biases the methods show as the GO class size varies, or as the threshold moves through the gene list. These results are shown in the supplementary text S1 [see additional file 1]. Here, we highlight a few details: The use of the prior variance *k *significantly stabilizes the GSZ-score, with a group of tested parameter values for the *k *obtaining equally good results (see supplementary figure S1, [additional file 3]). Regularized GSZ-score has a stable signal distribution over all the threshold positions with a randomized dataset, whereas two other evaluated methods, KS and modKS show significantly less stable behaviour over different threshold positions (see supplementary figure S2, [additional file 4]). Furthermore, a larger randomization shows very good stability for GSZ-score in row randomizations as the size of the class is varied. A more detailed analysis is in supplementary text S1 [see additional file 1].

In addition, we show in supplementary text S1 [see additional file 1] the dramatic biases caused by gene level correlations, discussed also earlier [13,15,19]. These can be seen when analyzing the column randomizations. We show the bias only for the most stable methods from earlier row randomizations (see supplementary text S1, [see additional file 1]), but it can be expected to affect all the methods. Here, mean and STD estimates from the column randomizations were tested for normalization of the results. The stability of the estimates was evaluated by dividing the randomized dataset into testing and learning data, where learning data was used to obtain the estimates and the testing data was normalized using the estimates and visualized to see the stability of the normalization with randomized data. The obtained normalization looks adequate with 100 randomizations. Despite the adequate normalization, the later steps use larger numbers of randomizations, as they will enable better analysis of empirical p-values for the observed results.

### Evaluation of class scoring functions with artificial data

Next, we evaluate the class level scoring functions on very large set of various artificial datasets. We currently lack complete knowledge on what can be considered as a positive signal for a differentially expressed gene class. However, we propose a few signal types that can be intuitively justified. **Case 1**: The whole gene class shows a similar type of regulation, either up or down. Nevertheless, the complication in this simple case is that the signal for the gene class can be quite diffuse with larger STD, depending on the different measurement efficiencies for various RNAs. **Case 2**: only a subset of the gene class shows signal (up- or down-regulation) like in case 1. Potential explanations are: Only a subset of the gene class requires regulation or the gene class includes false positive genes that are not really members of the gene class. **Case 3**: Gene class shows both up and down-regulation at the same time. Here gene class can comprise differently behaving subclasses or the members can be from a pathway with activities that either increase or decrease the activity of the pathway.

Here we have used an artificial data generation similar to previous work [7,10]. Test covers an even larger number of various signal subtypes for positive classes than previous research. We vary the size of the gene class that represents the signal, the magnitude of positive signal, the standard deviation of the positive signal, and the percentage of gene class that shows positive signal, as in the previous work. The signal is also either set to be only in positive direction (only up-regulation, case 1 and case 2), or the genes representing the signal are set to be randomly either positively or negatively regulated with equal probability (case 3). To our knowledge, this is the first work evaluating both of these cases to this extent. Details on the various parameters used in the artificial signal generation are shown in the Methods (supplementary text S1 [see additional file 1]).

First, the analysis was carried out with various gene class sizes, testing each class size separately. Thus, the compared results for positive and negative cases are obtained with the same gene class size. Next, the results for different class sizes and signal types are combined. This measures the performance of the method in the case where the various gene class sizes are separately normalized with mean and standard deviation estimates obtained from randomized data. This is currently the standard procedure of most programs. In another option, the positive and negative results for different class sizes are pooled and the separation is monitored within the pooled data. This measures the stability of the functions when the gene class size varies. This corresponds to the performance of the method without any class specific normalization with randomized data (proposed for example with iGA [11]).

In each testing situation with each parameter setting for positive datasets we generate 200 positive datasets and 600 negative datasets. Next, we calculate the test scores with each of these datasets. We used Area Under receiver-operating characteristics Curve (AUC) as a measure of separation. This was considered better than the analysis of power [7], as power analysis requires a selection of the p-value cut-off, measuring separation only at that point, whereas AUC measures the separation across the whole list. We have first summarized the information from four testing situations by comparing every method pair and calculating two scores:(5)

where *i *and *j *specify methods that are compared and *l *= 1, 2.. *R *refers to dataset, obtained with specific parameter values. Score A represents the mean of differences of AUC scores for two methods across all the datasets in the testing situation, whereas score B represents which of the methods, *i *or *j*, was more frequently better. We generated with both of these scores a difference matrix representing all the method pairs. Next the average for each method *i *was taken, by letting *j *go over all the methods. Now we have two measures for the tested method: One corresponds to average of difference to other methods and the other corresponds to frequency of better performance than with other methods. Results for these two scores are shown in table 1.

**Table 1:** Summary of method performances from artificial data analysis

	parameters (if any)		fixed size, up-regulation		fixed size, up and down-regulation		varying size, up-regulation		Varying size, up and down-regulation		included to fig. 1 and 2
	**w1**	**w2**	**Score A**	**Score B**	**Score A**	**Score B**	**Score A**	**Score B**	**Score A**	**Score B**	

GSZ-score	0	0.1	-0.0002	*-0.1898*	0.0867	0.184	0.0111	0.0837	**0.1025**	**0.4976**	
GSZ-score	0.1	0.1	0.003	-0.0896	0.0883	0.26	0.0095	0.0988	0.1003	0.4669	
GSZ-score	0.2	0.1	0.0048	0.0119	0.0891	0.3061	0.0065	-0.0085	0.0965	0.2528	
GSZ-score	0.5	0.1	0.0074	0.1567	0.0898	0.3006	*-0.0031*	*-0.355*	0.0837	-0.0515	
GSZ-score	0	0.3	0.0054	0.0485	**0.0901**	**0.33**	**0.018**	**0.5143**	**0.1073**	**0.7216**	
GSZ-score	0.1	0.3	0.0067	0.1327	**0.0904**	**0.3466**	**0.0156**	**0.4358**	**0.1043**	**0.5892**	
GSZ-score	0.2	0.3	**0.0076**	**0.1892**	**0.0904**	**0.3427**	0.0128	0.2623	0.1007	0.3901	
GSZ-score	0.5	0.3	**0.0088**	**0.2547**	0.09	0.2874	0.0041	-0.1618	0.0895	0.0725	
GSZ-score	0	0.5	0.0054	0.0485	**0.0901**	**0.33**	**0.018**	**0.5143**	**0.1073**	**0.7216**	X
GSZ-score	0.1	0.5	**0.0081**	**0.2245**	0.09	**0.309**	**0.018**	**0.5787**	**0.1049**	**0.5659**	X
GSZ-score	0.2	0.5	**0.0086**	**0.2609**	0.0898	0.2778	**0.0154**	**0.3876**	0.1016	0.3828	X
GSZ-score	0.5	0.5	**0.0094**	**0.2919**	0.0892	0.2115	0.0077	-0.019	0.0917	0.1538	X
t-test	0		*-0.0165*	*-0.2968*	*-0.2707*	*-0.7744*	*0.0056*	*-0.1683*	*-0.2454*	*-0.8415*	
t-test	0.1		*-0.0143*	*-0.2494*	*-0.2646*	*-0.7142*	0.0074	-0.0679	*-0.2385*	*-0.7783*	
t-test	0.3		*-0.0121*	*-0.2058*	*-0.2546*	*-0.6561*	0.0087	0.005	*-0.2275*	*-0.7206*	
t-test	1		*-0.0094*	*-0.1588*	*-0.2324*	*-0.6022*	0.01	0.0675	*-0.204*	*-0.6582*	X
t-test	3		-0.0079	-0.1024	*-0.2065*	*-0.5425*	0.0106	0.1249	*-0.1775*	*-0.5926*	X
KS			-0.0001	-0.0268	0.0462	0.0027	*-0.062*	*-0.5063*	-0.0439	-0.3352	X
modKS			*-0.0164*	-0.1558	-0.0231	-0.3216	*-0.0449*	*-0.5979*	-0.076	-0.4214	X
iGA			0.0075	0.0911	**0.0951**	0.2485	*-0.0058*	*-0.453*	0.0827	-0.0325	X

Table shows average performance of various methods and parameters with artificial datasets. Scores A and B are explained in the main text. The five best methods are highlighted with bold and underlined in each column. The next five methods are underlined. Five weakest scores are represented in italics. Scores obtained with fixed class size represent the performance when the results are normalized with class specific permutation, whereas the results with varying class size show the performance without any normalization.

**Figure 1:**
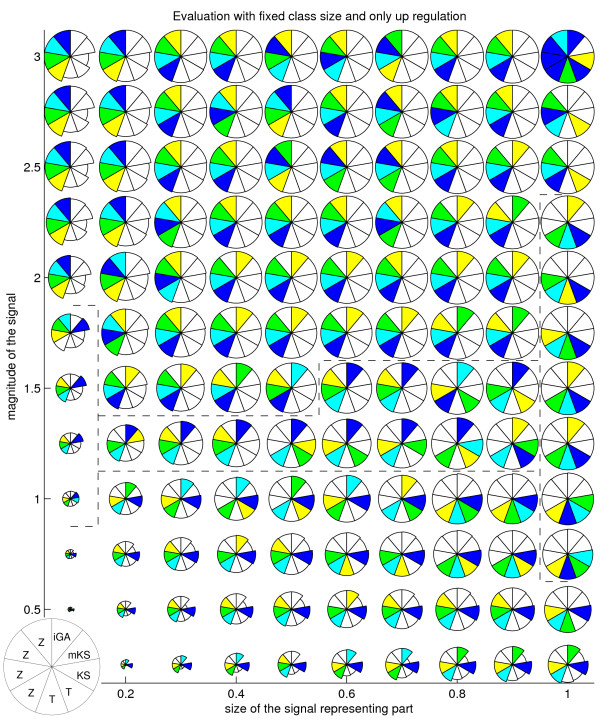
**Method performances for different artificial signals, when only up-regulation occurs**. Method performances for each proportion of the signal representing part (Y axis) and signal magnitude (X axis) as measured by average AUC score. Score is shown by the radius of each sector. Methods represented are (starting from 11 o'clock, anticlockwise): 4 versions of GSZ-score, 2 versions of t-test, KS test, modKS test and iGA. Selected methods/parameters are shown in detail in table 1. Colouring (blue, cyan, green, yellow) highlights the four best methods, with equally well performing methods represented with the same colour. Dotted lines separate areas where different methods show the best performance. GSZ-score, selected to later analysis, is at 9 o'clock position.

**Figure 2:**
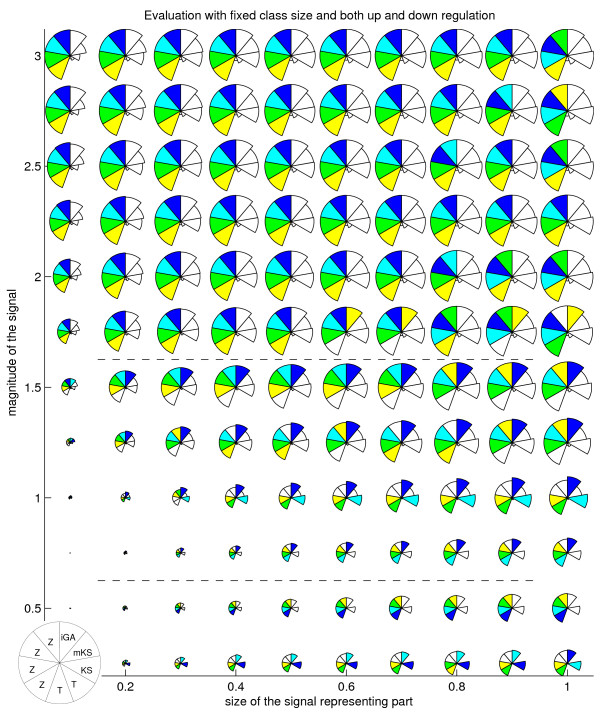
**Method performances for different artificial signals, when class shows simultaneous up and down-regulation**. Method performances for each proportion of signal representing part (Y axis) and signal magnitude (X axis) as measured by average AUC score. Score is also here shown by radius. Methods represented are the same as in the previous figure. Colouring (blue, cyan, green, yellow) again highlights the top methods. Dotted lines highlight the signal levels where the best method changes.

The results show the best overall performance for GSZ-scores. The only exception to the rule is the better performance of iGA in the testing with simultaneous up and down-regulation with fixed class size. Even there GSZ-scores showed good performance in a larger subset of the datasets (shown by the score B). This discrepancy of two measures proposes that we frequently see slightly better performance from GSZ-score and a small subset where iGA clearly surpasses GSZ-scores. Subsequent analysis links this to datasets showing signal around 1 STD (discussed later with fig. 1 and fig. 2). However, with real datasets this testing situation would require normalization of iGA results with several randomized runs, which is time consuming. Indeed, when the evaluation is done without class size normalization, iGA performance drops. Table 1 also suggests potentially best regularization parameter values for GSZ-score. None of the parameter values is optimal in all cases. Yet w1= 0.1 or 0.2 and w2 = 0.3 or 0.5 show good performance across the tests. From the obtained results we selected w1 = 0.2 and w2 = 0.5 for later analysis, although we point out that other nearby parameter values yielded equally good results. t-test has been similarly evaluated with several parameter values (with w1 = 0 as the standard t-test), with the best performance always occuring with the largest value w1 = 3.

Table 1 summarizes a large amount of information, when in reality methods perform differently with different types of signal [7]. This was highlighted by visualizing the score for each combination of signal representing subset size and signal magnitude separately, like in previous work [7]. Each result is averaged over tested class sizes and standard deviations of the signal. We further modify the earlier visualization by marking the best ranking methods with colours (blue, cyan, green, yellow for the best, the 2nd, the 3rd and the 4th best). The following work concentrates on analysis done with each class size separately.

Fig. 1 and 2 reveal trends, pointing out that no test is optimal across all the signal types. GSZ-score, with different parameter settings, is among the top methods across the most signal types. If we look at fig. 1, KS method shows the best performance with small signal magnitudes (signal between 0 - 1 STD. STD refers here to the standard deviation of the background distribution.) This was followed by iGA, showing best performance when signal is between 1.25 - 1.5 STD and signal representing portion between 0.6 - 0.9. GSZ-score is optimal when signal is larger than 1.5 STD or the signal portion is smaller than 0.4. The only deviations from this trend are modKS, which is optimal when signal is between 1 - 1.75 STD and signal proportion is 0.1, and t-test, which is optimal when whole class shows signal that is between 0.75 STD and 2.25 STD. A simpler view is obtained by looking at results obtained with simultaneous up and down-regulation (fig. 2). Here, good performance is only represented by KS test (when signal ranges 0.25 - 0.5 STD), iGA (when signal ranges from 0.75 - 1.5 STD) and GSZ-score (when signal is larger than 1.5 STD). Similar results were obtained for cases where class sizes are allowed to vary. The only exception is that in these tests GSZ-score and t-test clearly improve, and the performances of iGA and especially KS drop (data not shown).

### Evaluation of the methods with real dataset

Previous analysis evaluated various methods using artificial datasets. The problem with such simulated data was that we do not have knowledge on how regularly one sees various signal types. Furthermore, the artificial data analysis assumed totally independent genes, which is a biologically unrealistic assumption. Therefore it is crucial to evaluate the performance also with real datasets. We use here ALL (Acute Lymphatic Leukaemia) dataset [20], due the reasons discussed in the Methods. These analysis steps are also represented as pseudo codes in supplementary text S1 [see additional file 1].

With a real dataset the true positive and true negative GO classes (also referred to as *gold standard*) are not known. Therefore we used the methods themselves to estimate the positive classes. This can omit some positive classes that none of the methods can discover and equally report some negative classes as positive. Nevertheless, we are not aware of any other realistic solutions for selecting positive GO classes, and minor errors should not corrupt our results. We also presume that the GO classes form a smooth transition with strongly positive GO classes at one end and strongly negative GO classes at the other end rather than a sharp binary classification to positive and negative classes. We therefore decided to use a *varying rank threshold*, selecting *q *best classes (*q *= 1, 2...200) from each method as positive (similar to [15]). This allows each method to report exactly the same number of positive GO classes and enables testing with a varying number of positive GO classes, concentrating to the best ranks in the selected positive GO class list. The competing idea, with selection of a fixed set of positive GO classes, would depend on the used p-value threshold and on the used multiple testing correction. Furthermore, it would place methods depending on their stringency in unequal situation, as methods with a tendency to report many classes as positive would contribute a larger portion of positive GO classes. Also, the evaluation of sorted GO classes with decreasing significance resembles the popular analysis of GO classes as a sorted list.

We chose the AUC score to see how well the sorted list of GO classes separates different selected positive and negative classes. These results were also further confirmed with overall rank correlation between the ordered gold standard GO class list and ranked GO list from the evaluated method. Two methods have different aims: AUC with varying rank threshold focuses on most positive GO classes in control set, whereas the rank correlation monitors the overall correlation between the test and control set. Resulting evaluations are based on rank rather than the actual score values obtained for different methods. This was considered to be beneficial as different methods can be expected to follow different distributions, and including the actual score values would therefore put methods in unequal position. Only case where also the actual signal scores were used was the comparison of method with itself using pearson correlation (see table 2).

**Table 2:** Rank correlations for each method with a gold standard obtained from the other half of the dataset

	1st split		2nd split		3rd split		4th split	
**method**	**1st half**	**2nd half**	**1st half**	**2nd half**	**1st half**	**2nd half**	**1st half**	**2nd half**

GSZ-score	**0.6201**	**0.6219**	**0.6421**	**0.6451**	**0.6594**	**0.6368**	0.6176	**0.653**
t-test	0.5373	0.5276	0.5691	0.5748	0.5722	0.5827	**0.6190**	0.5727
KS test	0.4470	0.5089	0.4981	0.5140	0.5340	0.5054	0.4928	0.5388
modKS	0.5048	0.5772	0.5339	0.6035	0.5957	0.5336	0.5756	0.5873
iGA	0.5809	0.5888	0.6110	0.6133	0.6191	0.6148	0.5976	0.6185

Table shows rank correlation between the results for each half of a dataset with the gold standard ranking, obtained with all the methods from the other half (case *ii *evaluation). Various results are highlighted similarly to previous table. Two results for each split are obtained by testing the first half with a gold standard from the second half and testing the second half with a gold standard from the first half. Notice that GSZ-score clearly shows the best correlation. iGA is usually the second best method. The only exception is the first half of the 4th split, where t-test shows the best performance and GSZ-score scores as the close second best method.

One of our evaluation setups requires the combination of the gold standards from all the evaluated methods. It would be natural to combine the rank results from various methods by averaging, but instead the best rank for each class was used here. This was done to minimize the effects of correlations between the methods, as with mean score two identically behaving methods would contribute twice in the positive class selection, whereas with the maximum score they would contribute only once.

The evaluation was done in two ways: *i*) Method itself is used to define *k *best GO classes from one half of the dataset. Next the method is run using the other half of the dataset to see how well it can predict its outcome from the correlating dataset. *ii*) All methods are allowed to select *k *best GO classes as positive GO classes using one half of the dataset. Next, each method is run on the other half of the data and it is tested how well they can predict the combined positive outcome of all the methods from the correlating dataset. Results from both cases were analyzed using AUC with varying rank threshold and also with standard correlations.

These evaluations have different evaluation principles. In case (*i*), we use each method itself to define gold standard from the separate data, selecting the method that is most robust to the variances between the biologically replicated datasets. Furthermore, as each method is tested against its own results, the occurrence or lack of correlation with other scoring functions does not affect the evaluation. However, this could favour methods that have biased selection, like a preference of large classes. Neither does it penalize methods that miss GO classes that other methods are able to find. In case (*ii*), the combined results of all the methods from the separate data represents the same gold standard for all methods. Here method has to be robust to variances between the datasets, and also be able to predict combined positive classes coming from all the methods.

**Figure 3:**
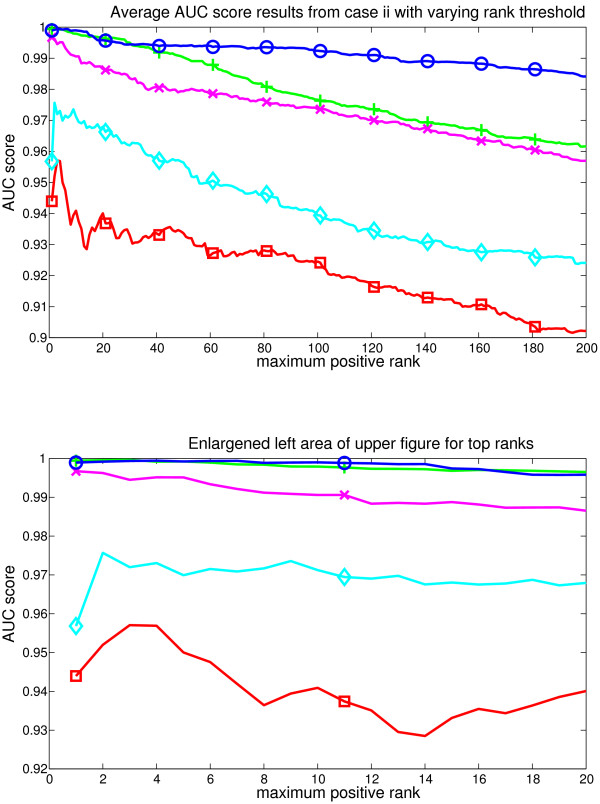
**Average results from the case i**. Figure represents the AUC score for each evaluated method as the rank limit of the positive GO classes is increased. The set of positive classes used for AUC grows as the rank threshold becomes bigger. Methods represented are GSZ-score: blue line with circles, t-test: green line with cross, KS test: red line with box, modKS test: cyan line with diamond, iGA: magenta line with x. Lower part zooms into the smallest ranks. Here GSZ-score shows the best performance and t-test performs equally well with the top ranks, while other methods show weaker performance.

**Figure 4:**
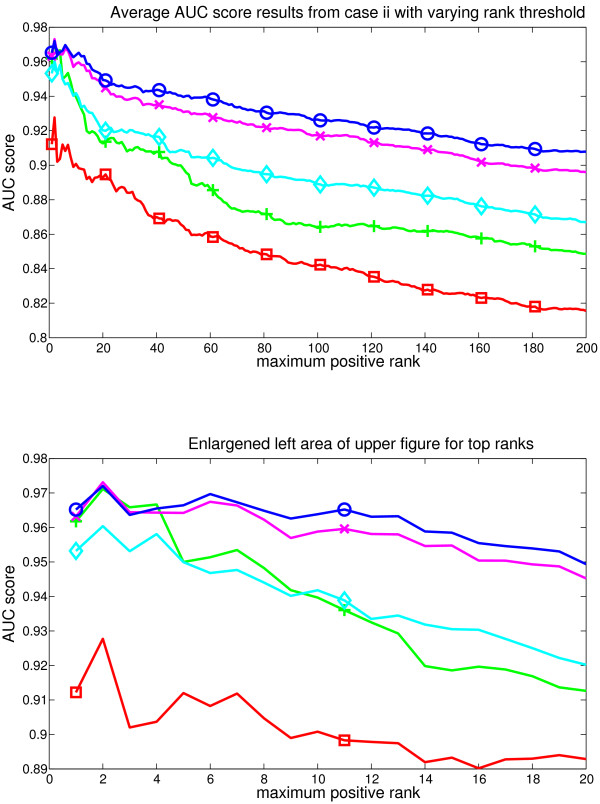
**Average results from the case ii**. Figure represents the AUC score for each evaluated method as the rank limit of the positive GO classes is increased. Methods are coloured identically to the earlier figure. Here, the GSZ-score shows the best performance and iGA is the second best method.

These analysis steps were done with four splits of the dataset (8 replicates). Results, averaged over all the replications, are shown in fig. 3 and 4 and tables 2 and 3. Also each split was analyzed separately [see additional files 5 and 6]. Results vary considerably at small ranks between replicates, especially in case (*ii*), but they stabilize at larger ranks, especially when rank threshold is between 10 and 20. This can be explained by the increasing size of the positive class set, used in the AUC scoring, which is potentially less sensitive to variations and errors in its definition.

**Table 3:** Rank and Pearson correlations for each method's results between the split parts of the dataset

	1st split		2nd split		3rd split		4th split	
**method**	**Rank**	**Pearson**	**rank**	**Pearson**	**rank**	**Pearson**	**rank**	**Pearson**

GSZ-score	**0.6878**	**0.8427**	**0.7021**	**0.8463**	**0.7051**	**0.8521**	0.6867	**0.8194**
t-test	0.6236	0.7291	0.686	0.7715	0.6932	0.7763	**0.7404**	0.7996
KS test	0.5402	0.585	0.5768	0.6197	0.5824	0.6282	0.5845	0.6145
modKS	0.6183	0.6397	0.6443	0.6658	0.6343	0.6554	0.6952	0.7083
iGA	0.6134	0.7261	0.6424	0.7401	0.6358	0.7562	0.6239	0.7243

Rank and Pearson correlation between the results from two halves of the dataset for each method (case *i*). The best result is highlighted with bold font and the second best is underlined. Notice that GSZ-score has the highest correlation with both correlation measures. t-test shows the second best performance. The only deviation is the 4th split where t-test shows the best rank correlation, although even in that case GSZ-score still shows the best Pearson correlation.

In case (*i*) GSZ-score shows best performance, with an even performance to t-test at the very first ranks. The detailed analysis of differences in replicates (see supplementary figure S3, [additional file 5]) showed equal performance between ranks 1-40 and the performance of t-test drops to clear separation after rank 40. The observed difference in results suggests that GSZ-score might see a larger set of classes with positive signal than t-test, and later analysis steps confirm this. Separation to other methods was obvious with iGA, modKS and KS showing consistently weaker AUC scores across all the ranks in all splits. These results are further confirmed with rank and Pearson correlations, which show the clear best performance for GSZ-score (table 3).

In case (*ii*) GSZ-score shows the best performance, and iGA is the close second best method. Their performances overlap in replicates across the top 10 ranks and after rank 20 GSZ-score consistently tops iGA in all replicates (see supplementary figure S4, [additional file 6]). T-test shows also somewhat equal performance at top 5 ranks but then it drops. ModKS shows also slightly better performance than GSZ-score in two of the replicates at very smallest ranks. These are only a few rank positions, and the performance of modKS drops at these replicates extremely fast. Indeed, modKS is mainly 3^*rd *^or 4^*th *^method, and it is the weakest method at the smallest rank in one of the splits. After rank 20 the order of methods is consistently GSZ-score, iGA, modKS or t-test and KS. Rank correlation analysis confirms also these results (table 2) showing the best performance for GSZ-score.

The overall performance of GSZ-score was best in these evaluations, as in case (*i*) it slightly surpassed t-test but is clearly better than iGA and in case (*ii*) it slightly surpassed iGA but was obviously better than t-test. Furthermore, it consistently topped modKS in case (*ii*) and clearly showed better overall performance in case (*i*). It was also observed that KS test shows the undoubtedly weakest performance in both tests. For a novel method, like GSZ-score, it is also interesting to see which methods are most similar to it. This was analyzed with the whole ALL dataset, using rank correlation. Results are shown in table 4. The correlations that GSZ-score shows with iGA and and t-test further underlines the GSZ-score's mathematical similarities with these methods.

**Table 4:** Rank correlations between results obtained by different methods

	GSZ	t-test	KS	modKS	iGA
GSZ	1	0.754	0.708	0.665	**0.887**
t-test	**0.754**	1	0.685	0.643	0.708
KS	0.708	0.685	1	0.502	**0.809**
modKS	**0.665**	0.643	0.502	1	0.627
iGA	**0.887**	0.708	0.809	0.627	1

Rank correlations between all the method pairs obtained using the whole ALL dataset and normalized results. Strongest correlation (excluding the diagonal) is highlighted on each row with a bold font. Notice the strong correlation between t-test and GSZ-score and the very strong correlation between iGA and GSZ-score.

### Comparison of empirical p-value signals

Earlier evaluations omitted the number of significant classes reported by each scoring function. This is, however, a relevant feature as smaller p-values and larger number of significant classes point out that the method is better in extracting signal. This evaluation was first done with ALL dataset [20]. Empirical p-values were obtained by using 1000 row and column randomizations and selecting always the larger, less significant, of two p-values. Only the scoring function is changed in this comparison and everything else is kept identical. We exclude the usage of asymptotic distributions as they are not available for all functions. All the results are shown here as -log10(p-values). We compared top 100 GO classes from each function by plotting the empirical p-values for these classes. Cases where log cannot be calculated (empirical p-value = 0) were modified by adding a value 0.5 to the calculus (see Methods, supplementary text S1 [see additional file 1]). We focus on the magnitude of reported p-values, as the selection of significant classes would depend strongly on the used multiple testing correction.

**Figure 5:**
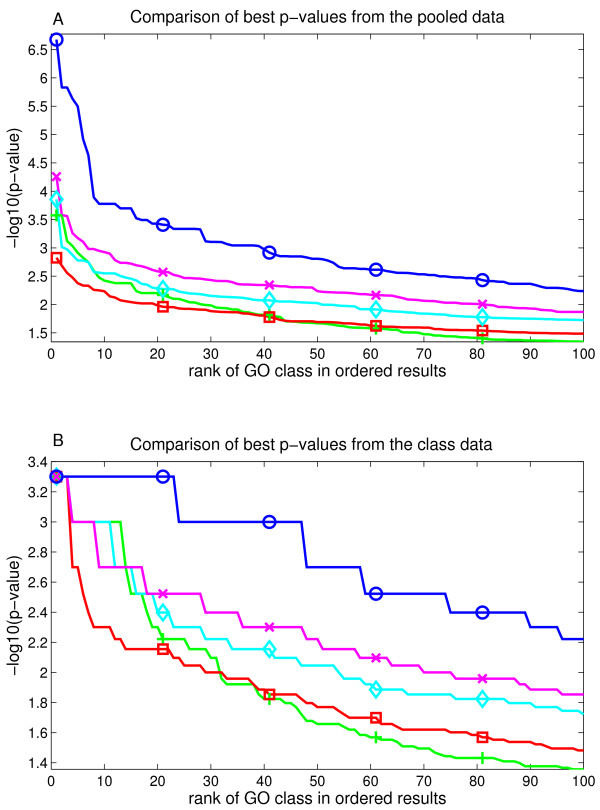
**Visualization of the empirical log-p-values from ALL dataset for the top-100 classes of each scoring function**. Part A shows the obtained p-values when all class randomizations are normalized and pooled. Part B shows the results when each class is analyzed separately. Largest value in lower plot refers to p-value = 0 (see main text for details). Lines are: blue with circles = GSZ-score; green with cross = t-test; red with squares = KS; magenta with x = iGA; cyan with diamonds = modKS. Notice that GSZ-score shows here a very clear separation from the other methods in both of the plots. Any reasonable threshold would result in a larger number of significant classes with GSZ-score than with any other method.

The actual p-value signals are represented as two separate results in the fig. 5. The fig. 5A shows p-values from *pooled data*. Here each GO class is first normalized using the mean and the STD from the randomizations and next all the randomizations are pooled and used together to obtain p-values. Fig. 5B shows p-values from *class data*. These empirical p-values are obtained by using only the class specific randomizations with each class. Our analysis showed that these two log(p-value) results correlate strongly, and we further show in supplementary text S1 [see additional file 1] that our normalizations with randomized data was rather stable. Note that the class data has got a smaller intensity range and also discrete steps in the reported log-p-values, which is why it does not separate functions as well as pooled data.

Fig. 5 shows that the GSZ-score obtains significantly larger log(p-values) in the analysis of pooled log(p-values) (fig. 5A). The difference is surprisingly large with the best log(p-value) for GSZ-score being 6.7, whereas the second best method (iGA) has best log(p-value) 4.2. Furthermore, the difference is one to two orders of magnitude across the whole top class list (GSZ-score p-value is 10 - 100 times smaller than p-value of any compared method). GSZ-score also reports 23 classes with p-value = 0 with class data, whereas other functions report only 1 - 3 classes. These are represented by largest values in the fig. 5B. These observed differences are larger than any of the differences between the other methods in the comparison.

**Figure 6:**
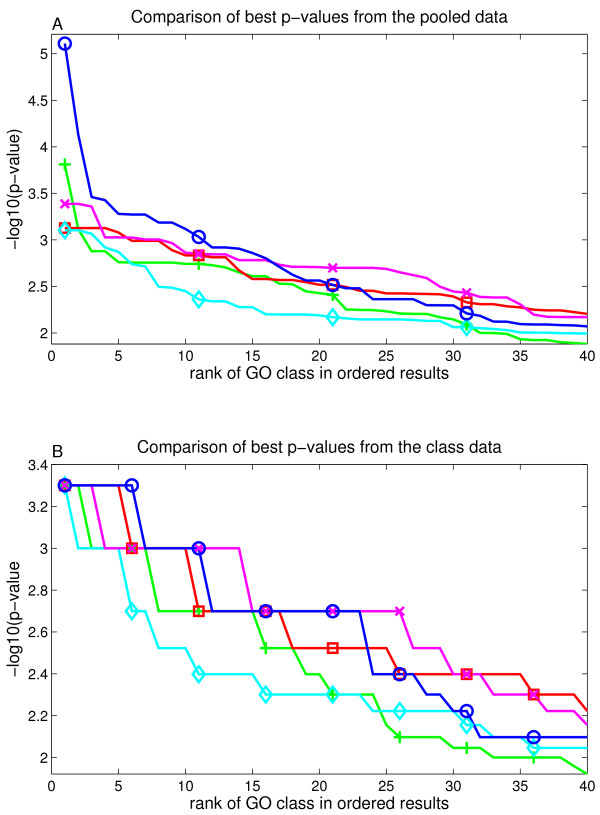
**Visualization of the empirical log-p-values from p53 dataset for the top-40 classes of each scoring function**. Part A shows the results when all class randomizations are normalized and pooled. Part B shows the results when each class is analyzed separately. Different functions are marked similarly to earlier figure. Notice that GSZ-score shows here a clear separation from the other methods in upper plot. In the lower plot it is the best performing method at top ranks and then as signal levels drops GSZ drops to 2^*nd *^best.

ALL dataset represents very strong biological signals. However, it is equally important to evaluate the scoring functions with expression data with weaker signal levels. Therefore, we replicated the p-value comparison with p53 dataset [13] and represent the results in fig. 6. Although the differences to other methods are now smaller, GSZ is clearly the best method in pooled data over top 15 classes. In class data the differences are smaller. GSZ produces again largest number of classes with *p-value *= 0 (6 classes), but also KS produces 5 classes. In addition, GSZ drops to the 2^*nd *^best after iGA around rank 10. This data proposes that GSZ is the best function across top ranks but it drops down later. These weaker ranks probably represent more random background signal.

### Biological comparison of class scoring functions

We also evaluate the biological relevance of the reported classes. This monitors the ability of scoring functions to report the relevant biological classes. We generated a sorted list of GO classes from each scoring function using the p-values from pooled data and class data in combination (see Methods, Supplementary text S1 [additional file 1]). This analysis is replicated on both real datasets, ALL and p53. The evaluation requires that we define functional classes that can be considered biologically positive, by selecting classes that can be linked with research setup. This selection is justified from the background biology, and it should not favour any of the evaluated scoring functions.

P53 dataset compares cancer cell lines with a mutation in p53 transcription factor to the ones without mutations. Here the classes with link to programmed cell death (apoptosis) were considered as positive. Selected classes were further confirmed by looking their regulatory relationship with p53 from the GeneGo Inc. MetaCoreTM database ([21], http://www.genego.com). As p53 data represents a small number of positive classes, we represent its results here in table 5. A more detailed presentation is in the supplementary table S1 [additional file 7]. Although most methods select the same apoptosis class (release of cytochrome c from mitochondria) as best, GSZ tops others by reporting 4 apoptosis related classes. GSZ reports also strongest p-values to each of these apoptosis related GO classes. KS and, unexpectedly, also T-test show here very weak performance.

**Table 5:** Comparison of scoring functions on p53 dataset

	GSZ	iGA	t-test	modKS	KS
1	*BP:0001836: release of cytochrome c from mitochondria*	*BP:0001836: release of cytochrome c from mitochondria*	CC:0009434: microtubule-based flagellum	*BP:0001836: release of cytochrome c from mitochondria*	BP:0050962: detection of light...
2	BP:0051668: localization within membrane	CC:0031903: micro-body membrane	MF:0033558: protein deacetylase activity	BP:0050953: sensory perception of light stimulus	BP:0050908: detection of light...
3	CC:0031903: micro-body membrane	CC:0005778: peroxisomal membrane	MF:0004407: histone deacetylase activity	BP:0007601: visual perception	BP:0007602: phototransduction
4	CC:0005778: peroxisomal membrane	CC:0044438: micro-body part	CC:0031903: micro-body membrane	CC:0000118: histone deacetylase complex	BP:0009584: detection of visible light
5	BP:0042787: protein ubiquitination... catabolic process	CC:0044439: peroxisomal part	CC:0005778: peroxisomal membrane	MF:0001664: G-protein-coupled receptor binding	BP:0009583: detection of light...
6	*BP:0008637: apoptotic mitochondrial changes*	BP:0042787: protein ubiquitination... catabolic process	CC:0019861: flagellum	BP:0015674: ditri-valent inorganic cation transport	MF:0016018: cyclosporin A binding
7	CC:0044438: micro-body part	BP:0050953: sensory perception of light stimulus	CC:0044438: micro-body part	MF:0033558: protein deacetylase activity	*MF:0005086: ARF guanyl-nucleotide...*
8	CC:0044439: peroxisomal part	BP:0007601: visual perception	CC:0044439: peroxisomal part	MF:0004407: histone deacetylase activity	*BP:0032012: regulation of ARF protein signal...*
9	CC:0009434: microtubule-based flagellum	MF:0033558: protein deacetylase activity	BP:0030890: positive regulation of B cell proliferation	BP:0006816: calcium ion transport	*BP:0032011: ARF protein signal transduction*
10	BP:0051205: protein insertion into membrane	MF:0004407: histone deacetylase activity	BP:0050962: detection of light...	MF:0019237: centromeric DNA binding	CC:0031903: micro-body membrane
11	BP:0046504: glycerol ether biosynthetic process	CC:0009434: microtubule-based flagellum	BP:0050908: detection of light...	MF:0030170: pyridoxal phosphate binding	CC:0005778: peroxisomal membrane
12	BP:0045017: glycerolipid biosynth...	MF:0016018: cyclosporin A binding	BP::0007602:: phototransduction	BP:0008015: circulation	MF:0033558: protein deacetylase activity
13	BP:0008643: carbohydrate transport	MF:0019237: centromeric DNA...	BP:0009584: detection of visible light	BP:0035136: forelimb morphogenesis	MF:0004407: histone deacetylase activity
14	*BP:0008629: induction of apoptosis by intracellular signals*	BP:0051205: protein insertion into membrane	CC:0000118: histone deacetylase complex	BP:0015918: sterol transport	CC:0031594: neuromuscular junction
15	*BP:0008635: caspase activation via cytochrome c*	BP:0046504: glycerol ether biosynthetic process	BP:0018298: protein-chromophore linkage	BP:0030301: cholesterol transport	MF:0005048: signal sequence binding

Biological classes reported by each scoring function from p53 dataset. Apoptosis related classes are highlighted with asterisk before and after the class name. Clear apoptosis classes are shown with bold font and border cases with underlined font. Although many methods report same apoptosis GO class as the top class, only GSZ is able to report three other apoptosis GO classes. Furthermore, our supplementary table S1 [see additional file 7] shows that GSZ reports strongest p-values for reported apoptosis gene sets.

**Figure 7:**
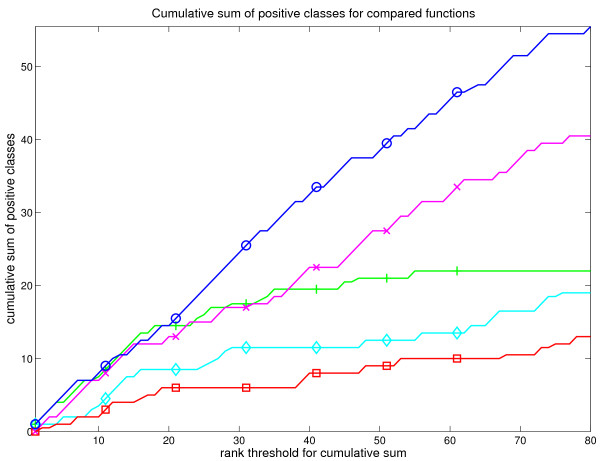
**Visualization of the cumulative sum of biologically positive classes among the top-80 classes for each scoring function**. Figure shows how many biologically positive classes each method discovers across their top ranks from ALL dataset. Different scoring functions are marked similarly to earlier figures. Notice that although GSZ, iGA and t-test first show equal performance, GSZ outweigh other methods across the later ranks. A more detailed view is provided in the supplementary table S2 [see additional file 8].

As ALL dataset compared B and T cell lymphomas with each other, we considered classes linked to immune response as positive. These include classes like immunoglobulin complex, T cell receptor complex etc. Two groups of classes were considered as border cases: the generic MHC protein complex and interleukin proteins. Due to the large number of positive classes, these results are summarized in fig. 7. Each positive class is weighted here as one and each border case classes were weighted as 0.5. More detailed presentation of the results is in supplementary table S2 [see additional file 8].

Fig. 7 shows that our ALL dataset represents a very strong biological signal with GSZ, t-test, and iGA exhibiting 11 - 12 positive classes among the top 15 classes. ModKS and KS clearly represent weaker performance with 7 positives and one border case (modKS), and 3 positives and 2 border cases (KS) among the top 15 classes. Outside the top ranks, the density of positive classes in t-test and iGA test drops, whereas GSZ clearly shows the best performance. Results from both ALL and p53 dataset propose that GSZ is outstanding among the evaluated scoring functions in finding relevant biological signals.

### Analysis of pairwise differences in class p-values

In order to evaluate further the differences between the scoring functions we focused on the pairwise differences in the log10(p-values) for each GO class between GSZ-score and each of the other methods. This allows the analysis of the actual GO classes representing the strongest separation between the compared methods. Here, again, we monitor whether these classes are relevant to research setup and also the difference in the log(p-values).

The outline of this analysis is represented in the supplementary text S1 [see additional file 1], and detailed results of the comparison in supplementary tables S3 and S4 [see additional file 9 and 10]. The top classes, where GSZ outweighs other methods are mostly all positive classes. Also the differences in p-values, favouring GSZ, are very large. The opposite classes are more or less random selections from the GO classes.

Also p-value differences are much smaller. There are also some interesting details in the comparisons:

#### GSZ vs. T-test

When comparing GSZ with T-test we observe large number of relevant GO classes with simultaneous up and down-regulation (large STD signal, small mean signal) in ALL dataset. These were naturally missed by T-test, whereas GSZ was able to report them. Furthermore, we observed surprisingly weak performance of T-test on p53 dataset.

#### GSZ vs. iGA

The comparison with iGA showed many classes from ALL dataset that had a strong regulation. GSZ was able to report very strong p-values for them, whereas iGA did not notice them since it discards the expression values. This underlines that inclusion of differential gene expression scores benefits the test statistic. GSZ clearly reported from p53 dataset better p-values for 3 apoptosis classes.

#### GSZ vs. modKS

On ALL dataset modKS shows very strange behaviour, and it is clearly outperformed by GSZ. Our assumption is that weak performance of modKS is related to its sensitivity to outlier expression values in GO classes. This outlier sensitivity is further demonstrated by supplementary figures S1 and S2 [see additional file 3 and 4].

#### GSZ vs. KS

Standard KS showed weakest performance in these comparisons reporting mostly quite random GO classes.

These results bring forth GSZ as an outstanding method, when evaluating the biological relevance of the reported classes. There was only one exception on this rule in the p53 dataset, where iGA and KS reported stronger signal on ARF protein signalling classes. These apoptosis classes were not reported as linked to p53 according to GeneGO. Also their average regulation was very small.

### Comparison of GSZ with program packages

We also compared our analysis pipeline with other actual software packages. This comparison is less clear to interpret, due to a large number of variables between different analysis pipelines. In addition, the programs report p-values post-processed in various ways. Therefore, the evaluation is focused on the order of the reported GO classes omitting the actual p-values. These obtained GO class lists are evaluated using the selected biologically positive classes again. We selected three software packages for comparison. These were Gene Set Enrichment Analysis package (GSEA, R code version [13]), Signal Pathway (SP [9]) and Gene Set Analysis (GSA [15]).

All compared methods were run with the same set of GO classes, with no maximum size limit and the minimum size limit set to 3. Although these settings are sub-optimal for the biological analysis, they allow a thorough evaluation of the packages against the variations in the GO class size. All the methods were also tested with 1000 randomizations. Both these settings correspond to the parameters used with the GSZ analysis in function comparison.

Two methods (GSEA and GSA) are one-sided tests, generating two separate outputs. However, we need a single GO class list for comparison with SP and GSZ-score. This was accomplished by combining the two GSEA output lists and sorting them using the absolute value of Normalized Enrichment Score (NES), used also to sort the result classes in the GSEA. With GSA we did a similar procedure, ordering in the first round with the normalized score (calculating Z-scores with permutation results, returned by GSA) and in the next round using the p-values. However, we also discuss the rankings in the separate lists. The following two chapters represent the results from two datasets.

#### ALL dataset

**Figure 8:**
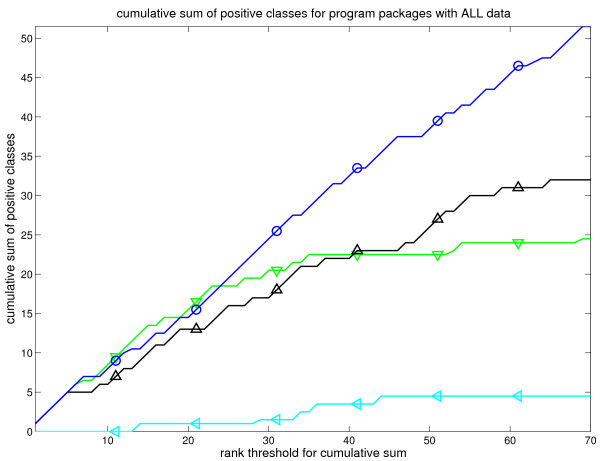
**Visualization of the cumulative sum of biologically positive classes among the top-70 classes for GSZ and compared programs**. Figure shows how many biologically positive classes each method discovers across their top ranks. Blue line with circles denotes GSZ. Green line with triangle downwards denotes SP. Black line with triangle upwards denotes GSA. Cyan line with triangle to left denotes GSEA. Notice that although GSZ, GSA and SP first show equal performance, GSZ outperforms other methods across the later ranks. A more detailed view is provided in the supplementary table S5 [see additional file 11].

First we compare methods with ALL dataset. We used same biologically positive classes as in the earlier evaluation with the ALL dataset. The obtained results can be seen in the supplementary table S5 [see additional file 11] and the summary in the fig. 8. The first observation is that three out of four methods clearly report positive classes. These are GSZ, SP, and GSA. These results are in agreement with the strong signal that was reported from this dataset in the earlier comparison of scoring functions. GSEA, nevertheless, shows a strong deviation from this rule. None of the reported classes was able to obtain stronger signal than its default cutoff (False Discovery Rate ≤ 0.25). Furthermore, the reported classes are quite arbitrary with only 4 positive classes among the top-70 classes. This is a very strong disagreement between GSEA and all the other evaluated methods.

There are also differences between the top three methods. GSZ, GSA, and SP all have only a few negative classes among top 20 classes. However, after the rank 20 their density drops in GSA and SP results. Indeed, there is only 7 negative classes among top 40 classes in GSZ-score results and one border case, whereas SP results have 17 negative classes and one border case, and GSA has 18 negative. This makes their error rate among these top classes over two times bigger and highlights GSZ again as the best method. A detailed analysis shows that SP misses totally positive classes with large variance signal and small mean signal (thymic T-cell related classes and immunological synapse). Likewise, GSA represents a quite small signal for some classes emphasized by SP and GSZ, like 'MHC class II protein complex' and 'antigen prosessing and presentation' classes.

#### P53 dataset

Next, we compared the methods with p53 dataset. Here, again, we select the programmed cell death (apoptosis) related GO classes as positive. Obtained results are shown in table 6. More detailed results are shown in supplementary table S6 [see additional file 12]. Again, GSZ shows good performance with 4 apoptosis classes among its top-15 results, showing strong ranking positions, such as 1 and 6.

**Table 6:** Comparison of GSZ and program packages using p53 dataset

	GSZ-score	GSA	SP	GSEA
1	*BP:0001836: release of cytochrome c from mitochondria*	CC:0031903: micro-body membrane	CC:0009434: microtubule-based flagellum	CC:0000118: histone deacetylase complex
2	BP:0051668: localization within membrane	CC:0005778: peroxisomal membrane	MF:0005125: cytokine activity	BP:0009314: response to radiation
3	CC:0031903: micro-body membrane	CC:0009434: microtubule-based flagellum	CC:0019861: flagellum	BP:0050962: detection of light...
4	CC:0005778: peroxisomal membrane	CC:0044438: micro-body part	BP:0050962: detection of light...	BP:0050908: detection of light...
5	BP:0042787: protein ubiquitination...	CC:0044439: peroxisomal part	BP:0050908: detection of light...	BP: 0007602: phototransduction
6	*BP:0008637: apoptotic mitochondrial changes*	*BP:0001836: release of cytochrome c from mitochondria*	BP:0007602: phototransduction	BP:0009584: detection of visible light
7	CC:0044438: micro-body part	CC:0005626: insoluble Fraction	BP: 0009584: detection of visible light	BP:0009583: detection of light...
8	CC:0044439: peroxisomal part	CC:0019861: flagellum	*BP:0001836: release of cytochrome c from mitochondria*	MF:0030170: pyridoxal phosphate binding
9	CC:0009434: microtubule-based flagellum	MF:0015103: inorganic anion transmembrane...	BP:0009583: detection of light...	*BP:0001836: release of cytochrome c from mitochondria*
10	BP:0051205: protein insertion into mem brane	BP:0051668: localization within membrane	BP:0006955: immune response	MF:0005487: nucleocytoplasmic trans porter activity
11	BP:0046504: glycerol ether biosynthetic process	BP:0050962: detection of light...	BP:0030890: positive regulation of B cell proliferation	BP:0006654: phosphatidic acid biosynthetic process
12	BP:0045017: glycerolipid biosynthetic process	BP:0050908: detection of light...	MF:0001664: G-protein-coupled receptor binding	BP:0046473: phosphatidic acid metabolic process
13	BP:0008643: carbohydrate transport	BP:0007602: photo-transduction	BP:0006572: tyrosine catabolic process	BP:0035137: hindlimb morphogenesis
14	*BP:0008629: induction of apoptosis by intracellular signals*	BP:0009584: detection of visible light	MF:0008009: chemokine activity	BP:0051716: cellular response to stimulus
15	*BP:0008635: caspase activation via cytochrome c*	CC:0031594: neuromuscular junction	MF:0042379: chemokine receptor binding	BP:0008217: blood pressure regulation

Table compares GSZ analysis with three popular software packages. Apoptosis related positive classes are highlighted as in table 5. GSZ, GSA and SP reported classes with strong signal, whereas GSEA did not report any significant classes. GSZ, GSA and SP reported strong p-value for GO class 1836, but only GSZ ranks it as top class. GSZ also reports 3 other apoptosis related classes.

SP reported one apoptosis related class at the rank 8, with empirical p-value = 0. This was the same class rated as first class by modKS, iGA and GSZ. It had actually the first rank in the column randomization of SP, proposing that it is here more useful than the combined ranking from two randomizations. Thus, SP performs quite nicely, but reports only one cell death related class among the top-50 classes, whereas GSZ was able to report 4 of them among top-15 classes. Furthermore, the standard ranking of SP was not optimal here.

GSA generates separate lists for up and down regulated classes. Also, the outputs of GSA represent several GO classes with same p-values. Therefore, we generated new ordering for GO classes, separately for up-regulated, down-regulated and combined set of GO classes. One apoptosis related class is seen in the down-regulated set of GO classes with empirical p-value = 0. Its original rank was 5^*th *^and after our reordering with Z-scores its rank is improved to 2^*nd *^position. However, when the two GO class lists are pooled, the rank of the apoptosis class is only 6. Therefore, its performance seems to be weaker than GSZ.

GSEA generates two lists, instead of one. We again combined them into a single list. The apoptosis related classes were in the list of down-regulated classes at the ranks 8, 14 and 24, and in the results of the pooled list at the ranks 9, 21 and 37. However, if we were to use the default GSEA FDR cutoff (0.25), none of these classes would have been reported. Also, all these apoptosis related classes were low in the result ranking. Therefore, the performance of the GSEA seems quite weak again.

Altogether, both datasets point out to GSZ as best method for detecting biologically relevant classes. SP and GSA show quite equal second best performance. Furthermore, both datasets pinpoint GSEA as the apparently the weakest method.

### Biological analysis of results

GSZ reported 4 apoptosis related classes from p53 data: Release of cytochrome c from mitochondria, apoptotic mitochondrial changes, induction of apoptosis by intracellular signal and caspase activation via cytochrome c. What these classes represent is some of the key steps for programmed cell death: intracellular apoptotic signalling to mitochondria, removal of cytochrome c and its later complex formation with caspase. In addition, other methods (iGA and KS) highlight the ARF protein signal transduction. This group plays a relevant role in apoptotic signalling, although it shows here only very weak regulation. This suggests that a combination of two or three scoring functions could actually highlight all the possibly relevant biological signals in the dataset.

ALL dataset showed classes representing simultaneous up and down-regulation, such as immunological synapse, positive thymic t cell selection and lymphocyte activation. Key trends here seem to be: Cell surface receptor signalling that activates immune response and developmental steps of T cells in thymus. Both these processes are central to immune processes. GSZ is able to recognize in particular the immunological synapse, a communication method between B cells and T cells. Note that part of the immunological synapse is expressed in T cell and part in B cell and it is complete when two cells interact with each other. These relevant immunological processes could not be observed from this dataset by methods that monitor only the mean expression of the GO class. Here t-test and SP missed them. Only GSZ and iGA performed satisfactorily on these classes. Also GSA from program comparison was able to detect a fraction of these.

ALL dataset also represented GO classes with strong mean up or down-regulation. These classes link to T cell receptor complex, antigen processing via MHC class II protein complex, and to MHC class II protein complex. What is paradoxical is that some methods did not give good scores to these classes. Especially iGA and KS performed weakly with these. This was probably due to the limitation of the analysis only to the order of the genes. Only GSZ and t-test in the comparison of scoring functions reported good scores for these classes. Also SP gave very strong signals to these classes. Surprisingly, GSA obtained less strong results on some of these classes (MHC class II protein complex and antigen processing related classes). Altogether, these results point that across different biologically relevant signals GSZ shows better performance compared to the other, competing algorithms and programs.

## Discussion

Current work proposes a novel class scoring function for threshold free gene set analysis. The function is a Z-score that is shown to have similarities to hypergeometric function and to correlation (see Results and supplementary text S2 in [see additional file 2]). Gene Set Z-score is shown to be stable under row randomization to variations of subset and class sizes. Furthermore, it can be stabilized with column randomizations against the biases caused by gene-level correlations. GSZ-score had the best overall performance in the artificial data analysis. The second best class scoring function was iGA. It is, however, significantly heavier to compute than the GSZ-score. In addition, we have performed a detailed evaluation on a real dataset, where the number of positive classes and the way how they were selected was altered. Here, GSZ-score was consistently the best performing class scoring function, whereas the performance of other functions varied. A further proof favouring GSZ-score was obtained when analyzing the empirical p-values from two datasets. Here GSZ-score was the top method, especially with ALL dataset showing 10 - 100 fold better p-values across the top hundred classes. Altogether, these numerical results indicate that the GSZ-score has an outstanding performance, when compared to the other represented class scoring functions.

We also monitored the biological performance of scoring functions by looking how they reported relevant classes. This evaluates the scoring functions for explorative biological comparison of sample groups. GSZ-score showed best performance, reporting biologically relevant classes with different signal types from ALL dataset. Other scoring functions showed weaknesses, discussed in the background. From p53 dataset only GSZ-score reports four relevant apoptosis related GO classes.

Biological analysis was also replicated against three widely used gene set analysis packages: Gene Set Enrichment Analysis, Gene Set Analysis and Signal Pathway. GSZ-score clearly showed better performance in biological explorative analysis also against these tools. We omitted any detailed evaluation of p-value signal between the program packages, since there is large number of variables between packages making comparison difficult. However, we point that GSZ-score, GSA and SP show strong p-values, whereas GSEA shows very weak signals.

It should be noted that we are excluding from the scoring function evaluation all the other (critical) analysis steps, and treat the functions in a consistent manner. This potentially weakens the performance of modKS, for which the original article [13] reported the need of separate evaluation of the up-regulated and down-regulated tail of the expression dataset. Also, the original work used a correlation test for differential expression, and used a different normalization with randomized data (for KS and modKS). These were excluded, as our aim was to keep the evaluation of the methods as constant as possible. Varying also other analysis steps would make it difficult to interpret what actually caused the differences in the results. Furthermore, our biological analysis shows better performance for modKS in our analysis pipeline than what is observed in GSEA, indicating that our analysis pipeline performs better.

Most earlier publications focus only on one of the two potential null permutations. We propose a separate normalization with row and column randomizations of a dataset, and the selection of the less significant outcome (normalized score value or p-value) as the result. This represents a pessimistic but also intuitive perspective, where the more likely null model is allowed to explain the observed results. As a drawback, the obtained p-value will naturally be conservatively biased. Nevertheless, our permutation evaluations show that GSZ is stable under row randomizations (see supplementary text S1 [additional file 1]). Also Efron and Tibshiriani [15] have proposed an alternative way to combine two nulls.

There are some details that were observed to cause problems when comparing gene expression data with functional classifications. These problems probably occur in many other biological data comparisons as well. A dataset should have a single, explicit representative for a single gene/transcript. Several measurements strongly violate the independence assumption, utilized by all the popular methods that we are aware of. Therefore, one can obtain seemingly highly significant GO class results when several measurements are associated with a single false positive gene. Similarly, it is unclear how to treat measurements (probe sets) that are associated with many genes. Fortunately, the current remapping projects (see for example [22]) aim to solve these types of problems, and turn out to be crucial for any gene expression data analysis. Furthermore, we have observed that genes not linked to classification should be filtered from the analysis. These are often classified as not belonging to the class, but such a solution caused false positive signal amongst the very largest classes of GO structure. We want to point out that our ALL dataset has been preprocessed to take these details into account.

Randomizations play critical role in analysis (see supplementary text S1, [additional file 1]). Row randomizations revealed substantial bias in some scoring functions (KS, modKS, normal t-test) and slight bias in iGA. Even more biased results were observed when doing the column randomization. Especially the iGA showed a dramatic bias here (largest -log_10_(p-value)>70). Note that iGA represents a variation of the standard threshold based hypergeometric analysis, and these results suggest that one might need similar column randomization also with those methods [19]. Also some gene set analysis methods currently omit column randomization totally [7,11,14], which might cause bias in their outcome.

The evaluation of gene set enrichment type analysis methods is a non-trivial task, and its problems are not usually discussed. We can design testing with artificial data where we know the positive and negative classes. However, the relevance of different signal types is not known. With real dataset we have an opposite problem: We have a true signal across different classes, but the actual classification to positive and negative classes can be considered to be a subjective and vague decision. Here, we have first used the same methods that we are evaluating for the selection of the positive classes, but point out that this is not an optimal solution. Furthermore, we propose that it is reasonable to perform the testing so that the threshold for the positive class selection is allowed to vary. This enables the testing with varying number of positive classes for monitoring consistently good performance. Also the definition of the positive classes is varied. One can start with an assumption that the results from the method itself, from a similar dataset, represent the optimal solution that the method is then allowed to predict (referred sometimes to as concordance analysis. See similar work in [15]). The use of two datasets would correspond to a real-life situation where one laboratory is trying to confirm the results from another laboratory. Previous evaluation omits the positive results from other methods, and therefore we decided to repeat the analysis using results pooled from several methods. These test situations would correspond to a situation where the second lab is confirming results from the first lab (with one method), by using many methods in combination. A further improvement to the presented analysis would be the testing with smaller subsets of the original data, to potentially see further variations in the method performances.

Real data was evaluated even further by generating a 1000 row and column randomizations, and looking at the actual p-values. We focused on the top 100-classes from ALL dataset where GSZ-score clearly represented a larger signal with 250 - 7100 times better signal for strongest class, and consistently over ten times better p-values for top ranks. Obtained results were further analyzed by looking at the biologically positive classes. Analysis showed that GSZ-score results show largest number of biologically relevant classes among their top ranks. Scoring functions were further compared using the pair-wise differences of the GO-class p-values. These differences from ALL dataset highlight *a*) classes where T-test fails due to the heterogeneous regulation, *b*) classes where iGA represents weak signal although the class represents a clear regulation, *c*) strongly regulated classes where modKS fails totally. P53 dataset gave also similar results. Overall, our work represents one of the most detailed evaluations of GO analysis methods with artificial and real data, and we hope to inspire the field to implement similar detailed and comprehensive evaluations in GO analysis method comparisons.

The combination of artificial and real data analysis revealed some exciting insights. KS test had the third best performance in the artificial datasets, but with real data it showed the undisputedly weakest performance. This seems to confirm the hypothesis that the signal area where KS shows its best performance in artificial data (see fig. 1 and 2) is unimportant in the real datasets [7,13]. Also modKS function showed very illogical behaviour with ALL dataset, preferring classes with smaller average regulation. Altogether, these results suggest that the use of KS and modKS should probably be avoided in GO class analysis. We observed even stronger differences in program package comparison. There GSEA package was indisputably the weakest program.

The introduced GSZ-score could be further improved. One of its weaknesses is the requirement of the prior variance *k*, which corrects for signal when the subset from gene list is small. However, our artificial data evaluation shows set of parameter values that represent equally optimal performance (*w*1 = 0.1, 0.2 and *w*2 = 0.3, 0.5). These are used to add prior variance to calculus. Similar method is regularly used in gene expression data analysis and in various areas of data mining (like Ridge Regression, bias-variance tradeoff or addition of pseudo counts to the calculus). Yet the performance could potentially be improved with a more exact measure using an estimated cumulative distribution (for more exact p-value) or Bayes Factor. In addition, the performance might be easily improved by using a larger number of score values from the calculated GSZ-score profile, obtained over the gene list, than just a single maximum value. A separate variation on this theme is the connection to max-mean statistic [15], which was observed to correspond to GSZ-score obtained when the threshold, selecting the analyzed subset, is set to correspond zero in differential expression scores (see supplementary text S2, [see additional file 2]). The presented GSZ-score should be applicable also to other relevant research topics, such as feature extraction. In addition, the mathematical derivation of the GSZ-score should provide an easy platform for further modifications.

## Conclusion

With increasing demand of gene list data analysis for gene expression, the introduced Gene Set Z-score function should represent a significant addition to analyst's tool palette. It represents a novel addition of signal-levels to ranked list analysis and gives reliable results.

## Methods

### Estimates for the expected value and the variance of GSZ-score

To calculate the estimates for the mean and variance, we have to note that the analysis situation can be modelled with a composition of two functions. The first one defines the probability for the observed number of class members in the analyzed subset. The second one defines the probability for obtaining the observed sum for the class members. The exact analysis with Bayes factors or using exact cumulative distribution to calculate p-values, would require the definition of a complex distribution with potentially too heavy calculations. Therefore, we prefer to use a Z-score with relatively simple estimates for mean and variance. Mean is simpler to derive from these two, and it is also required in the variance estimate. Thus we represent it here first. We start with the difference(8)

Here *pos *refers to positive genes (class members) and *neg *refers to negative (class non-members). We are dealing here with a sum of *N *values *S *= Σ_*n*_*X*_*n *_selected from the pool of *X*_1_, *X*_2_,..., *X*_*M *_values, which in turn is a subset of the total dataset (with size of *L *genes with *K *positive genes). The *N *= *N*_*pos *_is the number of positive genes included to our data subset (with *M *= *N*_*neg *_+ *N*_*pos*_). When the subset is randomly sampled from the total data pool, we do not know the *N *beforehand, but it can be expected to follow hypergeometric distribution, representing sample (without replacement) of size *M *from the pool of *L *genes including *K *genes classified as positive. Expected values for both terms in the eq. (8) can be defined using conditional probabilities(9)

which is simply the probability weighted sum of expected values, conditional on the number of class members in the sum. Proof that the *E*(*S*|*N *= *i*) = *iE*(*X*) is shown in supplementary text S1 [see additional file 1]. *E*(*X*) represents the expected value, or mean, of the data subset *X*_1_, *X*_2_,..., *X*_*M*_. Expected value for eq. 8 can be represented as:(10)

So the expected value is a simple function of expected value for hypergeometric distribution *E*(*N*), expected value for the test scores in the analyzed subset *E*(*X*) and the number of data points in the subset *M*. In practice we replace the *E*(*X*) with empirical mean calculated from the whole subset. This intuitive result is conditional on the selected data subset (M), on the size of the whole data pool (L) and on the number of positive genes in the whole pool (K). Estimate of the variance is somewhat harder to obtain. Here we start with the definition of variance for a single summation (*S*) in eq. (8).(11)

By fixing *N *we can express the expectation *E*(*S*^2^|*N*) as a sum of variance and the squared expectation.(12)

The first term in the latter equation represents the variance of the sum of *N *values selected from the pool of *X*_1_, *X*_2_,.. *X*_*M *_values with variance *D*^2^(*X*) (which is again replaced by empirical estimate obtained from the subset). Note that *D*^2^(*S*) = 0, when *N *= 0, *N *= *M *or if *X*_*i *_= *X*_*j *_for all values of *i *and *j *(resulting to *D*^2^(*X*) = 0). Derivation of *D*^2^(*S*|*N*), used in eq. 12 is represented in the supplementary text S1 [see additional file 1]. Substituting eq. 12 to the eq. (11) we get(13)

This is simply an equation of the variance and the mean of hypergeometric distribution (*E*(*N*), *D*^2^(*N*)), the mean and the variance for the subset of the data (*E*(*X*), *D*^2^(*X*)) and the size of the data subset (*M*). The result stays same, whether we monitor the variance of sum for the negative or the positive data points. Equation is similar to the well known equation for the variance of the sum of *N *copies of identically independently distributed variables *D*^2^(*S*_*iid*_) = *D*^2^(*X*)*E*(*N*) + *E*(*X*)^2^*D*^2^(*N*). Here the difference is caused by various draws of *X*_*i *_not being independent.

The equation (13) represented the variance of the first summation in the equation (12). For the whole variance of the eq. (8), we have to multiply eq. (12) with squared constant 2^2 ^= 4. Furthermore, we have to consider the variance of the latter term *ME*(*X*). A closer look reveals that this is simply a multiplication of two constants having no variance at all, no matter what outcome we observe in our test dataset.

Therefore it does not affect our variance estimate. So the final score for a data subset analysis becomes:(14)

In contrast to KS and modKS, when OGL is divided to two subsets, our score gives a different score for upper and lower subsets. Therefore, with eq. 14 it is required to consider separately the lower and upper end of the list. We took the largest absolute score from these two lists as the final outcome. Another modification to our score function was made due to the observed instabilities with small subset and class sizes. This is simply a prior variance *k *added to the variance:(15)

Note that similar procedure is regularly used with t-test in the expression data analysis. Selection of the prior variance will be discussed in the results section.

### Summary of the remaining Methods

The BioConductor package [23], probe remapping [22], Robust Multi-array Average (RMA, [24]) and Intensity Based Modified T-test (IBMT, [18]) were used in the gene expression data analysis. Gene ID-Converter [25] and Protein Information Resource (PIR, [26]) were used in the generation of GO dataset. The rest of the Methods are shown in the supplementary text S1 [see additional file 1].

## Abbreviations

ALL: Acute Lymphatic Leukaemia; AUC: Area Under receiver-operating characteristics Curve; ECD: Empirical Cumulative Density function; EVD: Extreme Value Distribution function; GO: Gene Ontology; GEVD: Generalized Extreme Value Distribution function; GSA: Gene Set Analysis; GSEA: Gene Set Enrichment Analysis; IBMT: Intensity Based Modified T-test; iGA: iterative Group Analysis; KS: Kolmogorov-Smirnov test; LR: Likelihood Ratio; ML: Maximum Likelihood; modKS: modified Kolmogorov-Smirnov; OGL: Ordered Gene List; RMA: Robust Multi-array Average; SP: Signal Pathway.

## Authors' contributions

PT developed, implemented and tested the methods. PO proposed the research topic and contributed to the datasets and visualization. PM contributed additional mathematical proofs. LH supervised this research. PT, PO and LH evaluated results. All contributed to the writing of the manuscript. All authors read and approved the final manuscript.

## Supplementary Material

Additional file 1 **Supplementary text S1**. This text shows in detail the stability of the GSZ-score and competing methods with randomized data, as the threshold moves through the gene list. In addition, the stability of the methods is monitored as a function of the GO class size. Text also highlights the false positive signals seen with column randomizations. Furthermore, the performance of the used normalization with randomized datasets is shown, and the normalized scores and the empirical p-values, obtained with row and column randomizations, are compared to each other. The text also represents the detailed comparison of pairwise differences in p-values for different scoring functions. In addition, most of the Methods are introduced here. Mathematical supplement shows the derivation of *E*(*S|N*) and *D*^2^(*S|N*), required for the derivation of the GSZ-score. Click here for file

Additional file 2 **Supplementary text S2**. This text represents a mathematical comparison of the GSZ-score with hypergeometric Z-score, with correlation between the differential expression scores and GO classification, with max-mean score (by Efron & Tibshiriani) and with Random Sets scoring function by Newton et al. Click here for file

Additional file 3 **Supplementary figure S1: Stability of the GSZ-score as the threshold goes through the gene list**. Distribution of the GSZ-score values as the threshold is moved along the gene list. Subset is smallest at the left and largest (the whole gene list) at the right end of the plot. Results are calculated using all the 4511 GO classes from diabetes dataset with randomized GO class matrix. Blue lines show seven percentiles (0, 5, 25, 50, 75, 95, and 100) at each position. For comparison, the red line shows minimum and maximum scores from the non-randomized diabetes dataset. Notice the good stability with the regularized GSZ-scores. Figure is discussed more in a more detail in the supplementary text S1 [see additional file 1]. Click here for file

Additional file 4 **Supplementary figure S2: Stability of KS and modKS as the threshold goes through the gene list**. Behaviour of the KS test and the modKS test with the same randomized and positive dataset. Lines represent the same percentiles as in the supplementary figure S1 [see additional file 3] with the same colouring. Notice the biases between different threshold positions, especially when the results are compared with the earlier supplementary figure S1 [see additional file 3]. Notice also the less clear separation between the negative and the positive dataset for modKS in the lower figure. Click here for file

Additional file 5 **Supplementary figure S3: Performance comparison in case (i) with each split analyzed separately**. Performance of the methods in each split in case i. Figure represents the AUC score for each evaluated method as the rank limit of the positive GO classes is increased. Note that AUC is calculated here using the whole evaluated GO class list, and it is the size of the used positive GO class set that varies. Methods represented are GSZ-score: blue line with circles, t-test: green line with cross, KS test: red line with box, modKS test: cyan line with diamond, iGA: magenta line with x. Notice that although the signal levels vary between the replicates, the differences between the methods are stable. GSZ-score and t-test show equal performance among the smallest ranks, whereas the GSZ-score is clearly the best among the larger ranks. Other methods show weaker signal. Figure is zoomed to the upper signal area so that most of the curve for the KS is left outside. Click here for file

Additional file 6 **Supplementary figure S4: Performance comparison in case (ii) with each split analyzed separately**. Performance of methods in each split in the case ii. The figure represents the AUC score for each evaluated method as the rank limit of the positive GO classes is increased. Methods are coloured identically to the earlier figure. Here, GSZ-score and iGA show the best performance at very top ranks, with GSZ-score slightly surpassing iGA. Note that performance of t-test and modKS varies considerably across the replicates. After rank 20 GSZ-score is constantly best in all the replicates. Click here for file

Additional file 7 **Supplementary table S1: Top 100 GO classes reported by each scoring function from p53 dataset**. This table presents 100 best scoring classes for each scoring function from p53 dataset. Table shows class rank, two combined empirical log-p-values, mean of expression values of GO class genes, mean of absolute expression values for GO class genes, class size and class name. In addition, we show four different empirical log-p-values and 7 percentiles (0, 5, 25, 50, 75, 95, 100) for the expression values of the class members. Empirical log-p-values are the ones used to calculate the combined log(p-value). Biologically relevant (positive) classes are shown in bold font and border cases are with underlined font. Results from each method is presented as a separate Excel worksheet. Furthermore, the first worksheet represents a summary of the results for each scoring function. Click here for file

Additional file 8 **Supplementary table S2: Top 100 GO classes reported by each scoring function from ALL dataset**. This table presents 100 best scoring classes for each scoring function from ALL dataset. Columns are similar to the columns in supplementary table S1 [see additional file 7]. Again each method's results are on separate sheet and the first sheet represents the summary. Click here for file

Additional file 9 **Supplementary table S3: GO classes showing largest pair-wise differences from ALL dataset**. This table presents 50 GO classes with the strongest combined log(p-value) differences in favour, as well as against the GSZ-score, in a pair-wise comparison with other scoring functions. Each comparison with each competing scoring function is on a separate sheet. Columns in the tables are identical with columns in tables supplementary table S1 and S2 [see additional files 7 and 8]. Click here for file

Additional file 10 **Supplementary table S4: GO classes showing largest pair-wise differences from p53 dataset**. This table presents 50 GO classes with the strongest combined log(p-value) differences in favour, as well as against the GSZ-score, in a pair-wise comparison with other scoring functions. Each comparison with each competing scoring function is on a separate sheet. Columns in the tables are identical with earlier tables. Click here for file

Additional file 11 **Supplementary table S5: Top GO classes from GSZ-score and compared program packages from ALL data**. This table presents top classes obtained from GSA, SP, GSEA and GSZ-score. Summary of the results is on the first sheet. Separate sheets show each programs results with additional data. Furthermore, we represent two result sheets for scoring functions with one sided test (GSA, GSEA). One presents the one sided test results and the other presents the combined results. Click here for file

Additional file 12 **Supplementary table S6: Top GO classes from GSZ-score and compared program packages from p53 data**. This table presents top classes obtained from GSA, SP, GSEA and GSZ-score. Summary of the results is on the first sheet. Separate sheets show each programs results with additional data. Furthermore, we represent two result sheets for scoring functions with one sided test (GSA, GSEA). One presents the one sided test results and the other presents the combined results. Click here for file
