# Supplementary material for: Robust extraction of functional signals from gene set analysis using a generalized threshold free scoring function
Source: BMC Bioinformatics. 2009 Sep 23;10:307. doi: 10.1186/1471-2105-10-307 (PMC2761411; doi:10.1186/1471-2105-10-307)
Supplement: Additional file 1 — Supplementary text S1. This text shows in detail the stability of the GSZ-score and competing methods with randomized data, as the threshold moves through the gene list. In addition, the stability of the methods is monitored as a function of the GO class size. Text also highlights the false positive signals seen with column randomizations. Furthermore, the performance of the used normalization with randomized datasets is shown, and the normalized scores and the empirical p-values, obtained with row and column randomizations, are compared to each other. The text also represents the detailed comparison of pairwise differences in p-values for different scoring functions. In addition, most of the Methods are introduced here. Mathematical supplement shows the derivation of E(S|N) and D2(S|N), required for the derivation of the GSZ-score. [file 1471-2105-10-307-S1.PDF]

Robust universal scoring function for threshold free gene set analysis: Supplementary text

Petri Törönen *[1], Pauli Ojala[2] , Pekka Marttinen[3], Liisa Holm[14]

[1]The Holm Group, Biocenter II, Institute of Biotechnology, PO Box 56, 00014 University of Helsinki, Finland
[2]Finnish Red Cross Blood Service Research and Development, Kivihaantie 7, 00310 Helsinki, Finland
[3]Department of Mathematics and Statistics, P.O. Box 68, 00014 University of Helsinki, Finland
[4]Department of Biological and Environmental Sciences, P.O. Box 56, 00014 University of Helsinki, Finland

Email: Petri Toronen*- firstname.lastname@helsinki.fi; Pauli Ojala - firstname.lastname@bts.redcross.fi; Pekka Marttinen - firstname.lastname@helsinki.fi; Liisa Holm - firstname.lastname@helsinki.fi;

*Corresponding author

Abstract

Short note: This is a supplementary text for the manuscript with the same title. The additions are the parts that handle stability under randomizations, pairwise p-value comparison of GSZ with other tested scoring functions, the most of the materials and methods, and derivation of $E(S|N)$ and $D^2(S|N)$ required for the derivation of the GSZ-score. This text represent five major findings: (*a*) Stability of the regularized GSZ-score over different threshold positions and instability of the KS and the modKS over the same threshold positions, (*b*) stability of the GSZ-score over different class sizes in row randomizations, (*c*) instabilities observed in the analysis methods when looking at the column randomizations, (*d*) stability of the results from the compared methods after using our normalization with randomized datasets, (*e*) better performance shown by GSZ-score in pairwise comparisons especially with biologically relevant classes.

Supplementary Results

Stability of various methods with row randomizations

In our preliminary analysis we concentrated on analyzing randomized real dataset with various methods to see how stable these methods are. We analyzed the stability against the variations in the size of the class with all the methods and the stability as the threshold goes through the gene list. Randomization was done by mixing the class labels (row randomization), and at this step we omit the contribution of the gene level correlations from the results. First we analyzed the stability of the test score as the threshold moves through the gene list. Aim is to see how stable various scores and also various regularizations for our GSZ-score are as the size of the subset is varied. We generated a single randomization of a Diabetes dataset [1]. We show these results only for the two best weight combinations and the GSZ-score without any regularization.

Results are shown in fig 1. Instead of showing all 4511 profiles for each GO class figure shows 7 percentile lines (0, 5, 25, 50, 75, 95 and 100), obtained from all the GO classes, at each threshold position. What draws first attention is the instability of the minimum and maximum value when the subset is very small, if no regularization is used. This can be explained by the Laplace distribution [2] reminding null distribution where the extreme values are quite far away although most of the probability mass is concentrated close to the null. This is unwanted behaviour especially when selecting the maximum value from score profile, and therefore our aim was to lessen it. Fig. 1 shows the results obtained with two regularizations. Now the distribution of minimum and maximum lacks extreme values with small subset sizes. Also quite reasonable range of weight values generates similarly stable results (data omitted). Notice also that the GSZ-score is not symmetrical so the score has to be calculated separately for upper and lower half at each threshold position. Last points at the right represent the signal obtained with whole gene list. This is where the original Diabetes dataset (red curves) shows strongest signals with regularized versions. This corresponds to the situation where the gene class shows consistent similar regulation, generating naturally a strong signal.

For comparison fig. 2 shows same randomization with two popular ranked list based methods. We show scores that are normalized with average (as recommended in [3], using 200 row randomizations). Here what especially draws attention is the instability of the null distributions of KS test based methods, although similar type of regularization could improve also these methods. Note that the same visualization could be obtained with hypergeometric test, but its calculation would be computationally extremely very heavy task

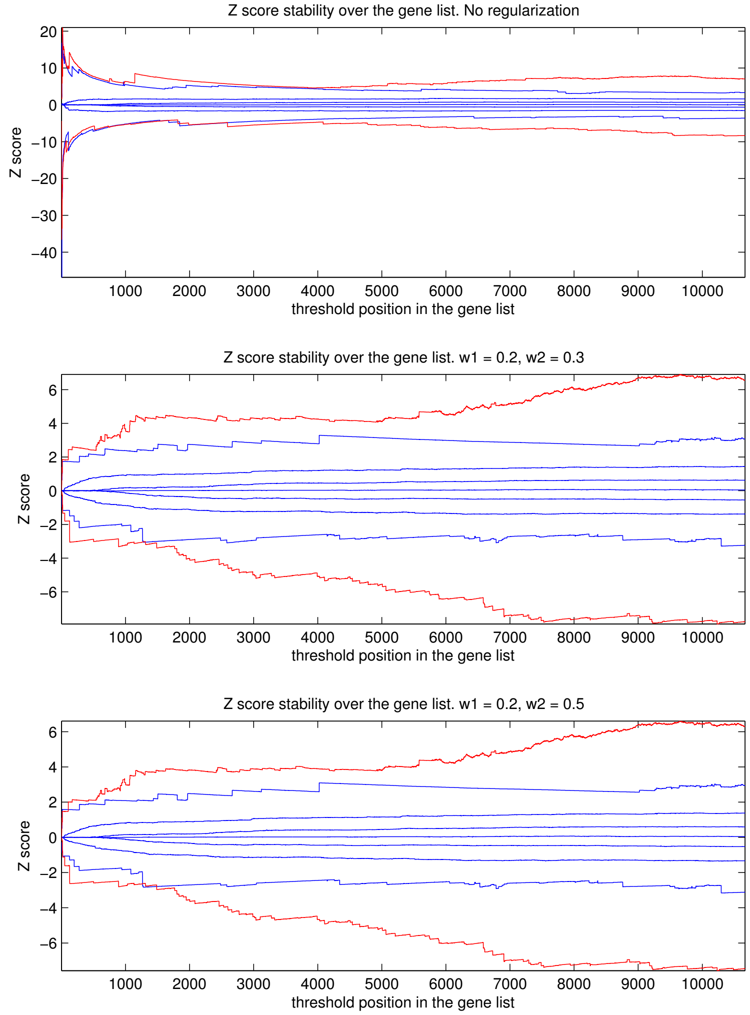

Figure 1: Distribution of GSZ-score values as the threshold is moved along the gene list. Subset is smallest at left and largest (whole gene list) at right. Results are calculated using all the 4511 GO classes from diabetes dataset with randomized GO class matrix. Blue lines show seven percentiles (0, 5, 25, 50, 75, 95, and 100) at each position. For comparison the red line shows minimum and maximum scores from the non-randomized diabetes dataset. Other positive percentiles are omitted as only a very small percentage of GO classes shows positive signal. Note the very unstable behaviour of GSZ-score without regularization, in the highest figure, and stable behaviour of regularized GSZ-scores everywhere except at the very beginning of gene list. This negative selection was still considered to be beneficial, as with very small subsets the effect of error in the signal increases. GSZ-score is referred as Z-score in this figure.

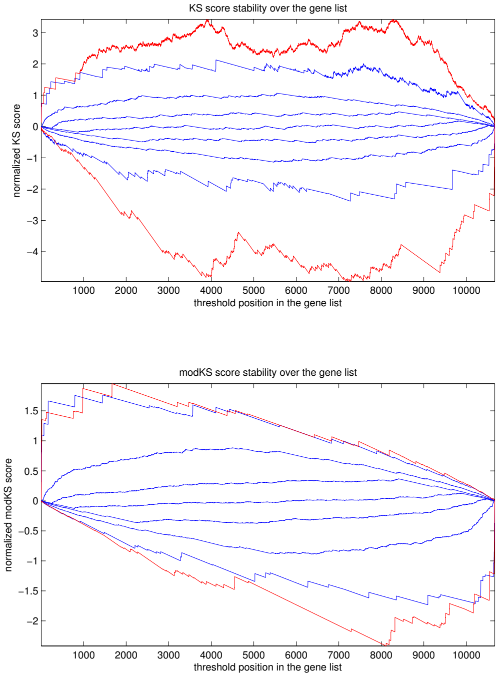

Figure 2: Behaviour of KS test and modKS test with the same randomized and positive dataset. Lines represent the same percentiles as in fig. 1 with same colouring. Now there are biases between different threshold positions, especially when the results are compared with earlier figure. Notice the less clear separation between the negative and positive dataset for modKS in the lower figure.

for every GO class at every threshold position (see materials and methods, on how iGA circumvents this). However, from our earlier research, we estimate the hypergeometric p-value to be quite stable (Törönen unpublished work).

Stability against varying class sizes was monitored by doing 200 row randomizations with each observed class size. Results are visualized against the log of the size of the class. Scores here were the maximum absolute values obtained from GSZ-score profile for each class over all the threshold positions. Figure shows only seven percentiles (0, 5, 25, 50, 75, 95 and 100). We exclude the results for the unregularized GSZ-score and show only the results obtained with two best-performing regularizations. The observed stability in fig. 3 is a surprise to us. Notice especially the stability of the 95 percentile in these figures. Only deviation is the slightly weaker median signal observed with smaller GO class sizes. These results propose that the regularized GSZ-score could be used to rough analysis even without the normalization with randomized results.

In addition to this we also show the stability of some other scoring methods. These results are shown in

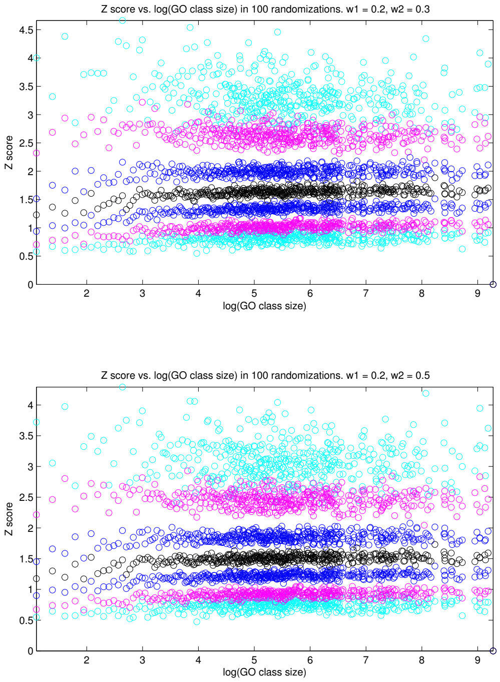

Figure 3: Stability of the two regularized GSZ-scores against the GO class size with 200 randomizations. Figure shows 7 percentiles with different colourings (0, 100: cyan; 5, 95: magenta; 25, 75: blue; 50: black). GSZ-score is referred as Z-score in this figure.

the fig 4. These results can be summarized as follows: KS methods show a clear bias for larger signal with smaller classes, again highlighting the need for the normalization with specific class size [1, 3, 4] for these methods. Surprisingly also normal t-test shows strong bias that occurs among the very small class sizes, estimated to be the result of the uncertainty in the variance estimate for a small class size. This was corrected by adding a prior variance for regularization also to t-test. Regularizations with weights 1 and 3 showed a reasonable performance (see Material Methods for details), and these turn out to be most stable scores against the variations in the class size. Also iGA showed a trend with weaker signal for small class sizes (figure shows -log10 of best p-value). This is surprising as iGA is based on the p-value from hypergeometric distribution, a test often considered to be 'exact', and the publications using hypergeometric test have not discussed about the class size specific normalization [5]. Nevertheless the negative bias with smaller classes can be also useful as with smaller class the signal should be stronger in order to be reliable.

As a summary, the raw GSZ-score is unstable, but its improved regularized version is stable against the variations in the gene list subset and gene class size. Furthermore, to our surprise it shows even more

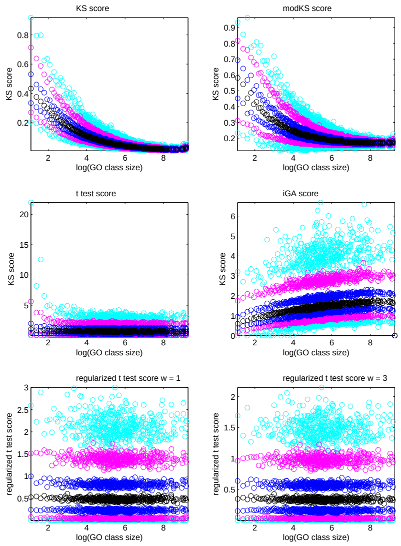

Figure 4: Stability of various other threshold free methods against the variations in the gene class size. Methods represented are as follows: KS test and modKS test, middle row: normal t-test and iGA, and bottom row regularized t-test w=1 and regularized t-test w=3. Results are obtained with the same 200 randomizations used in the fig 3. Also the same percentiles are represented with the same colour coding.

stable behaviour across different class sizes than the method based on hypergeometric distribution. This stability proposes that GSZ-score could be used in some applications even without any normalizations with randomized data. T-test also showed even better stability with similar regularization against the variations in the class size. Note that the variations across different class sizes can be normalized with row randomizations (discussed later), but the biases between different threshold positions are harder to normalize.

Stability of various methods with column randomizations

Earlier stability analysis concentrated on the stability under randomization of class labels. Still this randomization omits the correlations between the genes, which cause strong false positive signals with real data [3, 6, 7]. These can be monitored by looking the results from the randomized sample labels, where the correlations between the genes are conserved (column randomizations, see Materials and methods). This is demonstrated here with 200 randomizations for ALL (Acute Lymphatic Leukemia) dataset (see Materials and Methods). Distribution for each class is shown in the figure 5 for the most stable methods from earlier

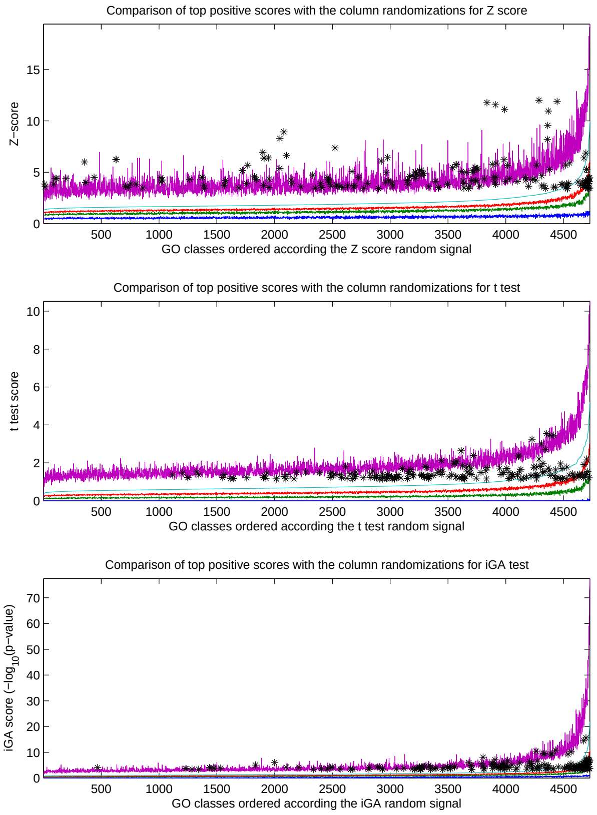

Figure 5: Stability of various score functions (Z-score = GSZ-score, T-test, iGA) in column randomizations. GO classes are shown in the increasing order of the random signal. Blue, green, red, cyan and magenta line show five percentiles (0, 25, 50 75, 100) for 200 randomizations. Black stars represent scores for the top 200 classes, for each method, from the positive data. Notice the strong bias in all the methods for classes at far right. Notice also the better separation with GSZ-score between the random and positive scores, when compared to other methods.

chapters. These figures show drastic biases in the results for various methods, especially for iGA.

Note that, although only few methods are represented here, the same bias can be expected to affect all GO based analysis methods [6]. This bias can be corrected by generating a pool of randomizations for each class. This pool can be used to normalize the results for each class, with the mean and STD of the randomized results (see Materials and methods). Also a relatively small number of randomizations can generate a stable normalization. This was demonstrated by splitting the column randomized data into two halves. First half is used to define the mean and STD estimates, and the second half is normalized using

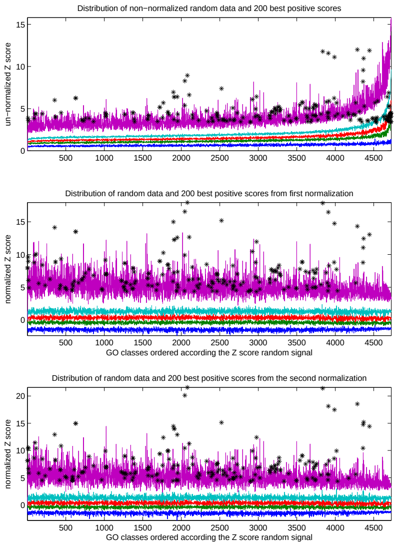

Figure 6: Normalization of GSZ-score column randomizations with mean and STD estimates from randomized data. Same percentiles (same as in fig. 5) are shown for last 100 randomizations in each plot. Black stars represent again top positive results, after normalizing similarly like the random data. Highest figure: No normalization; Middle figure: Normalization using first 100 randomizations; lowest figure: Normalization using first 50 randomizations.

these parameters, and then visualized with top scores from the un-randomized data to see how stable the normalization is. Results, in the fig. 6, show reasonable good stabilization. The only unwanted phenomenon seems to be the slight bias in the maximum values, potentially caused by too strong correcting effect. Therefore, we propose the normalization where one normalizes data separately with row and column randomizations, and selects the less significant (p-value or raw score) of two results. In later work we normalize also other methods in a similar fashion, using simply (moment based) mean and variance of the class specific randomizations.

Evaluation of different method normalizations

Normalizations with randomized data play crucial role in our method evaluation, as bad normalization could cause a bias and weak performance of the evaluated methods, especially when ranks of the normalized scores are used to evaluate the signal (see main text 'Evaluation of the methods with real dataset') and when the classes are pooled for p-values (see main text 'Comparison of empirical p-value

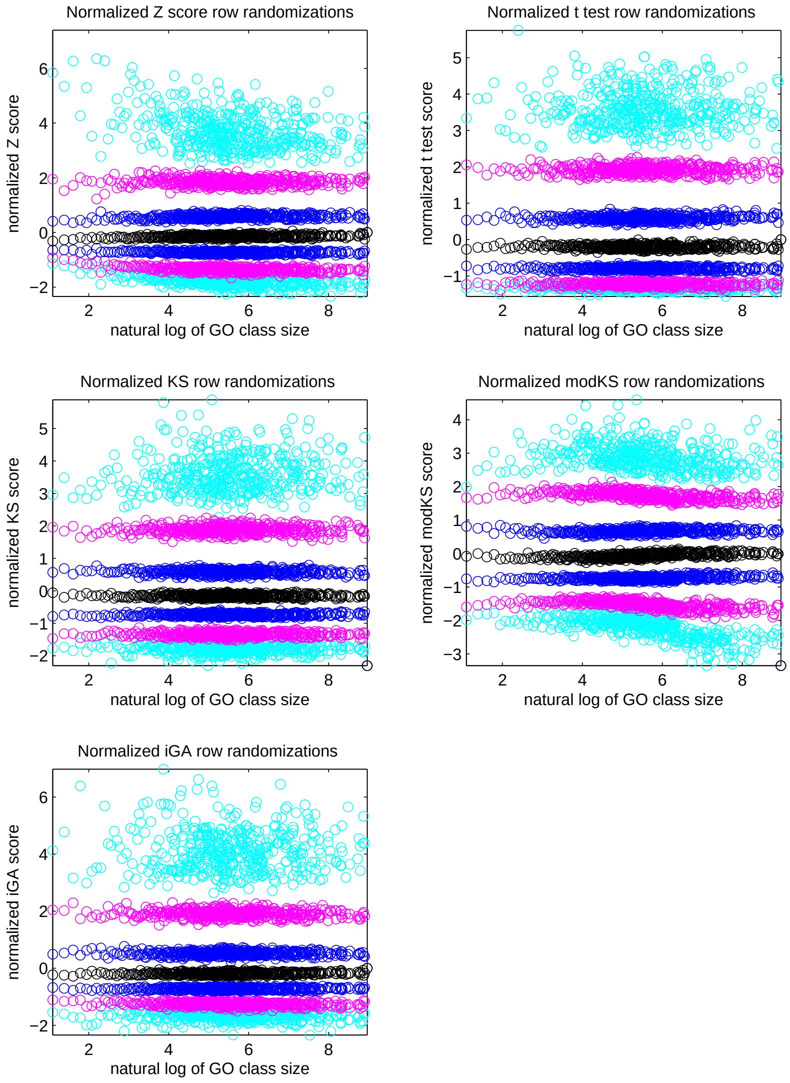

Figure 7: Stability of 200 row randomization results after the used GSZ-score normalization. Results are monitored against varying class size. Only a single randomization is shown for each class size with seven percentiles (0, 5, 35, 50, 75, 95, 100). Percentiles are coloured differently so that 0 and 100 are cyan, 5 and 95 are magenta, 25 and 75 are blue, and 50 is black. Figures show quite stable distributions.

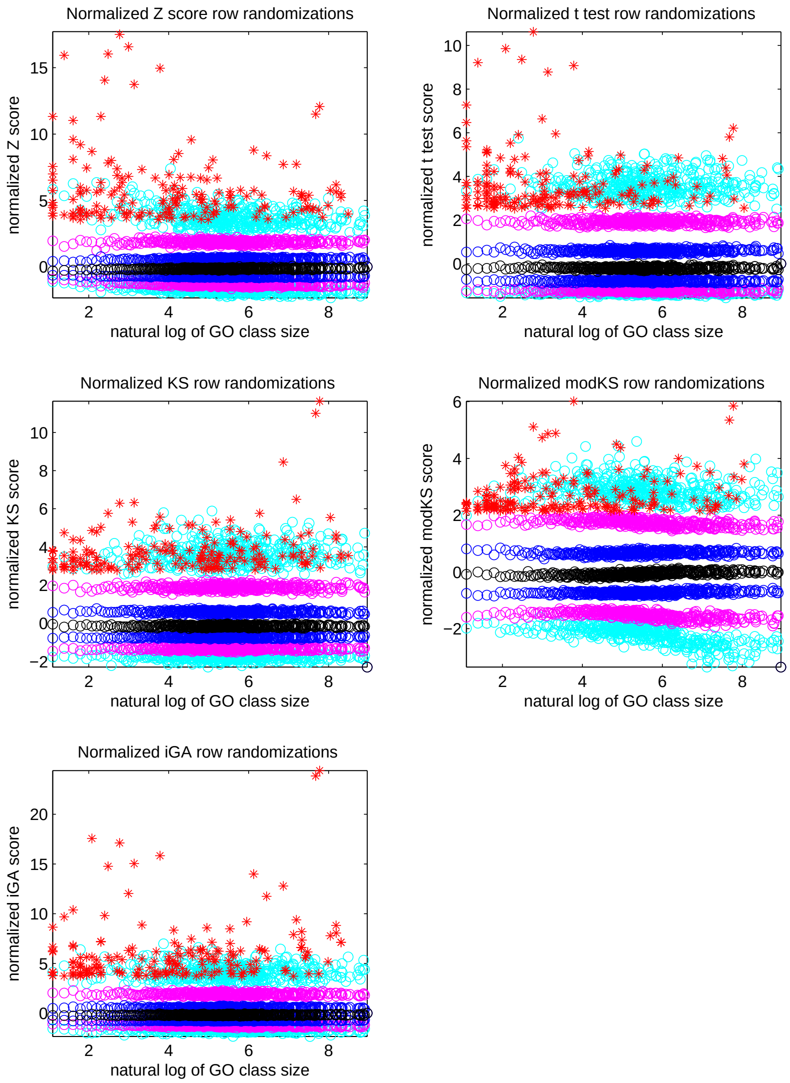

Figure 8: Representation of the fig. 7. Here the top 200 results, for each method from the original dataset, are represented as asterisks. This highlights the differences between the random data and top-scoring unrandomized results. Top results and the randomizations are normalized in a similar way

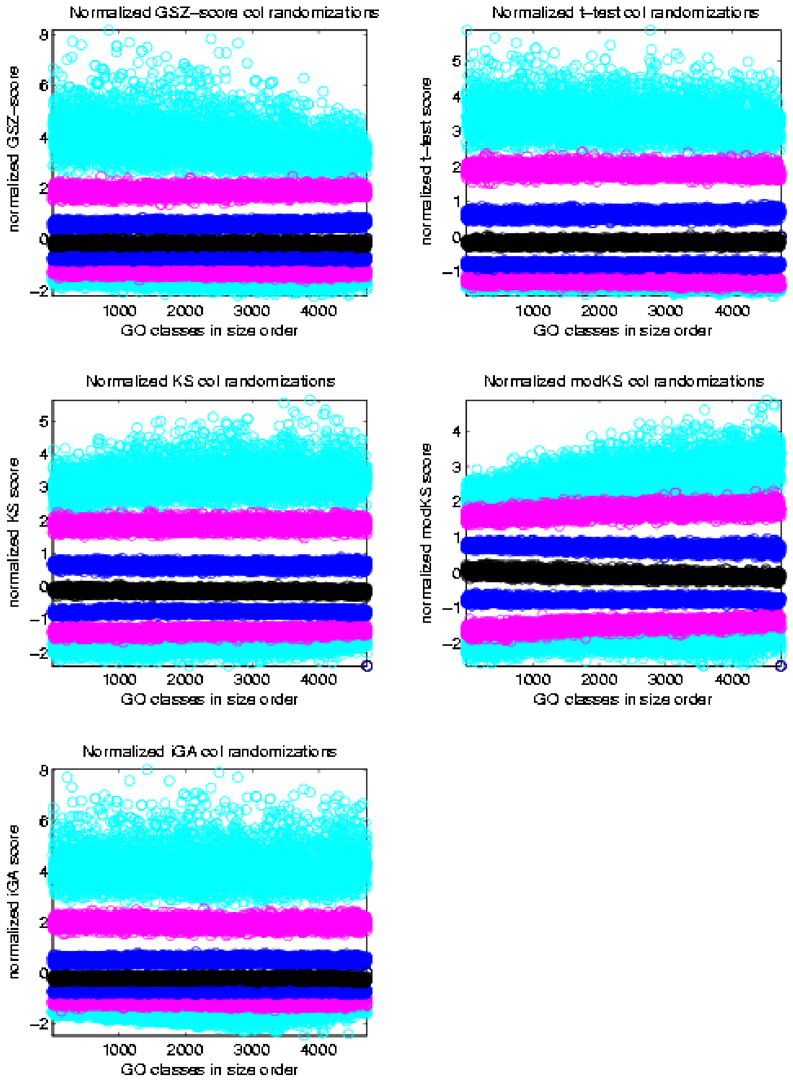

Figure 9: Stability of the column normalized results from different scoring functions in 200 column randomizations. GO classes are represented in size order starting with smaller classes, with each class shown as a separate column. Same percentiles as in fig. 1 are shown from each randomization. Figures show stable distributions.

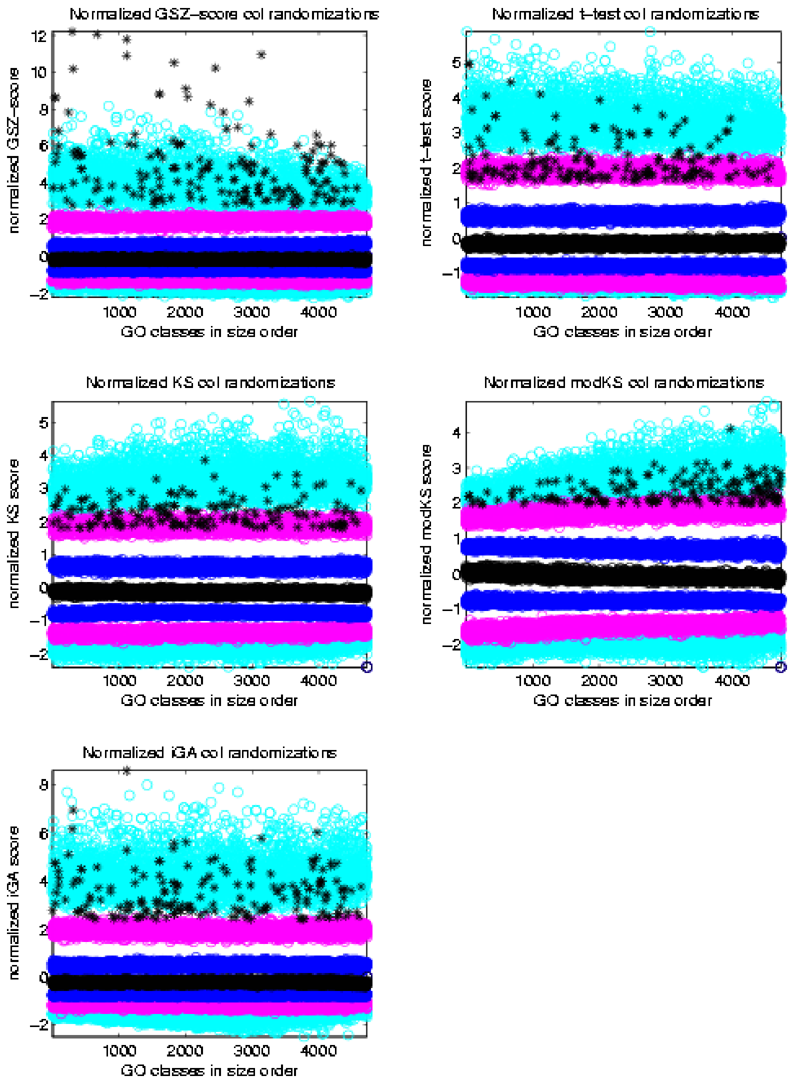

Figure 10: Representation of the fig. 9. Again the top 200 results, for each method from the original dataset, are represented as asterisks. Again the aim is to visualize the differences between randomized and top-scoring un-randomized results. Top results and the randomizations are normalized in a similar way

signals'). Therefore it is central to evaluate normalization to see whether the GO classes represent a stable distribution with randomized data. This was done by visualizing different percentiles for functional classes as the class sizes vary. Results are shown in figures 7-10. Note, that the t test scores in these comparisons are the normalized version of the absolute t test.

Figure 7 shows the obtained stability in row randomizations. It shows a distribution of 200 randomizations for each GO class size, by representing seven percentiles (0, 5, 25, 50, 75, 95 100), with the interesting ones being the top percentiles. Figure shows a single set of randomizations for each class size. Most of the percentiles show stability across the changing class sizes. Furthermore, t test and iGA show here the best stability across different percentiles. iGA distribution is now more stable, than without the random data normalization. There are some less stable percentile profiles: The maximum scores of the GSZ-score show some bias, with slightly larger average of maximum signals for smaller classes. The maximum of normalized KS score shows weaker signal for smaller classes. However, the maximum values can be expected to be less stable than the other percentiles. Furthermore, when we add the top 200 classes from the non-randomized data to the figure, we see a clear separation of random and positive data especially with GSZ-score, iGA and t test. Also, the top scoring classes represent varying class sizes, proposing that the bias seen with the GSZ-score is insignificant. The most unstable behaviour is seen in the distribution of the normalized modKS score. Also this distribution is somewhat stable after random data normalization, but still there are some small fluctuations in many of its percentiles.

Similar visualizations were also done to column randomizations (fig 9-10). Here we have a separate randomization for every GO class. We represent all the GO classes in the size order, starting with the smallest classes. Note here that we have many classes of same size. The obtained distribution looks quite stable. Now the only slight deviation in the stability seems to be the distribution of the maximum with modKS score. Here it again shows weaker signal for the smaller classes. Interestingly GSZ-score has many top positive scores with clear separation to the randomized results.

We also further visualize the differences between the two random data normalizations. These are represented by showing the scatter in figure 11 for each method with normalization, based on column randomizations, on X axis and normalization, based on row randomizations, on Y axis. Here one sees that most of the classes with most of the methods get more conservative results with column randomizations, which corresponds with earlier publications [3, 6, 8]. Still there are some observations especially in

GSZ-score, modKS and in iGA that get larger result in column randomization pointing to usefulness of monitoring both of them [7].

What is also interesting is to see how the empirical p-values, obtained from both randomizations behave. P-values were calculated by pooling all the randomizations from every class. These results are visualized similarly to figure 11 in figure 12. Figure represents $\log_{10}$(p-value), and undefined cases (where empirical p-value=0) are ad-hoc corrected by replacing 0 in class count with 0.5. Now value log(p-value) 6.28 corresponds to case that none of the classes produced as strong signal in any of the randomizations. Again many methods show this for the row randomizations, but only two methods, iGA and GSZ-score, show classes with such results for both the row and the column randomizations. Furthermore iGA had only single class showing this whereas in GSZ-score results show 15 such classes. The figures 11 and 12 highlight that the results would benefit from selecting the less significant (test score or p-value) from two normalizations.

Detailed analysis of differences in class p-values

In order to further evaluate the differences between the class scoring functions we focused on the pairwise differences in the p-values for each GO class between GSZ-score and each of the other functions. This allows the analysis of actual GO classes that represent the strongest separation between the functions. First we need to combine the different p-values reported for each method. We use the p-value combination described later in the materials and methods. Combined p-values were used to look for differences between GSZ and each of the other methods by simply taking the difference in $\log(\text{p-value})_{combined}$ between compared methods. We monitored classes with 20 strongest differences where GSZ-score outperforms the compared method and 20 strongest differences where the compared method outperforms GSZ-score (see supplementary tables 3 and 4, additional data file 9 and 10). KS was omitted from the detailed comparison since it showed weakest performance in all the earlier comparisons. A summary of the results is represented here in the tables 1 - 6, which show 10 top classes in favour and also against GSZ-score. Tables also show the log(p-value) for GSZ-score and for competing method. Also the mean of differential gene expression t-test scores (differential expression test scores) between the two sample types across the GO class members (shortened here as mean expression scores) and mean of absolute values of the same gene expression t-test scores (shortened as mean of absolute expression scores) are represented. The first average measures consistent up- or down-regulation of the GO class. The latter average measures simultaneous up and down-regulation.

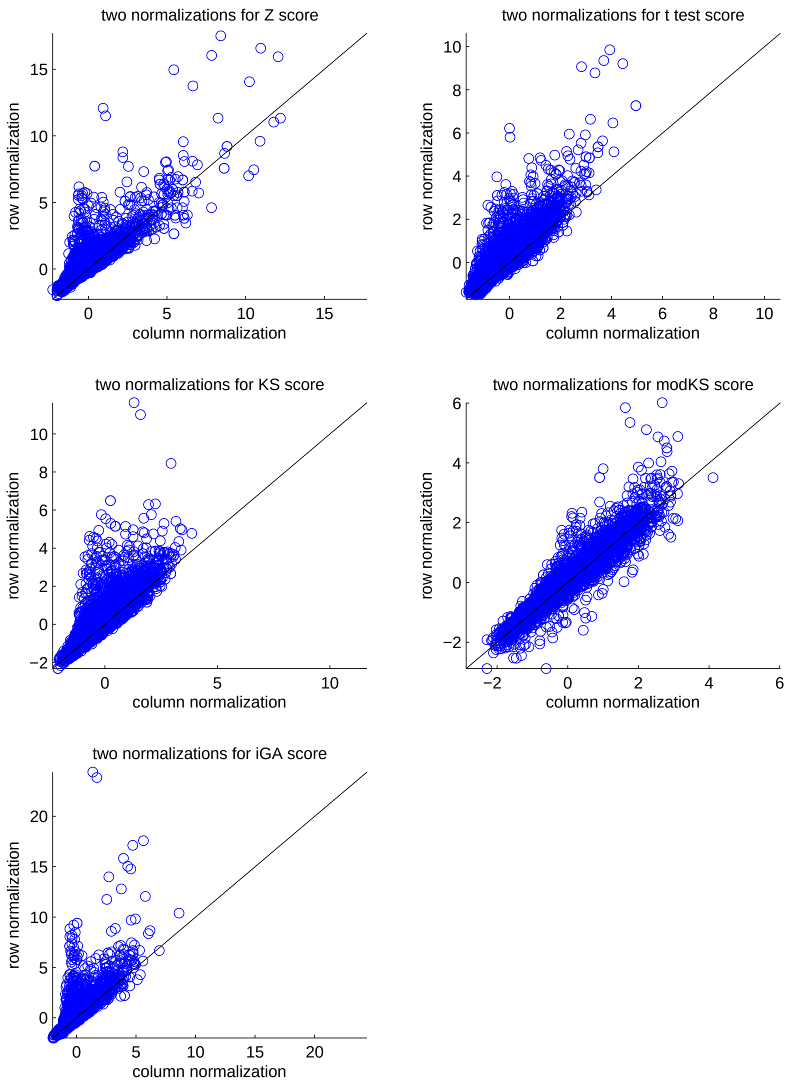

Figure 11: Comparison of each methods results when using two random data based normalizations. Notice that the column randomizations are in most cases more conservative. Black line shows $x = y$ line.

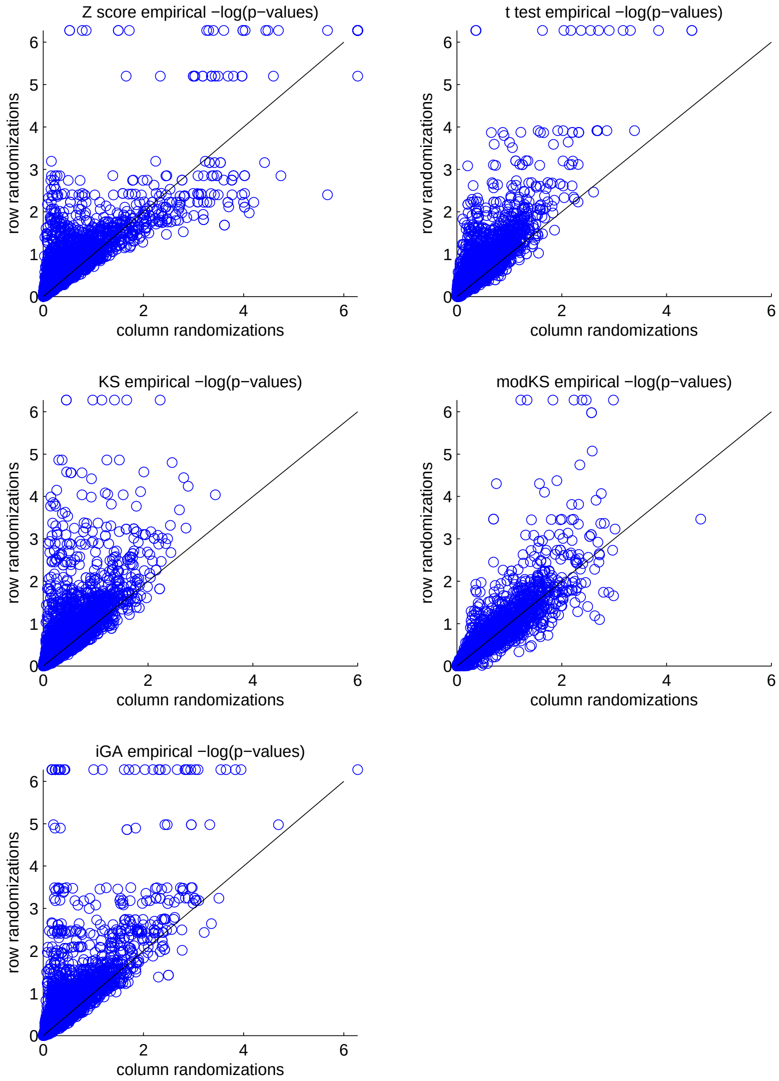

Figure 12: Comparison of each methods empirical p-values when using two random data based normalizations. Notice that the again column randomizations are in most cases more conservative. Still there are some cases in GSZ-score and modKS where row randomization is more conservative. Black line shows $x = y$ line.

GSZ-score vs. t-test

The strongest signals from the comparison with t-test from ALL data are shown in table 1. Table shows classes with dramatic differences (class data log-p-value differences 4.7, 3.8, 3.5) favouring GSZ-score, with 3100 - 50000 fold difference in p-values. Differences highlight that t-test is unable to detect simultaneous up and down-regulation, observed in the classes like 'immunological synapse', 'T cell receptor complex', 'positive thymic T cell selection'. Surprisingly, also a small down-regulated class with a scattered expression 'positive regulation of antigen receptor-mediated signalling pathway' obtains log(p-value) difference of 3.8, favouring GSZ-score. Biological analysis reveals that these classes are mostly associated with T cell selection and immune response development linking them strongly to the research setup (B cells vs. T cells). These classes point that it would be naïve to monitor only GO classes with clear up or down regulation, and point biological trends (mainly 'thymic T cell selection' and 'immunological synapse' related classes) that would be totally lost when neglecting heterogeneous behaviour.

The opposite classes, showing strongest preference for t-test, represent only extremely small log differences (less than 0.65, a mere 4.5 fold difference). Therefore we only represent here the top four classes. Furthermore, the reported classes are more biologically heterogeneous ('AP-type membrane coat adaptor complex', 'Rap GTPase activator activity' etc.) with less clear link to experiment setup. These classes were also low in the ranked t-test result list, which suggest that these differences might be normal random variation between the two results. These results propose that GSZ-score is able to detect the signals that t-test finds here but not the other way around. t-test could be also modified to monitor the absolute values, but this would of course require the usage of two separate tests, one for signed and one for absolute values.

Similar analysis was also done with p53 dataset (table 2). There differences favouring GSZ were smaller, but still larger than differences favouring t-test. GSZ also performed better on positive classes, like apoptotic mitochondrial changes, caspase activation via cytochrome c etc. Furthermore also the other classes, not considered as positive, can be linked to processes related to apoptotic signal pathway, like mitochondrial membrane. The surprisingly weak performance of t-test proposes that it is not the best candidate among the scoring functions that are based on summation of differential expression scores of GO class. Indeed, we show better performance by Signal Pathway [9] in our program package comparison. Signal Pathway uses simple sum of differential expression scores and omits the variance of expression scores. However, Signal Pathway is also unable to report classes representing simultaneous up and

down-regulation.

GSZ-score vs. iGA

Results from comparison between GSZ-score and iGA on ALL dataset are represented in table 3. This comparison shows also large differences, preferring GSZ-score. Three strongest differences come from classes that show consistent down-regulation (mean expression values: -15.5, -18.3 and -8.7). These should be easy to detect, but unexpectedly iGA reports significantly weaker p-values for these classes. Furthermore, the differences are 3.5, 3.5, and 2.9 (in class data p-values), which means 3100 - 790 fold difference in p-values. Biological analysis reveals repetitive occurrences of classes related to MHC class II receptor, to antigen processing and to T cell receptor complex.

Again the top opposite differences are smaller (largest difference 1.0) with more scattered functional descriptions like receptor tyrosine kinase binding, vitamin metabolic process and dendrite development. These are also less clearly linked to experiment setup than the classes favouring GSZ-score.

Same comparison was also done using p53 dataset, shown in table 4. Also here GSZ gives clear better signals on three positive classes: Apoptotic mitochondrial changes, release of cytochrome c from mitochondria and induction of apoptosis by extracellular signals. However there is one exception: We notice ARF protein signalling classes. These protein classes represent a border case in p53 dataset, and thus we mark them with underline. They were not reported as being regulated by p53, but nevertheless they are central to apoptotic signalling. Although the performance difference between GSZ and iGA on this dataset is less clear cut, GSZ seems to outperform iGA with biologically relevant classes.

The differences between these two methods are interesting, as in GO class ordering they gave quite similar results (see table 5 in main text). Detailed analysis shows that GSZ-score surpassed especially with the classes showing strong regulation. GSZ-score uses the actual signal intensities, whereas iGA takes as the input only the order of the genes and omits the actual signal levels. This causes differences between these methods in column randomization when the original dataset represents strong regulatory signal.

GSZ-score vs. modKS

Results from comparison of log(p-values) between GSZ-score and modKS from ALL dataset are shown in table 5. Comparison shows large differences, with largest difference favouring GSZ-score being 5.4 (250000

Table 1: Classes where GSZ-score and t-test showed largest difference in ALL dataset

			GO class info		GSZ log(p-values)		t-test log(p-values)	
Difference	mean expr	mean of abs(expr)	Class size	Class name	pooled data	class data	pooled data	class data
3.39	-4.13	10.56	20	**CC::0001772:: immunological synapse**	5.83	3.30	1.15	1.19
3.15	-1.13	15.27	6	**BP::0045059::positive thymic T cell selection**	3.43	3.30	0.22	0.21
3.15	-1.13	15.27	6	**BP::0043368::positive T cell selection**	3.43	3.30	0.22	0.21
3.10	1.11	5.51	148	**BP::0046649:: lymphocyte activation**	3.03	3.30	0.07	0.07
2.98	0.55	7.61	16	**BP::0002429::immune response-activating . . . pathway**	3.10	3	0.077	0.07
2.98	0.55	7.61	16	**BP::0002768::immune response-regulating . . . pathway**	3.10	3	0.08	0.08
2.96	0.96	5.41	165	**BP::0045321::leukocyte activation**	2.98	3	0.03	0.02
2.91	-2.51	11.00	10	**BP::0045061::thymic T cell selection**	3.70	3.30	0.59	0.59
2.88	0.62	8.09	15	**BP::0050851::antigen receptor-. . . pathway**	2.88	3	0.06	0.06
2.85	0.85	5.90	47	MF::0060090::molecular adaptor activity	2.50	3.30	0.06	0.05
0.65	-6.76	6.76	5	BP::0042384::cilium biogenesis	2.17	1.96	2.42	3
0.64	3.43	4.03	16	CC::0030119::AP-type membrane . . .	0.85	0.86	1.50	1.50
0.61	-2.97	2.97	9	BP::0021537::telencephalon development	1.40	1.47	1.93	2.15
0.59	-1.49	3.56	25	BP::0016358::dendrite development	1.21	1.21	1.78	1.82

Table highlights classes where the p-value from GSZ-score and t-test showed the clearest difference on ALL dataset. Upper part shows classes favouring GSZ and lower part classes favouring t-test. Both sets show the ranked list of classes, starting with the class showing largest difference. The first column represents the difference in the log10(p-value) between the compared methods. The second column shows average differential expression of GO class and the third column the same average calculated from absolute values of differential expression. Third and fourth columns represent the GO class size and GO class name and ID. Abbreviated GO class names have been marked with three dots. Positive GO classes are represented with bold font. The sixth and seventh class represent two empirical log10(p-values) obtained with GSZ. The last two columns represent the same log10(p-values) for t-test. All these values are minimums of log(p-values) from row and column randomization. Supplementary tables, however, represent all four log(p-values) for both methods. As the differences favouring t-test are very small, we only represent four classes from the whole list. Note that classes where GSZ-score surpassed t-test have large mean absolute values and small normal mean values. These values suggest simultaneous up and down-regulation.

Table 2: Classes where GSZ-score and t-test showed largest difference in p53 data

			GO class info		GSZ log(p-values)		t-test log(p-values)	
Difference	mean expr	mean of abs(expr)	Class size	Class name	pooled data	class data	pooled data	class data
3.09	-0.33	1.57	13	**BP::0008637::apoptotic mitochondrial changes**	3.43	3	0.13	0.12
2.65	-1.12	2.70	3	BP::0051205::protein insertion into membrane	3.28	2.70	0.35	0.33
2.36	-1.08	1.83	4	**BP::0008635::caspase activation via cytochrome c**	3.03	2.70	0.50	0.50
2.27	-1.64	2.44	6	BP::0051668::localization within membrane	4.13	3.30	1.40	1.48
1.85	-1.96	2.14	6	**BP::0001836::release of cytochrome c from mitochondria**	5.11	3.30	2.41	2.30
1.84	-0.44	1.57	7	BP::0007006::mitochondrial membrane ...	2.06	1.96	0.17	0.167
1.79	-0.47	2.01	4	BP::0045736::negative regulation of cyclin-dependent ...	1.99	1.85	0.13	0.128
1.77	-0.14	1.17	22	CC::0005741::mitochondrial outer membrane	1.86	1.80	0.06	0.06
1.75	-0.72	1.20	21	**BP::0008629::induction of apoptosis by intracellular signals**	2.80	3	1.17	1.14
1.72	-0.70	1.42	7	BP::0008634::negative regulation of survival ...	2.70	2.05	0.45	0.46
0.89	1.11	1.11	6	MF::0016018::cyclosporin A binding	1.58	1.60	2.25	2.70
0.80	1.01	1.18	6	BP::0043542::endothelial cell migration	1.19	1.24	1.99	2.05
0.79	-1.70	1.70	5	BP::0018298::protein-chromophore linkage	1.85	1.80	2.70	2.52
0.79	-1.84	1.84	5	BP::0006572::tyrosine catabolic process	1.86	1.70	2.73	2.40
0.70	-1.31	1.35	8	BP::0045453::bone resorption	1.74	1.70	2.43	2.40
0.68	-1.54	1.54	5	MF::0009881::photoreceptor activity	1.75	1.72	2.52	2.30
0.66	-0.87	1.06	14	BP::0007416:: synaptogenesis	0.95	0.93	1.61	1.59

Table highlights classes where the p-value from GSZ-score and t-test showed the clearest difference on p53 dataset. Upper parts shows classes favouring GSZ and lower part classes favouring t-test. Both parts show classes with largest differences. Columns are similar to the earlier table. As the differences favouring t-test are very small, we only represent seven classes from the whole list. Note that classes where GSZ-score surpassed t-test have large mean absolute values and small normal mean values.

Table 3: Classes where GSZ-score and iGA showed largest difference in ALL dataset

			GO class info		GSZ log(p-values)		iGA log(p-values)	
Difference	mean expr	mean of abs(expr)	Class size	Class name	pooled data	class data	pooled data	class data
2.29	-18.32	18.32	4	**CC::0042105::alpha-beta T cell receptor complex**	5.83	3.30	2.33	2.22
1.91	-8.70	10.65	11	**CC::0042101::T cell receptor complex**	5.63	3.30	2.72	2.40
1.90	-15.49	15.49	3	**BP::0050857::positive regulation of antigen receptor . . . pathway**	6.67	3.30	3.17	3
1.88	-4.13	10.56	20	**CC::0001772:: immunological synapse**	5.83	3.30	2.98	2.40
1.67	0.87	5.89	47	MF::0060090::molecular adaptor activity	2.50	3.30	1.24	1.22
1.46	10.77	10.82	16	**BP::0002504::antigen processing . . . via MHC class II**	4.92	3.30	2.78	2.52
1.36	-4.16	5.21	13	BP::0042094::interleukin-2 biosynthetic process	2.47	2.70	1.22	1.22
1.36	-4.16	5.21	13	BP::0032623::interleukin-2 production	2.47	2.70	1.22	1.22
1.35	-4.91	5.62	11	BP::0045076::regulation of interleukin-2 . . .	2.55	2.40	1.12	1.11
1.34	9.44	9.57	7	**BP::0002478::antigen processing . . .**	3.33	3	1.83	1.82
1.09	-2.97	2.97	9	BP::0021537::telencephalon development	1.40	1.47	2.35	2.70
0.96	-0.77	3.61	38	BP::0006766::vitamin metabolic process	1.16	1.16	2.20	2.05
0.95	-1.49	3.56	25	BP::0016358::dendrite development	1.21	1.21	2.17	2.15
0.92	6.86	6.86	3	MF::0030971::receptor tyrosine kinase binding	1.36	1.35	2.40	2.15
0.91	1.33	4.52	12	MF::0015175::neutral amino acid . . .	0.36	0.36	1.28	1.27
0.91	-0.08	3.50	48	BP::0006112::energy reserve metabolic process	0.90	0.80	1.74	1.77
0.90	2.19	4.08	13	BP::0050885::neuromuscular process . . .	0.59	0.58	1.48	1.49
0.90	6.06	6.06	5	BP::0046479:: glycosphingolipid catabolic process	1.51	1.46	2.47	2.30

Table highlights classes where the p-value from GSZ-score and iGA showed the clearest difference on ALL dataset. Upper part shows classes favouring GSZ and lower part classes favouring iGA. Both parts show classes with largest differences. Columns are similar to the earlier tables. We show less cases favouring iGA due the space limitations. Note that classes where GSZ-score surpassed iGA show clearer regulation.

Table 4: Classes where GSZ-score and iGA showed largest difference in p53 dataset

			GO class info		GSZ log(p-values)		iGA log(p-values)	
Difference	mean expr	mean of abs(expr)	Class size	Class name	pooled data	class data	pooled data	class data
1.23	-1.65	2.44	6	BP::0051668::localization within membrane	4.13	3.30	2.45	2.52
1.17	-0.33	1.57	13	**BP::0008637::apoptotic mitochondrial changes**	3.43	3	2.04	2.05
0.89	-0.47	0.78	38	MF::0015662::ATPase activity ...	1.57	1.57	0.68	0.67
0.88	-1.98	1.98	5	BP::0030890::positive regulation of B cell ...	2.86	2.70	1.92	1.89
0.88	-1.96	2.14	6	**BP::0001836::release of cytochrome c from mitochondria**	5.11	3.30	3.36	3.30
0.88	-0.50	1.21	30	**BP::0008624::induction of apoptosis by extracellular signals**	1.76	1.89	0.95	0.95
0.84	-0.57	0.75	12	MF::0015085::calcium ion transmembrane ...	1.34	1.32	0.49	0.49
0.76	1.44	1.82	3	BP::0046463::acylglycerol biosynthetic process	2.56	2.70	1.79	1.96
0.76	1.44	1.82	3	BP::0046460::neutral lipid biosynthetic process	2.56	2.70	1.79	1.96
0.70	0.23	1.06	30	MF::0019888::protein phosphatase ...	2.09	2.30	1.51	1.47
1.22	1.11	1.11	6	MF::0016018::cyclosporin A binding	1.58	1.60	2.62	3
1.13	-0.45	1.02	69	BP::0006725::aromatic compound metabolic process	1.03	1.02	2.15	2.15
1.11	0.34	0.93	16	BP::0032259::methylation	0.93	0.90	1.90	2.15
1.11	0.34	0.93	16	BP::0043414::biopolymer methylation	0.93	0.90	1.90	2.15
1.03	-0.57	1.05	131	BP::0050953::sensory perception ...	1.85	2.10	2.71	3.30
1.03	-0.57	1.05	131	BP::0007601::visual perception	1.85	2.10	2.71	3.30
1.01	0.35	0.77	15	BP::0043407::negative regulation of MAP kinase activity	0.90	0.94	1.87	2
1.01	-0.68	0.68	12	MF::0005086::ARF guanyl-nucleotide ...	1.73	1.72	2.78	2.70
1.01	-0.68	0.68	12	BP::0032012::regulation of ARF protein signal ...	1.73	1.72	2.78	2.70
1.01	-0.68	0.68	12	BP::0032011::ARF protein signal transduction	1.73	1.72	2.78	2.70

Table highlights classes where the p-value from GSZ-score and iGA showed the clearest difference on p53 dataset. Upper part shows classes favouring GSZ and lower part classes favouring iGA. Both parts show the classes with largest differences. Columns are similar to the earlier tables. GSZ shows better performance on positive classes. The only exception is ARF protein signal transduction classes, which show better signal with iGA.

fold difference in p-values!) This is natural as these two methods had a significant difference already in the top log-p-values (see figures 4 and 5 from manuscript). Top classes are related to positive regulation of antigen receptor, alpha-beta T cell receptor complex and immunological synapse.

Also the differences in the classes favoured by modKS are larger than in previous comparisons (2.1, 1.7, 1.7. . .). Still these are clearly smaller than the differences in the earlier classes that favoured GSZ-score. These classes also seem less relevant to the research setup (glycolipid metabolic process, transcription from RNA polymerase II promoter, dynein complex etc.). modKS performed also better with some very vague large classes like: Regulation of cellular process, positive regulation of metabolic process, regulation of biological process. These classes seem quite uninformative from the biological perspective. Furthermore, when the classes where modKS was better are compared with classes where GSZ-score was better, we notice that classes like glycolipid metabolic process and regulation of cellular processes obtained better p-value from modKS than most of the T-cell receptor related classes or MHC class II related classes. This seems to be in contrast with the known biology of test setup. It also contradicts the stronger regulation of these classes, represented by the two mean values.

We also show comparison GSZ score and modKS with p53 dataset in table 6. Although the differences in this dataset were smaller GSZ clearly outperforms modKS on two positive classes: apoptotic mitochondrial changes and caspase activation via cytochrome c. Many other classes, where GSZ was better, can be indirectly linked to apoptotic processes, like protein insertion into membrane and mitochondrial membrane organization and biogenesis. The opposite classes, where modKS outperformed GSZ seem to be arbitrary collection of GO classes.

The drastic differences favouring GSZ-score are likely caused by the more stable behaviour of GSZ-score across different threshold positions, shown in figures 1 and 2, earlier in this document. These figures also showed clearer differences for GSZ-score between real data and randomized data than for modKS. Analysis of different threshold positions with modKS represents a bias in null distribution, where the threshold positions close to the ends of the gene lists are preferred. This was the original goal of the modification created to the KS test [3], as the KS is known to prefer the threshold positions at the centre region of the gene list (see figure 2). However, the obtained null distribution, represented in the figure 2, proposes that the effect has been too strong and has started to favour tails of the gene list. Also a detailed analysis of classes, where modKS showed the best performance revealed that these classes have one or few strong

expression outliers with majority of the class having mild regulation, which suggests that modKS might be more biased to favour outlier signal. This is highlighted by the smaller mean expression value and smaller mean of absolute expression values, reported for these classes, than the mean values reported for classes favouring GSZ-score. Note that optimal test should definitely give good results, when classes show large deviation from null in either of two mean values. These results propose that modKS is quite suboptimal scoring function for gene set analysis.

GSZ-score vs. KS

Earlier comparisons propose very weak performance for standard KS. Therefore, we omit the detailed analysis here and represent only few details. Comparison on ALL dataset is represented in supplementary table 3. Here KS does not report signal at all to the top scoring positive classes (log(p-values) 1), whereas GSZ reports log(p-values) around 3 - 7. Resulting differences in log(p-values) for the positive classes obtained with class data range from 2.5 to 5.6, highlighting GSZ as clearly better method. Only exception is the very weakly regulated class of interleukin-12 production.

Similar results were also obtained p53 dataset. There KS does not report any signal to the p53 related apoptosis classes. However we see again that GSZ is outperformed ARF protein signalling classes. These results propose that KS should not clearly used as the only test to analyze regulation of gene classes. However, KS or iGA could be used in combination with GSZ to monitor classes with weak or very weak regulation. Nevertheless, KS had also weak performance in cross validation test, where one task was to predict its own results from other half of data. This suggests that these weak signal classes might be unstable.

As a summary the differences favouring GSZ-score were bigger and they were regularly related to GO classes that were strongly linked to research setup. The differences favouring other methods were clearly smaller and came from the very various GO classes. These seem to be more random fluctuation observed in the results, rather than consistent signal. Also the classes selected by GSZ-score showed larger differences from null in the mean of signed expression values and/or in the mean of absolute expression values. These are classes that an optimal test should definitely select as they show a quite clear deviation from null.

Table 5: Classes where GSZ-score and modKS showed largest difference in ALL dataset

		GO class info			GSZ log(p-val)		modKS log(p-val)	
Difference	mean expr	mean of abs(expr)	Class size	Class name	pooled data	class data	pooled data	class data
3.51	-15.49	15.49	3	**BP::0050857::positive regulation of antigen . . . pathway**	6.67	3.30	1.27	1.68
2.98	-1.13	15.27	6	**BP::0045059::positive thymic T cell selection**	3.42	3.30	0.39	0.38
2.98	-1.13	15.27	6	**BP::0043368::positive T cell selection**	3.43	3.30	0.39	0.38
2.82	-18.32	18.32	4	**CC::0042105::alpha-beta T cell receptor complex**	5.83	3.30	1.45	2.05
2.80	-4.13	10.56	20	**CC::0001772::immunological synapse**	5.83	3.30	1.83	1.70
2.64	-2.51	11.00	10	**BP::0045061::thymic T cell selection**	3.70	3.30	0.84	0.87
2.54	0.55	7.61	16	**BP::0002429::immune response-activating . . . pathway**	3.10	3	0.51	0.52
2.54	0.55	7.61	16	**BP::0002768::immune response-regulating . . . pathway**	3.10	3	0.51	0.52
2.46	0.62	8.09	15	**BP::0050851::antigen receptor-mediated signaling pathway**	2.88	3	0.47	0.48
2.45	0.87	5.89	47	MF::0060090::molecular adaptor activity	2.50	3.30	0.45	0.45
1.88	2.73	3.81	22	BP::0006664::glycolipid metabolic process	0.87	0.86	2.97	2.52
1.84	3.72	3.82	10	BP::0031960::response to corticosteroid stimulus	1.10	1.07	2.55	3.30
1.71	2.67	2.75	6	BP::0040015::negative regulation of multicellular organism growth	0.46	0.49	1.98	2.40
1.59	1.45	3.49	328	BP::0031325::positive regulation of cellular metabolic process	0.40	0.39	2.09	1.89
1.57	1.42	3.48	334	BP::0009893::positive regulation of metabolic process	0.37	0.37	2.03	1.85
1.57	1.42	3.54	511	BP::0006366::transcription from RNA polymerase II promoter	0.40	0.41	2.06	1.89
1.56	1.18	3.59	2421	BP::0050794::regulation of cellular process	0.42	0.44	2.17	1.82
1.53	2.50	3.42	15	CC::0030286::dynein complex	0.81	0.78	2.50	2.15
1.48	1.16	3.61	2565	BP::0050789::regulation of biological process	0.52	0.53	2.15	1.85
1.47	2.31	2.41	4	MF::0015368::calcium cation antiporter activity	0.07	0.08	1.49	1.60

Table highlights classes where the p-value from GSZ-score and modKS showed the clearest difference on ALL dataset. Upper half shows classes favouring GSZ and lower half classes favouring iGA. Both halves show the classes with largest differences. Columns are similar to the earlier tables. Classes preferring GSZ show clear stronger regulation, with clear positive biological link to analysis setup. Opposite classes are more or less random classes representing weak average signals.

Table 6: Classes where GSZ-score and modKS showed largest difference in p53 dataset

			GO class info		GSZ log(p-val)		modKS log(p-val)	
Difference	mean expr	mean of abs(expr)	Class size	Class name	pooled data	class data	pooled data	class data
2.35	-1.12	2.70	3	BP::0051205::protein insertion into membrane	3.28	2.70	0.65	0.63
1.85	-1.65	2.44	6	BP::0051668::localization within membrane	4.13	3.30	1.82	1.92
1.82	-0.33	1.57	13	**BP::0008637::apoptotic mitochondrial changes**	3.43	3	1.42	1.36
1.67	-1.98	1.98	5	BP::0030890::positive regulation of B cell proliferation	2.86	2.70	1.10	1.11
1.61	-0.47	2.02	4	BP::0045736::negative regulation of cyclin-dependent protein kinase activity	1.99	1.85	0.33	0.30
1.55	0.52	1.89	4	BP::0001837::epithelial to mesenchymal transition	2.06	1.85	0.42	0.39
1.53	0.79	1.31	18	CC::0031903::microbody membrane	3.19	3.30	1.84	1.59
1.53	0.79	1.31	18	CC::0005778::peroxisomal membrane	3.19	3.30	1.84	1.59
1.52	-1.08	1.84	4	**BP::0008635::caspase activation via cytochrome c**	3.03	2.70	1.28	1.42
1.47	-0.44	1.57	7	BP::0007006::mitochondrial membrane organization and biogenesis	2.06	1.96	0.54	0.54
1.94	-0.68	0.79	5	BP::0035136::forelimb morphogenesis	0.35	0.36	2.20	2.40
1.78	-0.64	0.66	3	MF::0004767::sphingomyelin phosphodiesterase activity	0.122	0.13	1.58	2.22
1.63	0.50	0.56	5	CC::0005675::holo TFIIH complex	0.53	0.56	2.14	2.22
1.60	-0.54	0.75	8	BP::0001976::fast regulation of arterial pressure	0.17	0.17	1.84	1.70
1.60	-0.53	0.75	8	BP::0001990::regulation of blood pressure by hormones	0.17	0.17	1.84	1.70
1.60	-0.54	0.75	8	BP::0050886::endocrine process	0.17	0.17	1.84	1.70
1.48	-0.39	0.82	32	MF::0008235::metalloexopeptidase activity	0.37	0.36	1.94	1.74
1.30	-0.37	0.87	80	MF::0042803::protein homodimerization activity	0.39	0.38	1.78	1.60
1.23	-0.45	1.02	69	BP::0006725::aromatic compound metabolic process	1.03	1.02	2.45	2.05
1.15	-0.88	1.10	8	MF::0004935::adrenoceptor activity	0.95	0.94	1.97	2.22

Table highlights classes where the p-value from GSZ-score and modKS showed the clearest difference on p53 dataset. Upper half shows classes favouring GSZ and lower half classes favouring iGA. Both halves show the classes with largest differences. Columns are similar to the earlier tables. Classes preferring GSZ show slightly stronger regulation, with few positive classes. Opposite classes are again more or less random classes representing weak average signals.

Materials and Methods

Other evaluated class scoring functions

There are many class scoring functions available for the threshold-free gene set analysis, and testing them all is outside the scope of this manuscript. Instead, we select few functions that represent very different approaches for the gene set analysis. In addition, we represent improvements on two earlier methods, iGA and to mean testing.

Probably the simplest idea for the threshold free gene set analysis is the mean of the differential expression scores for the gene class members $\sum(X_{pos})/N_{pos}$ [9]. Here X_{pos} is the expression score for member of gene class and N_{pos} is the size of gene class. This score can be compared with the same score, calculated for non-members, resulting into a difference between the two scores $\sum X_{pos}/N_{pos} - \sum X_{neg}/N_{neg}$. Here we decided to modify this further to get a t-test score

$$KS = \frac{\sum(X_{pos})/N_{pos} - \sum(X_{neg})/N_{neg}}{\sqrt{D^2(X_{pos})/N_{pos} + D^2(X_{neg})/N_{neg}}} \tag{1}$$

We furthermore developed regularized version of t-test, where prior variance was added separately to both variance estimates. This stabilizes the variance estimate similarly to work done in gene expression data analysis [10, 11]. Prior variance was defined as a variance of whole set of differential expression scores ($D^2(X_{total})$), multiplied with a weight w (for example $D^2(X_{pos}) = D^2(X_{pos}) + wD^2(X_{total})$). Five values of w $(0, 0.1, 0.3, 1, 3)$ were tested in artificial data analysis. The two largest weight values showed best performance and the largest $w = 3$ was selected to further analysis. One could potentially look for optimum weight value, or test other ways of adding 'a priori' variance to divider [10, 11] but this was considered to be outside the scope of this work. We later noticed that prior variance moves t-test towards novel scoring used by Newton et al. [12] (see supplementary text 2). This scoring function could be actually more stable representative from the signal summary methods.

Another intuitive and popular method is the Kolmogorov-Smirnov test (KS) [1, 13]. KS is based on Empirical Cumulative Distribution (ECD) of the class members and class non-members. It measures the difference between the two ECDs and takes the maximum absolute difference:

$$KS = max_{1 \leq j \leq N}(abs(\sum_{i=1}^{j} Y_i)) \tag{2}$$

where

$$Y_i = \frac{1}{K} \tag{3}$$

when gene belongs to class and

$$Y_i = \frac{-1}{L-K} \tag{4}$$

when gene does not belong to class. K represents the number class members (positive genes) and $L - K$ represents the number of negative genes. Recently a variation of the KS, modified KS (modKS), was presented that took the scores for the differential expression into account (similarly to our GSZ-score). Here the Y_i from the previous analysis is modified for class members to be:

$$modKS = max_{1 \leq j \leq N}(abs(\sum_{i=1}^{j} Y_i)) \tag{5}$$

where

$$Y_i = \frac{|X_i|^p}{\sum_j |X_j|^p} \tag{6}$$

The summation j goes over the class members. Otherwise method is same as KS test, especially with eq. (4) for class non-members. Although original publication used this method only with a specific differential expression score (Signal to Noise score, similar to t-test), we use it here with same differential expression scores as all the other methods.

One interesting method is iterative Group Analysis (iGA, [5]). In the original publication the gene list is analyzed using hypergeometric p-value to see whether the functional class is over-represented among the up-regulated genes. As the calculation of the hypergeometric p-value is computationally heavy task, the whole gene list was not analyzed. Instead the analysis was limited to threshold positions right below every class member, therefore greatly reducing the computation. Note that these are the positions at which the over-representation can be expected to be at maximum.

We have modified iGA considerably here. First, the statistic was modified to be two-tailed p-value, monitoring both over and under-representation of the gene class in the subset (score similar to one represented in [14]). Second, we included threshold positions right before and after the class member to the analysis. We can expect the over-representation to be at maximum right after the class member, but in

case of under-representation the maximum score should be right before the class member. With these two modifications our iGA analysis can analyze simultaneously with a single run gene classes over-represented at the upper end of the OGL (under-represented at the remaining lower part) and over-represented at the lower end of the list (under-represented at the remaining upper part). Original iGA algorithm would have required for this separate runs starting from upper and lower end. Third, we started monitoring the threshold positions next to the class non-members, when the class included more than half of the genes. This again lightens the calculation when the gene classes are large. Furthermore, we have used asymptotic methods (hypergeometric Z-score and binomial likelihood ratio) to select a smaller group of threshold positions that are most likely to represent a strongest signal, and calculate the hypergeometric p-value only for this smaller group. These also generated some speedup (although contribution of various speedups was not evaluated) and results were mostly consistent with the calculation of hypergeometric p-value.

We have omitted a group of earlier published methods. We have excluded the standard threshold based analysis of GO classes with hypergeometric distribution. This would require the definition of threshold for the analysis, and call for usage of many threshold values. Instead we have iGA that optimizes single threshold that shows the strongest signal with hypergeometric distribution. Nilsson et al. [4] represented Likelihood Ratio (LR) methods that were based on ECD, making these methods similar with KS test in our evaluation. These methods were quickly tested, but the observed results in the artificial data analysis were weak. This was probably due the problems while implementing them. LR methods were proposed for the better sensitivity at the tail areas [4]. Here we want to point that iGA in our comparison represents a well justified null model under row randomization (hypergeometric distribution) that should be able to monitor significant signals also occurring at the tail areas.

Also during the finishing of this manuscript we observed another novel method, Gene Set Analysis (GSA) [7]. Their method used a max-mean statistic, a compromise between absolute sum and sum of test scores. This is discussed in the supplementary text 2 (see additional data file 2).

Asymptotic and empirical p value calculation

Gene set analysis requires also the definition of the statistical significance for the observed results using p-values or Bayes factors. Previous works have mostly used empirical distribution to define p-value for the results, but this distribution has serious weaknesses: It is discontinuous, it is unreliable at the very tail, and requires large number of randomizations for reliable small p-values. Asymptotic distribution, on the

other hand, is continuous distribution and can be solved for arbitrary small p-values, although it is only an approximation of the true distribution and also dependent on the amount of randomized data available to learn the distribution parameters.

We generated empirical p-values similarly with all class scoring functions. Two principles have been earlier used with empirical p-value calculation: Most methods take only the randomization results reported for the evaluated class and use them to generate the empirical p-value [7, 9] (class data). GSEA represents the pooling of randomization results of all classes after their normalizations [3] (pooled data). Class data does not require any normalization and models exactly the distribution of the results for the class in question. However, class data generates small amount of randomization results for each class, and therefore it especially represents the weaknesses of empirical distribution. Pooled data represents larger number of random data points for null distribution, and shows therefore more stable p-values. However, it is strongly dependent on the normalization of the different classes. Here both these empirical p-values were used although frequently only pooled data was able to generate separation between the different scoring functions. In some cases, especially with class data, we obtained null as empirical p-value. As all our p-values are treated as -log10(p-value), these cases had to be corrected for inclusion to analysis. We did an 'ad hoc' correction by adding a pseudo count, 0.5, to p-value calculation in these cases.

We also represent asymptotic p-values for GSZ, although they were not used in the manuscript. As the maximum of the scores from the OGL is taken to analysis, it is natural to select extreme value distribution (EVD) or generalized extreme value distribution (GEVD) to model the asymptotic distribution under null hypothesis.

$$log(p\text{-}value_{EVD}) = -log(1 - exp(-exp(Y))) \quad (7)$$

$$log(p\text{-}value_{GEVD}) = -log(-[1 - exp(1 + \xi(-Y)]^{-1/\xi}) \quad (8)$$

Here $Y = (Mu - X)/Beta$. Mu and $Beta$ are the localization and scale parameters of the distribution. GEVD has also a shape parameter, ξ. These and other distributions were evaluated with cross validation using randomized real datasets (details omitted). Three best performing distributions (EVD, GEVD, gamma) and empirical p-value were selected to the further analysis. There each of the selected methods was tested using both class data and pooled data. Different methods were evaluated with randomized ALL data. Unfortunately no clear recommendations can be made from these results (data omitted). Therefore

we consider the p-value estimation and selection of suitable p-value measure as an open research topic, and propose monitoring both asymptotic and empirical p-values.

Ordering GO classes with p-values and other scores

Our evaluation of biological results from different scoring functions required the ordering of GO classes. We had four different empirical p-values obtained for each GO class with each method, which had to be combined into a single value for ordering. We excluded asymptotic p-values here as they were not available to all methods. We used two sets of p-values in combinations: $log(p\text{-}value)_{class}$ report the p-value from class data. $log(p\text{-}value)_{pooled}$ report the p-values obtained from pooled data. Furthermore we have p-values from column randomization ($log(p\text{-}value)^{col}$) and from row randomization ($log(p\text{-}value)^{row}$). We always used the weaker (larger p-value, smaller log(p-value)) of these two scores. Altogether, the combination can be represented as equation:

$$log(p\text{-}value)_{combined} = (min(log(p\text{-}value)^{col}_{class}, log(p\text{-}value)^{row}_{class}) + min(log(p\text{-}value)^{col}_{pooled}, log(p\text{-}value)^{row}_{pooled}))/2$$

Although the combination of p-values is ad hoc, it highlights classes that show good p-values under both row and column randomizations. It also combines the signal from both class specific and pooled randomizations. These combined p-values are later also later used to look for pairwise differences for scoring functions.

We also needed to order the results from competing program packages. This was especially required for GSA and GSEA, as these programs generated two result outputs, which in turn had to be combined into a single output. Original GSA package uses p-values for sorting, but due the ties in the empirical p-values, this is not optimal. Therefore we sorted GSA results always first with normalized scores, and next with p-values. This usually improved GSA performance in biological evaluation. GSEA program package reports several p-value or FDR estimates. We selected the normalized enrichment score (NES) for sorting the results. This is also used to sort classes in GSEA. Furthermore, we combined the up and down-regulated classes by using absolute value of NES. However, we point that there might be better sorting options also available in GSEA, but the different GSEA p-value estimates were unclear to us, and thus we did not use them. With SP we monitored the three available rankings and used mainly the default ranking.

Randomizations and normalization of the various methods

Methods were evaluated for potential biases by generating two types of randomizations (similar to earlier work [7, 9]). These were *row randomization*, where the labels of gene class are repetitively redistributed randomly along the gene list. This is the null hypothesis behind the mathematical model of GSZ-score and iGA. The other one is called *column randomization*. Here the differential gene expression scores are recalculated using permuted sample labels, like disease and control. Next the class scoring function is calculated using the recalculated differential expressions. Notice that row randomization does not require recalculation of the differential expression scores, and furthermore the results from this randomization depend only on the size of the class [4]. Therefore this randomization was calculated only once for every class size that was observed in the dataset. Column randomization is dependent on the genes included to the class and it was thus calculated separately for each class.

As the results show, some of the used methods represent bias in the row randomization. Furthermore all of them are expected to show biases in the column randomization. Therefore it is critical to normalize the obtained results, for example when pooling the randomizations for null distribution or when using the scores to order GO classes. Here one can use the results from the randomization with a standard Z-score type normalization:

$$X_{norm} = (X_{raw} - \mu)/\sigma \tag{9}$$

where σ and μ represent the estimated STD and mean, obtained from the row or column randomization of the gene class in question using the score to be normalized. Values for σ and μ can be simply the mean and the STD of the randomized scores. In addition, a different scaling was proposed earlier for KS and modKS ($X_{norm} = X_{raw}/\mu$, [3]). This was also tested, but the results altered only slightly, plus our visualizations of randomized data percentiles seemed less stable for this normalization. Therefore, we selected consistently identical normalization of all score functions. Although the different scorings might have different optimal normalizations with randomized data, figures 7 - 10 show quite stable distributions for each method from our normalization (see earlier chapters).

Previous scoring generates separate scores for row and column randomization. In biological analysis these could be analyzed also separately [9]. However, our real data evaluation using cross validation requires a single score for each GO class. We decided to select the minimum $X_{min} = min(X_{col}, X_{row})$ of the two

normalizations. This is based on the logic that the result should be robust against unwanted noise biases, potentially seen only under row or column randomizations (see [7] for added discussion). Furthermore, this relates to the risk of having too small STD estimate (like in gene expression data analysis) in one of the randomizations, that can generate too large signals. Another option for combining the two null-hypotheses is represented by Efron and Tibshirani [7].

Gene expression datasets

Discussed methods have been tested on various gene expression datasets (Toronen et al. and Ojala et al. unpublished research), but we limit our analysis only to few datasets, as we focus on the method comparison. For our preliminary analysis steps we used the Diabetes dataset, obtained from the GSEA website [15]. We used a simple t-test to obtain differential expression scores. The GO annotations were obtained by using the open access Gene ID-Converter [16]. We also obtained our p53 dataset from the same web site. The only difference in its preprocessing was that we used IBMT [11], an improved version of LIMMA method [10], for obtaining the differential expression scores.

The real data evaluation with split data required high quality gene expression data. Especially the consistency in the signals within the compared sample groups was essential. With lower quality datasets the dissimilarities between the samples causes the correlation between the OGLs on the functional class level to disappear. Furthermore we need a good number of replicates within the sample groups, so that the column randomizations can be performed adequately even when the dataset is split. Therefore we selected ALL (Acute Lymphatic Leukaemia) dataset [17], and decided to monitor the differences between the B cell and T cell based leukaemias.

In order to obtain the highest data quality, we started the analysis from the Affymetrix CEL files using Bioconductor package [18]. Probesets were remapped to genes using reannotations [19], and the data was then pooled to single gene expression measures using RMA [20]. The quality of the obtained gene expression data was evaluated by visualizing the between sample correlations, which corresponded nicely with the analyzed sample groups (data not shown). We used IBMT again to generate the differential expression scores.

ALL data probe sets were mapped to Entrez gene Ids. The GO classifications for these were obtained from two sources, Protein Information Resource [21] and ID-Converter [16]. Two sources were joined by using all

the annotations that were reported for either GO classification source. This aims at large coverage of genome but it can potentially generate more false positive classifications than either source separately. Differently from the earlier work we decided to include all the GO classes that were larger than 2 genes. Furthermore, no upper limit on the GO class size was used. This way the dataset allows better monitoring of size dependent biases, although in real biological testing it would be reasonable to limit the class sizes to more informative classes.

Splitting of gene expression data

ALL dataset was repetitively split to two halves. The two halves were used as separate learning and testing datasets in cross validation evaluation. This was done by splitting the RMA expression values for two sample groups (B cell and T cell leukaemias) separately into two as equally sized groups as possible. Repetitive splits were selected from a larger pool of random splits, so that all the splits are as dissimilar as possible (Toronen unpublished method). Next the analysis pipeline (IBMT, each analysis method, row and column randomizations) was performed separately in parallel using always only a one half of the available information.

Artificial data evaluation

In artificial data evaluation we compare various functions for their ability to separate classes with positive signal from the random background variance. The parameters for the positive signal were varied on a wide range to generate a comprehensive picture on best performing methods in different signal areas. Standard normal distribution was selected as a background null distribution. This was used for sampling the gene expression scores for class non-members. The positive signal (for class members) was also sampled from a normal distribution, but with adjustable variance and mean. In addition, only a subset of genes from the functional class was selected to represent the signal, while the rest of them had expression scores sampled from the null distribution. Furthermore, the size of the functional class was varied. The background variance was obtained by analyzing each generated dataset with few row randomizations. Similarly the changes in the signal representing part of the gene class were generated by randomizing the labels for a subset of the genes from the functional class.

All the previous steps are similar to earlier work [4, 13]. Our enhancement is that we have also separately tested simultaneous up and down-regulation of functional class. This was done by defining the sign for the

positive signal, from the binomial distribution (with probability 0.5). In addition each testing was done also separately in two ways: 1) Comparing positive signals for each class size to randomized signals from the very same class size. 2) Comparing the positive signal from varying sizes of classes to randomized signals from similarly varying classes. We want to point that the testing with varying class size includes unsolved problems. Especially, the signal was kept at same magnitude although the class size was allowed to vary. This is problematic as the large class with equal sized signal is always easier to detect than a small class. Therefore the signal level should probably be shifted to be larger for smaller classes, for maintaining the consistent level of difficulty. Due to the associated problems we give less weight to these results and consider them to be suggestive. Nevertheless, this problem should affect each method's results similarly.

Table 7: Parameters used in the artificial data analysis

Size of the class (A)	Size of the class (B)	STD of the signal	mean of the signal	portion of the class representing signal
5	5	0.25	0.25	0.1
20	50	0.5	0.50	0.2
50	500	1	0.75	0.3
200		2*	1.00	0.4
500			1.25	0.5
			1.50	0.6
			1.75	0.7
			2.00	0.8
			2.25	0.9
			2.50	1
			2.75	
			3.00	

Different parameters of the artificial data and values used for them. Size of the class (A) refers to the case where the testing was always carried out against the same class size. Size of the class (B) shows class sizes used in testing, where different class sizes are pooled together before the comparison. The largest value of STD (2) was omitted from the actual summation of the results. Mean of the signal was either positive (up-regulation) or defined randomly as positive or negative (simultaneous up and down-regulation). All parameters were used in all combinations.

Derivation of $E(S|N)$ and $D^2(S|N)$

Here we derive equations required for the GSZ-score mean and variance. These represent the analysis situation where we have a fixed number of, for example, positive genes, and a larger set of differential expression scores (for both positive and negative genes). Our aim is to define mean and variance estimates for the sum of score values for positive genes, when they are randomly sampled from the larger pool. Notice the difference to our mean and STD estimates of GSZ-score, that do not need fixed number, and take into account the variance in the number of positive genes also.

Let X_i, $i = 1, \ldots, n$ be i.i.d. random variables representing the values of a subgroup of all the N differential expression test scores. Let the expected value and the variance of each X_i be determined by the sample expectation and variance for the whole population of N test scores. Our goal is to derive a formula for the variance of $X = \sum_{i=1}^{n} X_i$, the sum of the n differential expression scores. The expected value of X is given by

$$\begin{aligned} E\left[X\right] &= E\left[\sum_{i=1}^{n} X_i\right] = \sum_{i=1}^{n} E\left[X_i\right] \\ &= n * E\left[X_1\right] = n * \overline{x}, \end{aligned}$$

where $\overline{x}$ equals

$$\overline{x} = \frac{1}{N}\sum_{i=1}^{N} x_i,$$

the sample estimate based on all the N differential expression test scores. The expected value of X^2 can be

written as

$$
\begin{aligned}
E\left(X^2\right) &= E\left[\left(\sum_{i=1}^{n} X_i\right)^2\right] \\
&= E\left[\sum_{i=1}^{n} X_i^2\right] + E\left[\sum_{i=1}^{n}\sum_{\substack{j=1\\ j\neq i}}^{n} X_i X_j\right] \\
&= n * E\left[X_1^2\right] + n(n-1) * E\left[X_1 X_2\right] \\
&= n * E\left[X_1^2\right] + n(n-1) * \sum_{i=1}^{N}\sum_{\substack{j=1\\ j\neq i}}^{N} \frac{1}{N(N-1)} x_i\, x_j \\
&= n * E\left[X_1^2\right] + \frac{n(n-1)}{N\,(N-1)} * \sum_{i=1}^{N} x_i \sum_{\substack{j=1\\ j\neq i}}^{N} x_j \\
&= n * E\left[X_1^2\right] + \frac{n(n-1)}{N\,(N-1)} * \sum_{i=1}^{N} x_i \left[\left(\sum_{j=1}^{N} x_j\right) - x_i\right] \\
&= n * E\left[X_1^2\right] + \frac{n(n-1)}{N\,(N-1)} * \left(\sum_{i=1}^{N} x_i\right)^2 - \frac{n(n-1)}{N\,(N-1)} * \sum_{i=1}^{N} x_i^2 \\
&= n * E\left[X_1^2\right] + \frac{n(n-1)N}{(N-1)} \overline{x}^2 - \frac{n(n-1)}{(N-1)} * E\left[X_1^2\right] \\
&= E[X_1^2]\left(\frac{n\,(N-n)}{N-1}\right) + \frac{n(n-1)N}{(N-1)} \overline{x}^2.
\end{aligned}
$$

Thus, the variance of X can be written as

$$
\begin{aligned}
Var\left[X\right] &= E\left[X^2\right] - E\left[X\right]^2 \\
&= E[X_1^2]\left(\frac{n\,(N-n)}{N-1}\right) + \frac{n(n-1)N}{N-1} \overline{x}^2 - n^2 \overline{x}^2 \\
&= \left(\frac{n\,(N-n)}{N-1}\right) * \left(E\left[X_1^2\right] - \overline{x}^2\right) \\
&= \left(\frac{n\,(N-n)}{N-1}\right) * Var(X_1)
\end{aligned}
$$

Pseudo code representation of analysis steps

We show outline for GSZ analysis steps as pseudo codes in fig. 13. We also represent the outline for the cross validation style function comparison in fig. 16. Other pseudo code figures show analysis steps used by these analysis pipelines. More detailed discussion on these steps is available in the actual manuscript.

GSZ Analysis Steps

- Gene Expression (GE) data Matrix (N genes and M samples)
- Classifications (like disease and control) for samples in GE data
- GO classifications for genes in the GE matrix (N genes and L classes)

STEPS

Run GSZ Signal Analysis
Run Column Randomization
Calculate p-value p_{col} for each class using its Column Randomizations
Run Row Randomization
Calculate p-value p_{row} for each class using its Row Randomizations
Select weaker (=larger) p-value as a result: p = $max(p_{col}, p_{row})$
Sort classes using the p-values, and/or select significant classes

GSZ Signal Analysis

INPUT

- Gene Expression (GE) data Matrix (N genes and M samples)
- Classifications (like disease and control) for samples in GE data
- GO classifications for genes in the GE matrix (N genes and L classes)

STEPS

Generate Differential Expression (DE) scores (with t-test or LIMMA etc.) for genes between the two sample groups.
Sort genes using DE scores.
for $j = 1..L$ **do**
  Calculate GSZ-score at each threshold position of the gene list, using the jth GO class and the DE scores.
  return the threshold position with the largest absolute score as output score
end for

Figure 13: **Representation of GSZ-score analysis steps.** Figure represents steps required for the analysis of the GSZ-score with differential gene expression analysis. First test statistic (DE score,) can be switched in other applications to another more suitable statistic. Notice that for statistical significance the analysis has to be replicated with row and/or column randomization. These are represented later as they were implemented in function comparison. Reported p-values can be calculated either with each class' empirical distribution or by fitting an asymptotic distribution to empirical distribution.

FUNCTION COMPARISON

INPUT

- Gene Expression (GE) data Matrix (N genes and M samples)
- Classifications (like disease and control) for samples in GE data
- GO classifications for genes in the GE matrix

STEPS

Generate Differential Expression (DE) scores (with t-test or LIMMA etc.) for genes between the two sample groups.
Sort genes using DE scores (for methods that require sorted list)
for $j = 1..L$ **do**
Obtain the results from compared test functions using jth GO class and DE-scores and/or sorted list:

- Calculate GSZ-test
- Calculate t-test
- Calculate KS-test
- Calculate modKS-test
- Calculate iGA

end for
return each methods results for each analyzed class

Figure 14: **Representation of function comparison analysis steps.**

COLUMN RANDOMIZATION

INPUT

- Gene Expression (GE) data Matrix (N genes and M samples)
- Classifications (like disease and control) for samples in GE data
- GO classifications for genes in the GE matrix
- K, the required number of randomizations

STEPS

Generate K different randomizations to sample labels
for $j = 1..K$ **do**
  Run FUNCTION COMPARISON using jth randomization of sample labels and correct GO data.
  Store obtained random results for each class from each function.
end for
return K random results from each method for each analyzed class

ROW RANDOMIZATION

INPUT

- Gene Expression (GE) data Matrix (N genes and M samples)
- Classifications (like disease and control) for samples in GE data
- GO classifications for genes in the GE matrix
- K, the required number of randomizations

STEPS

Generate K different randomizations to GO classifications*
for $j = 1..K$ **do**
  Run FUNCTION COMPARISON using jth randomization of GO classifications and correct sample labels.
  Store obtained random results for each class from each function.
end for
return K random results from each method for each analyzed class

Figure 15: **Representation of two randomizations.** Figure represents an outline for Row and Column Randomization as it is implemented in function comparison. Note that the compared methods are treated identically against the same set of randomizations. *One can either generate K different row randomizations of the GO data matrix or generate K different randomizations for each class size. In this work we selected the latter lighter option. However, note that it cannot be used to estimate the GO class correlations and False Discovery rates from the dataset.

Evaluation with Cross Validation

INPUT

- Gene Expression (GE) data Matrix (N genes and M samples)
- Classifications (like disease and control) for samples in GE data
- GO classifications for genes in the GE matrix

STEPS

1: **for** $j = 1..4$ **do**
2: Split the disease samples randomly into two appr. equal sized groups A1 and A2
3: Split the disease samples randomly into two appr. equal sized groups B1 and B2
4: Run Method Comparison, Row Randomization and Column Randomization using only data in A1 and B1.
5: Generate a Z-score (Z_{row}) from positive results of each method for each GO class using the mean and STD estimates from each method's row randomization results.
6: Generate a Z-score (Z_{col}) from positive results of each method for each GO class using the mean and STD estimates from each method's column randomization results.
7: Select the weaker of two Z-scores ($min(Z_{row}, Z_{col})$) as a result for each class with each method. This gives us result vectors: $R1_{GSZ}$, $R1_{t\text{-}test}$, $R1_{KS}$, $R1_{modKS}$, $R1_{iGA}$.
8: Repeat the steps 4 - 7 using only data in A2 and B2 to obtain the result vectors: $R2_{GSZ}$, $R2_{t\text{-}test}$, $R2_{KS}$, $R2_{modKS}$, $R2_{iGA}$.
9: **for** $test = GSZ, t\text{-}test, KS, modKS, iGA$ **do**
10: Run AUC Test with Varying Positive Subset to test $R1_{test}$ using $R2_{test}$ as control set. Store as $result1_i$.
11: Run AUC Test with Varying Positive Subset to test $R2_{test}$ using $R1_{test}$ as control set. Store as $result2_i$.
12: **return** $AUC\_vector^{test}_{case\ i} = mean(result1_i, result2_i)$
13: Calculate the rank correlation between $R1_{test}$ and $R2_{test}$. **Return** $Corr^{test}_{i\ rank}$.
14: Calculate the Pearson correlation between $R1_{test}$ and $R2_{test}$. **Return** $Corr^{test}_{i\ Pearson}$.
15: Run Combination of Control Sets using all $R2$ results in combination to obtain $Rank2$
16: Run Combination of Control Sets using all $R1$ results in combination to obtain $Rank1$
17: Run AUC Test with Varying Positive Subset to test $R1_{test}$ using $Rank2$ as control set. Store as $result1_{ii}$.
18: Run AUC Test with Varying Positive Subset to test $R2_{test}$ using $Rank1$ as the control set. Store as $result2_{ii}$.
19: **return** $AUC\_vector^{test}_{case\ ii} = mean(result1_{ii}, result2_{ii})$
20: Generate a ranking of GO classes ($Rank1_{test}$) from $R1_{test}$ starting from the highest score.
21: Generate a ranking of GO classes ($Rank2_{test}$) from $R2_{test}$ starting from the highest score.
22: calculate a rank correlation using $Rank1_{test}$ and $Rank2$. **Return** $Corr1^{test}_{ii\ rank}$
23: calculate a rank correlation using $Rank2_{test}$ and $Rank1$. **Return** $Corr2^{test}_{ii\ rank}$
24: **end for**
25: **end for**

Figure 16: **Representation of evaluation using cross-validation.** The used data is split to two halves. Each half is analyzed with different functions with exactly same DE scores and with exactly same set of randomizations. Next each function's results are compared against the control (*Gold Standard*) generated with the the same function from the other half (case i) or by using all the compared functions in combination (case ii). Some of the used sub-functions are

Combination of Control Sets

INPUT

- a matrix of GO class Z-scores (methods on row and GO classes in columns) to be used as a control

Generate a ranking of GO classes ($rank_{ctrl}$), using control result matrix, separately for each row, starting with the strongest score.
Select the smallest rank from all methods as the rank for each GO class.
return vector of generated ranks.

AUC Test with Varying Positive Subset

INPUT

- a vector of GO class Z-scores to be tested
- a vector of GO class Z-scores to be used as a control **OR**
- a vector of GO class ranks precalculated from the control dataset.

STEPS

Generate a ranking of GO classes ($rank_{test}$), starting with the largest score.
if control set represents Z-scores **then**
  Generate a ranking of GO classes ($rank_{ctrl}$), with control list, starting with strongest score.
else
  take the control input directly as $rank_{ctrl}$.
end if
for $j = 1..200$ **do**
  Select classes that have $rank <= j$ as positive classes. Set all the remaining classes as negative.
  Calculate AUC score between positive - negative classification and $rank_{test}$, where the small ranks are considered as positive.
  store the result in output vector ($AUC[j] = score$)
end for
return AUC vector

Figure 17: **Outline of two sub-functions used in cross-validation.** Figure presents the steps used in the AUC (Area Under Curve) analysis. Difference to normal AUC analysis is that the positive and negative GO classes are not known. Therefore the control list is used to define j best scoring classes as positive.

References

1. Mootha VK, Lindgren CM, Eriksson KF, Subramanian A, Sihag S, et al. (2003) PGC-1alpha-responsive genes involved in oxidative phosphorylation are coordinately downregulated in human diabetes. Nature genetics 34: 267-273.
2. Christian W (1996) Hand-book of statistical distributions for experimentalists. Technical Report SUF-PFY/96-01, Particle Physics Group; Stockholm University.
3. Subramanian A, Tamayo P, Mootha VK, Mukherjee S, Ebert BL, et al. (2005) Gene set enrichment analysis: a knowledge-based approach for interpreting genome-wide expression profiles. Proceedings of the National Academy of Sciences of the United States of America 102: 15545-15550.
4. Nilsson B, Hakansson P, Johansson M, Nelander S, Fioretos T (2007) Threshold-free high-power methods for the ontological analysis of genome-wide gene-expression studies. Genome biology 8: R74.
5. Breitling R, Amtmann A, Herzyk P (2004) Iterative Group Analysis (iGA): a simple tool to enhance sensitivity and facilitate interpretation of microarray experiments. BMC bioinformatics 5: 34.
6. Goeman JJ, Buhlmann P (2007) Analyzing gene expression data in terms of gene sets: methodological issues. Bioinformatics 23: 980-987.
7. Efron B, Tibshirani R (2007) On testing the significance of sets of gene. Annals of Applied Statistics 1: 107-129.
8. Breslin T, Eden P, Krogh M (2004) Comparing functional annotation analyses with Catmap. BMC bioinformatics 5: 193.
9. Tian L, Greenberg SA, Kong SW, Altschuler J, Kohane IS, et al. (2005) Discovering statistically significant pathways in expression profiling studies. Proceedings of the National Academy of Sciences of the United States of America 102: 13544-13549.
10. Lönnstedt I, Speed TP (2002) Replicated microarray data. Statistica Sinica 12: 31-46.
11. Sartor MA, Tomlinson CR, Wesselkamper SC, Sivaganesan S, Leikauf GD, et al. (2006) Intensity-based hierarchical Bayes method improves testing for differentially expressed genes in microarray experiments. BMC bioinformatics 7: 538.
12. Newton MA, Quintana FA, Boon JAD, Sengupta S, Ahlquist P (2007) Random-set methods identify distinct aspects of the enrichment signal in gene-set analysis. Annals of Applied Statistics 1: 85-106.
13. Ben-Shaul Y, Bergman H, Soreq H (2005) Identifying subtle interrelated changes in functional gene categories using continuous measures of gene expression. Bioinformatics 21: 1129-1137.
14. Toronen P (2004) Selection of informative clusters from hierarchical cluster tree with gene classes. BMC bioinformatics 5: 32.
15. Gsea web site. URL http://www.broad.mit.edu/gsea/.
16. Alibes A, Yankilevich P, Canada A, Diaz-Uriarte R (2007) IDconverter and IDClight: conversion and annotation of gene and protein IDs. BMC bioinformatics 8: 9.
17. Chiaretti S, Li X, Gentleman R, Vitale A, Wang KS, et al. (2005) Gene expression profiles of B-lineage adult acute lymphocytic leukemia reveal genetic patterns that identify lineage derivation and distinct mechanisms of transformation. Clinical cancer research 11: 7209-7219.
18. Gentleman RC, Carey VJ, Bates DM, Bolstad B, Dettling M, et al. (2004) Bioconductor: open software development for computational biology and bioinformatics. Genome biology 5: R80.
19. Dai M, Wang P, Boyd AD, Kostov G, Athey B, et al. (2005) Evolving gene/transcript definitions significantly alter the interpretation of GeneChip data. Nucleic acids research 33: e175.
20. Irizarry RA, Bolstad BM, Collin F, Cope LM, Hobbs B, et al. (2003) Summaries of Affymetrix GeneChip probe level data. Nucleic acids research 31: e15.
21. Barker WC, Garavelli JS, Huang H, McGarvey PB, Orcutt BC, et al. (2000) The protein information resource (PIR). Nucleic acids research 28: 41-44.
