# Supplementary material for: Robust extraction of functional signals from gene set analysis using a generalized threshold free scoring function
Source: BMC Bioinformatics. 2009 Sep 23;10:307. doi: 10.1186/1471-2105-10-307 (PMC2761411; doi:10.1186/1471-2105-10-307)
Supplement: Additional file 2 — Supplementary text S2. This text represents a mathematical comparison of the GSZ-score with hypergeometric Z-score, with correlation between the differential expression scores and GO classification, with max-mean score (by Efron & Tibshiriani) and with Random Sets scoring function by Newton et al. [file 1471-2105-10-307-S2.PDF]

Mathematical comparison of Gene Set Z-score with similar analysis functions

Petri Törönen *[1], Pauli Ojala[2] , Pekka Marttinen[3], Liisa Holm[14]

[1]The Holm Group, Biocenter II, Institute of Biotechnology, PO Box 56, 00014 University of Helsinki, Finland
[2]Finnish Red Cross Blood Service Research and Development, Kivihaantie 7, 00310 Helsinki, Finland
[3]Department of Mathematics and Statistics, P.O. Box 68, 00014 University of Helsinki, Finland
[4]Department of Biological and Environmental Sciences, P.O. Box 56, 00014 University of Helsinki, Finland

Email: Petri Toronen*- firstname.lastname@helsinki.fi; Pauli Ojala - firstname.lastname@bts.redcross.fi; Pekka Marttinen - firstname.lastname@helsinki.fi; Liisa Holm - firstname.lastname@helsinki.fi;

*Corresponding author

Short note

This is the second supplementary text for the manuscript 'Novel Robust Universal Scoring Function for Threshold Free Gene Set Analysis'. Text represents detailed comparison of the proposed method with other similar methods. This highlights the similarities with more standard methods and recent publications and shows that GSZ score represents a theoretical unification that encompasses these compared methods. Also a graphical representation of the similarities is represented.

GSZ-score calculated using the whole list is similar to the correlation of GO class and gene expression data

Our main text presents the Gene Set Z-score (GSZ-score) for analysis of subsets of genelists for the GO class related signals. It starts with a score:

$$Diff_M = \sum_{\substack{i \in S_M \\ i \in pos}} X_i - \sum_{\substack{i \in S_M \\ i \in neg}} X_i, \quad (1)$$

$$= \sum_M X_{pos} - \sum_M X_{neg} \quad (2)$$

a difference between the sums of scores for members (positive gene group pos) and non-members (neg) of a gene class, among the M genes with the highest differential expression test scores. For simplicity, we omit later the index M from $Diff_M$. The equation 1 is more stable as a Z-score:

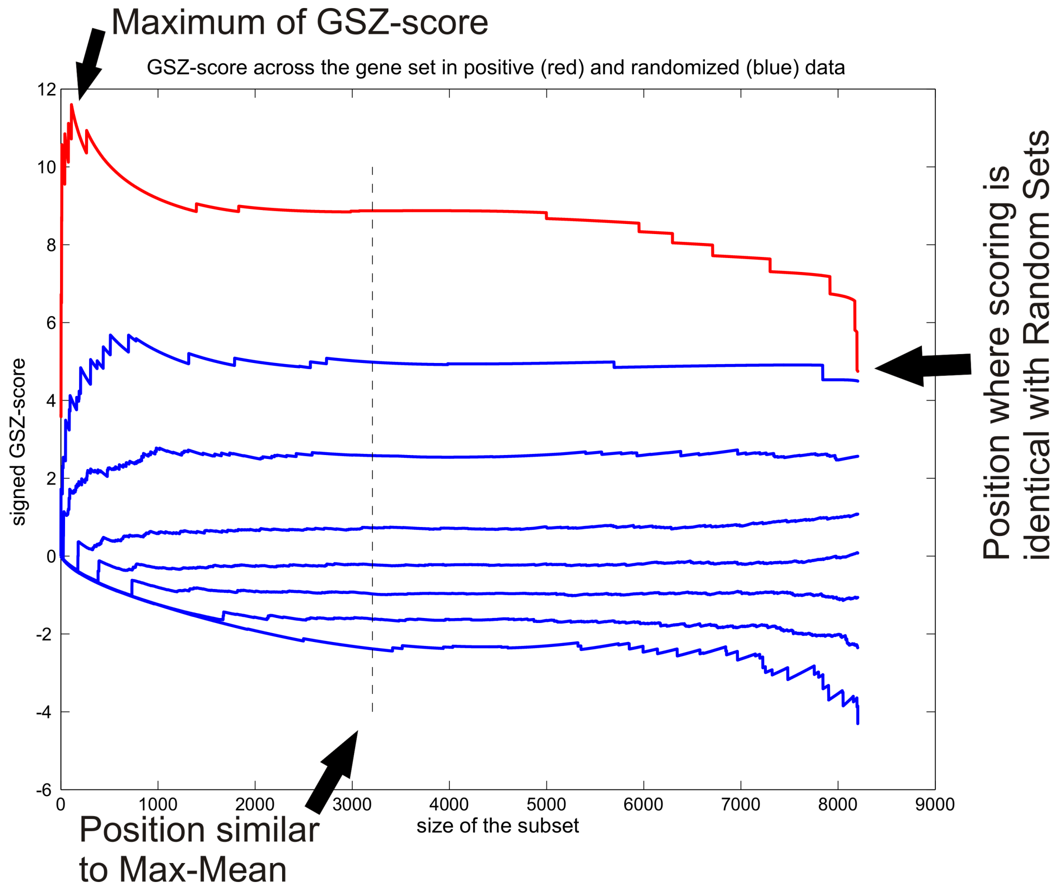

Figure 1: **Visualization of the similarities between GSZ-score, Max-Mean and Random Sets.** Figure represents the GSZ-score profile for ALL dataset (red line) for GO class 'immunological synapse'. Profile is calculated over the ordered gene list. Figure also shows seven percentiles (blue lines), obtained from 500 column randomizations of the same class and dataset. Percentiles are: 0, 5, 25, 50, 75, 95, and 100. Notice the clear separation of the positive curve with the randomization results. In addition figure shows an approximate position that corresponds with Max-Mean scoring. This position is not necessarily exactly same across randomizations. Furthermore, Max-Mean lacks the stabilization performed with GSZ-score. Figure also shows the last point, where GSZ-score is identical with Random Sets scoring function and similar with correlation. This class shows simultaneous up and down-regulation resulting in a small average expression signal, seen at the last point.

$$Z = \frac{Diff - E(Diff)}{\sqrt{D^2(Diff) + k}}, \tag{3}$$

where $E(Diff) = 2E(X)E(N) - ME(X)$ and

$$D^2(Diff) = 4(\frac{D^2(X)}{M-1}(E(N)(M - E(N)) - D^2(N)) + E(X)^2 D^2(N)) \tag{4}$$

where N is the number of positive genes in the subset, M is the size of the subset, $E(N)$ is the mean and $D^2(N)$ is the variance of the hypergeometric distribution of the number of positive genes N for the analyzed subset. $D^2(X)$ and $E(X)$ are the variance and the mean of the primary test scores for the subset. k represents the prior variance used to stabilize the function (see main text). The derivation of $E(Diff)$ and $D^2(Diff)$ is shown in the main text.

As a difference to earlier methods (Kolmogorov-Smirnov, modified Kolmogorov-Smirnov, iGA), the signal can be calculated using the whole gene list ($M = L$). In that case the variance and mean of the resulting hypergeometric distribution are $D^2(N) = 0$ and $E(N) = K$, and the variance estimate of $Diff$ is reduced to:

$$\begin{aligned} D^2(Diff) &= 4(\frac{D^2(X_i)}{M-1}(E(N)(M - E(N)) - D^2(N)) \\ &+ E(X)^2 D^2(N)) \\ &= \frac{4D^2(X_i)K(L-K)}{L-1} \end{aligned} \tag{5}$$

The expectation of the $Diff$ is similarly reduced to

$$\begin{aligned} E(Diff) &= 2E(X)E(N) - ME(X) \\ &= (2K - L)E(X) \end{aligned} \tag{6}$$

Equation (5) is actually identical to the $D^2(S|N)$ derived in the supplementary text 1 [see additional file 1] that is used in the derivation of the variance equation. A more detailed view on this equation shows that it can be considered as a function of two components: $D^2(X)$ and $K(L - K)/(L - 1)$. The first one represents the variance of the test scores and the latter reminds variance of binary classification to class members (K) and non-members ($L - K$). Similarly the equation (6) is reduced to the product of the expectation of a binary classification ($2K - L$) and expectation of the differential expression values ($E(X)$). In overall, the whole equation becomes:

$$
\begin{aligned}
Z &= \frac{\sum X_{pos} - \sum X_{neg} - E(Diff)}{\sqrt{D^2(Diff)}} \\
&= \frac{\sum X_{pos} - \sum X_{neg} - (2K - L)E(X)}{2\sqrt{(D^2(X_i)K(L-K))/(L-1)}} \\
&= \frac{\sum X_{pos} - (L * E(X) - \sum X_{pos}) - (2K - L)E(X)}{2\sqrt{(D^2(X_i)K(L-K))/(L-1)}} \\
&= \frac{2\sum X_{pos} - 2KE(X)}{2\sqrt{(D^2(X_i)K(L-K))/(L-1)}} \qquad (7)
\end{aligned}
$$

We used the rule $L * E(X) = \sum X_{pos} + \sum X_{neg}$, where $\sum X_{pos}$ is a sum of expression values for positive genes (class members) taken from all data. This shows that Z-score is in this case very similar to the correlation between the class-member classification (binary variable Y) and the given differential expression test scores (X). For comparison the correlation is:

$$
\begin{aligned}
Corr &= \frac{E(XY) - E(X)E(Y)}{\sqrt{D^2(X)D^2(Y)}} \qquad (8) \\
&= \frac{\sum X_{pos} - E(X)K}{\sqrt{D^2(X)L(K/L)(L-K)/L}} \qquad (9) \\
&= \frac{\sum X_{pos} - E(X)K}{\sqrt{D^2(X)K(L-K)/L}} \qquad (10)
\end{aligned}
$$

Here we have used assumptions $E(Y) = K$ and $D^2(Y) = np(1-p) = L(K/L)((L-K)/L)$. Note that we omit from this comparison the contribution of the prior variance k. This is done to simplify the comparison of equations. Also, our detailed analysis shows that the prior variance is mostly clearly smaller than the estimated variance (data not shown), so it should only contribute a small stabilizing effect for the smallest variances. Notice that this case corresponds also to the scoring function represented by Newton et al. We discuss the differences between these methods later.

Gene Set Z-score can be identical to Hypergeometric Z-score

Another extreme case would occur, if we keep the order of the genes in the list but omit the test scores from our analysis and give to each gene a weight equal to unity. In this case $D^2(X) = 0$, $E(X) = 1$, and the variance estimate reduces to $D^2(S) = E(X)^2 D^2(N) = D^2(N)$. So only the variance for the hypergeometric distribution remains. The expectation value for the sum changes similarly:

$E(Diff) = 2E(X)E(N) - ME(X) = 2E(N) - M$. The resulting Z-score is:

$$
\begin{aligned}
Z_{raw} &= \frac{\sum X_{pos} - \sum X_{neg} - E(Diff)}{\sqrt{D^2(Diff)}} \\
&= \frac{\sum X_{pos} - \sum X_{neg} - (2E(N) - M)}{2\sqrt{(D^2(N)}} \\
&= \frac{\sum X_{pos} - (M - \sum X_{pos}) - 2E(N) + M}{2\sqrt{(D^2(N)}} \\
&= \frac{2N - 2KE(N)}{2\sqrt{(D^2(N)}} \qquad (11)
\end{aligned}
$$

This is simply the hypergeometric Z-score for a variable N. We have again omitted the prior variance for simplicity. Similar phenomenon occurs (in theory) when data subset includes only a single data point ($M = 1$, details not shown). Still a drawback in $M = 1$ case is that the equation is not defined due the term $M - 1$ in divider (generating 0/0). Due to this, we omit this case from analysis. Note that the hypergeometric distribution is currently probably the most popular method for analyzing gene lists using a threshold.

The reasonable results obtained with iGA (hypergeometric p-value based analysis) propose that a simple analysis of order would also be useful. This could be obtained by ordering the gene list with gene expression values, and then giving to each gene the expression value unity.

Max-Mean statistic reminds Gene Set Z-score

The score proposed by Efron & Tibshiriani was a max-mean statistic, where the analyzed gene set (positive genes) is divided into a half at $X = 0$. This corresponds to our Gene Set Z-score analysis with the pre-positioning of the threshold at the exactly same location. Their score is based on sum of the expression values for both halves (half with positive regulation, half with negative regulation), next it divides each half with the whole size of the gene set. The bigger of the absolute values for two halves is selected as the score.

$$
MaxMean = max(abs(\sum_{\substack{X_i>0 \\ i\in pos}} X_i), abs(\sum_{\substack{X_i<0 \\ i\in pos}} X_i))/N_{pos} \qquad (12)
$$

This reminds our Z-score analysis as our analysis of differences can be considered as a function of sum of scores for positive genes. The differences are: (a) Max-mean scoring omits totally the class non-members

and potentially the large expression signals among them. This was corrected by running several row randomizations of expression data. We propose the usage of direct estimates of mean and STD for the selected subset. Our estimates take directly into account the deviation in the gene expression data, among both the members and non-members. Our estimates could be also directly used to stabilize the max-mean function. (b) With max-mean the subset with larger absolute sum is taken as the score. This does not take into the consideration the potentially larger overall deviation from the null in the other tail, or even the potential bias where the whole expression distribution would be shifted up or down. Thus, in such cases max-mean would be potentially unable to report the signals, occuring in the tail area with weaker overall signal. This could be again corrected by using our mean and STD estimates for the selected subsets. (c) Max-mean is used with a pre-fixed threshold position, whereas we propose the optimization of the threshold position so that the maximum absolute signal is obtained. While both of these methods have pros and cons, it might be still beneficial for the max-mean type scoring to allow the threshold to move at least within some frame. This way the predefined threshold would not affect the results so much. As an example, the analyzed datasets outside the actual differential gene expression analysis can include skewed distributions including only positive values. The used null threshold is clearly unusable with such datasets, and the selection of new useful threshold might be challenging in those cases. Note still that the work of Efron and Tibshiriani propose that one could use also GSZ-score with some pre-defined threshold positions.

Score function by Newton et al. is GSZ-score calculated from the whole gene list

While finishing this manuscript we were pointed to the publication by Newton et al. They represent similar method as ours. Their scoring starts with $\bar{X} = \sum_N X_{pos}/N$, a mean value of differential expression for all the genes that belong to the analyzed class. Next, the mean and variance estimates for $\bar{X}$ are represented. These are practically the same equations as our equations for $E(S|N)$ and $D^2(S|N)$, with only differences caused by division with N. So their scoring function corresponds GSZ score, when it is calculated using the whole gene list (equation 7). The major difference between these methods is that GSZ-score can monitor simultaneous up and down-regulation, as it goes through the gene list.
