# Supplementary material for: Robust extraction of functional signals from gene set analysis using a generalized threshold free scoring function
Source: BMC Bioinformatics. 2009 Sep 23;10:307. doi: 10.1186/1471-2105-10-307 (PMC2761411; doi:10.1186/1471-2105-10-307)
Supplement: Additional file 3 — Supplementary figure S1: Stability of the GSZ-score as the threshold goes through the gene list. Distribution of the GSZ-score values as the threshold is moved along the gene list. Subset is smallest at the left and largest (the whole gene list) at the right end of the plot. Results are calculated using all the 4511 GO classes from diabetes dataset with randomized GO class matrix. Blue lines show seven percentiles (0, 5, 25, 50, 75, 95, and 100) at each position. For comparison, the red line shows minimum and maximum scores from the non-randomized diabetes dataset. Notice the good stability with the regularized GSZ-scores. Figure is discussed more in a more detail in the supplementary text S1 [see additional file 1]. [file 1471-2105-10-307-S3.PDF]

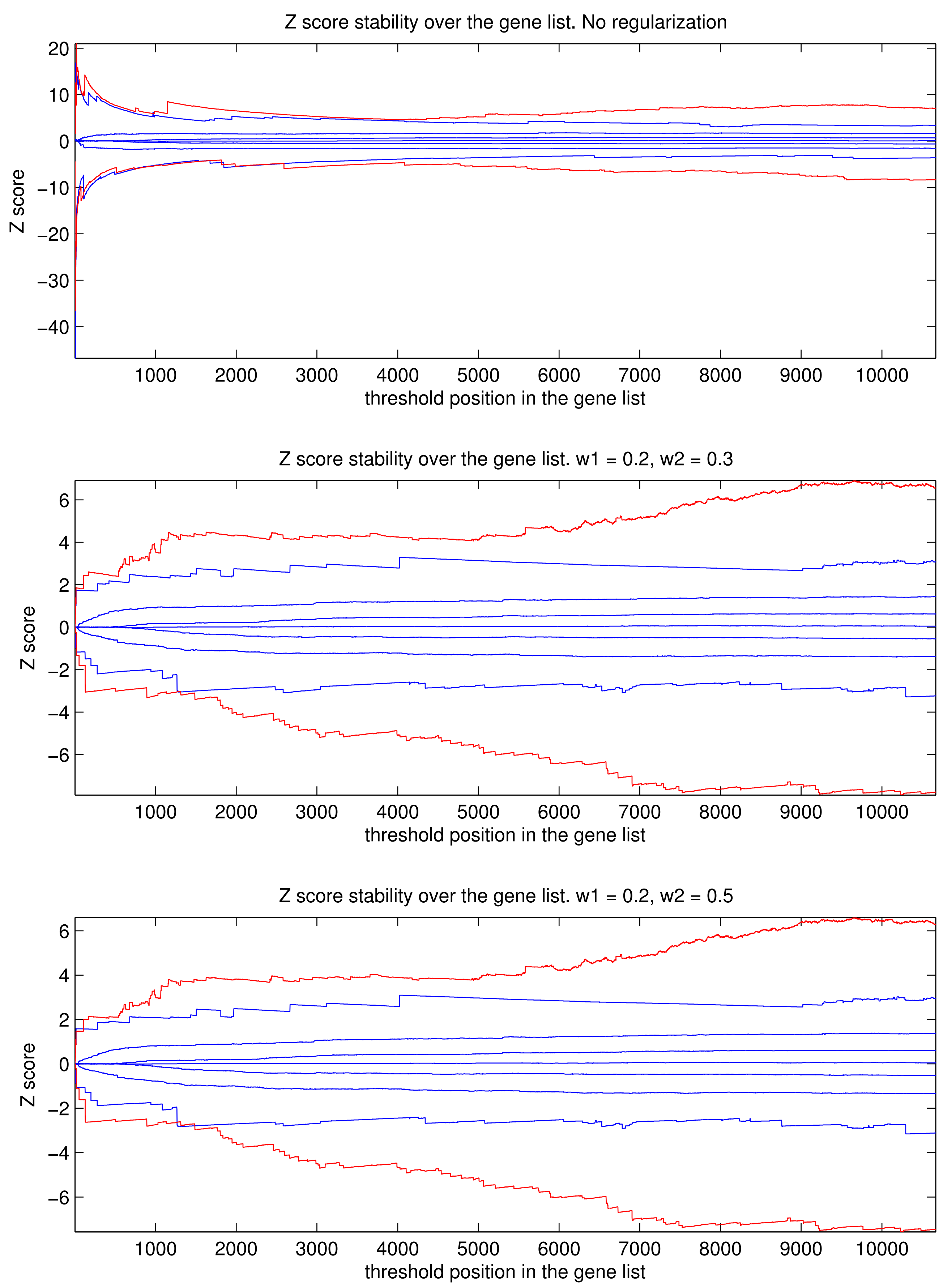
Z score stability over the gene list. No regularization
Z score
20
10
0
−10
−20
−30
−40
1000
2000
3000
4000
5000
6000
7000
8000
9000
10000
threshold position in the gene list
Z score stability over the gene list. w1 = 0.2, w2 = 0.3
Z score
6
4
2
0
−2
−4
−6
1000
2000
3000
4000
5000
6000
7000
8000
9000
10000
threshold position in the gene list
Z score stability over the gene list. w1 = 0.2, w2 = 0.5
Z score
6
4
2
0
−2
−4
−6
1000
2000
3000
4000
5000
6000
7000
8000
9000
10000
threshold position in the gene list
