# Supplementary figures and images for: Robust extraction of functional signals from gene set analysis using a generalized threshold free scoring function
Source: BMC Bioinformatics. 2009 Sep 23;10:307. doi: 10.1186/1471-2105-10-307 (PMC2761411; doi:10.1186/1471-2105-10-307)

KS score stability over the gene list

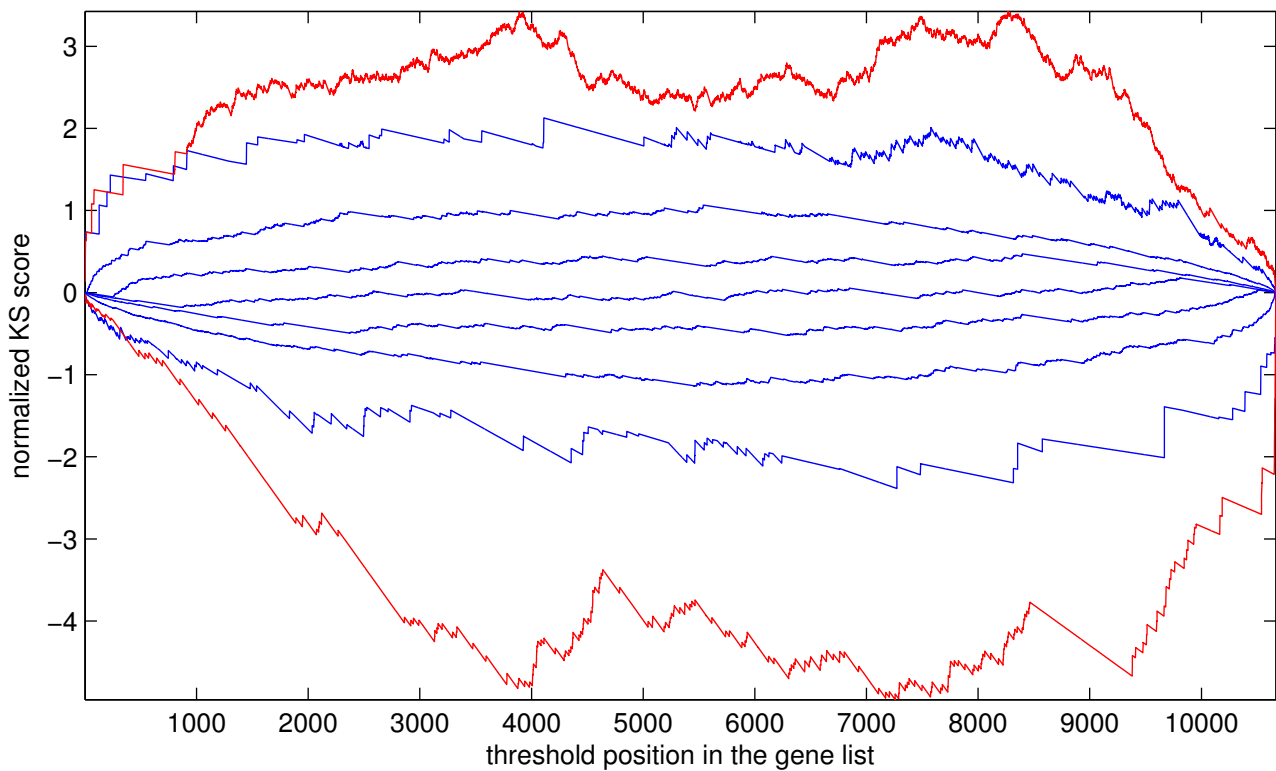

modKS score stability over the gene list

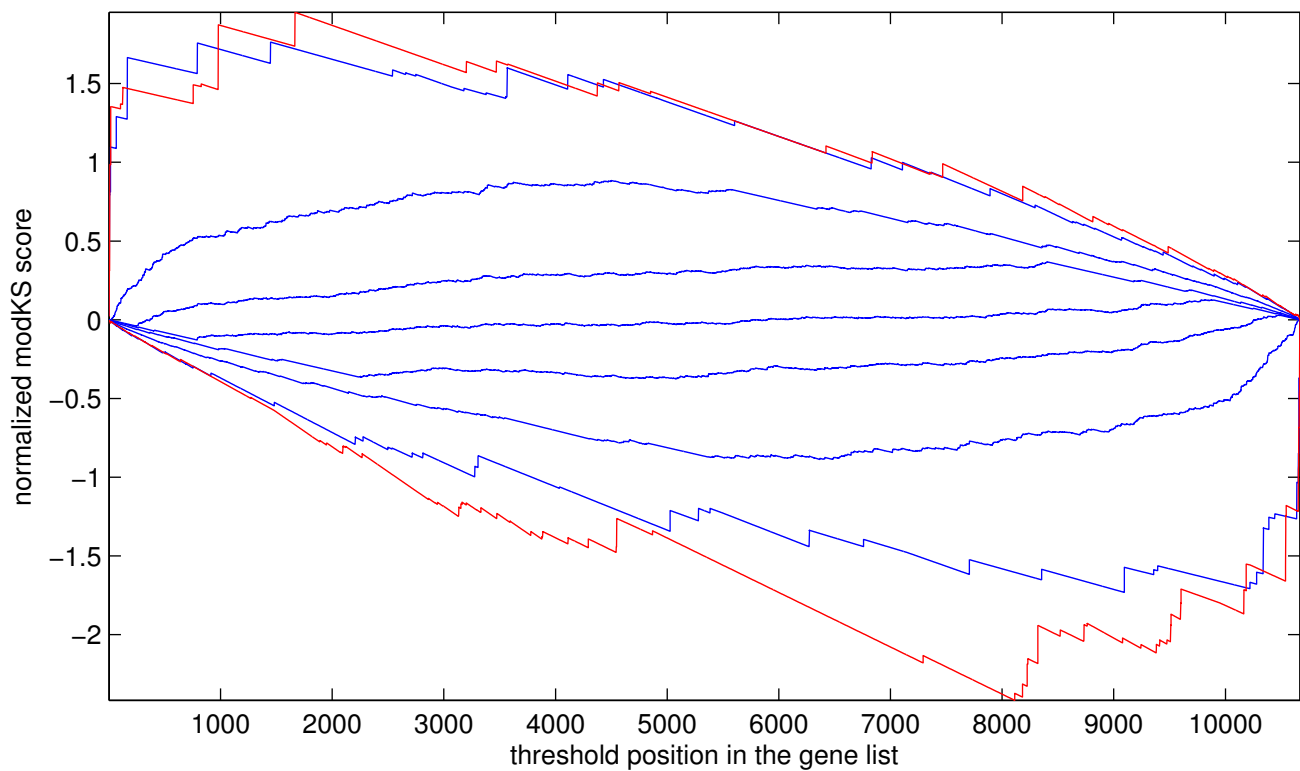

Supplement: Additional file 4 — Supplementary figure S2: Stability of KS and modKS as the threshold goes through the gene list. Behaviour of the KS test and the modKS test with the same randomized and positive dataset. Lines represent the same percentiles as in the supplementary figure S1 [see additional file 3] with the same colouring. Notice the biases between different threshold positions, especially when the results are compared with the earlier supplementary figure S1 [see additional file 3]. Notice also the less clear separation between the negative and the positive dataset for modKS in the lower figure. [file 1471-2105-10-307-S4.PDF]

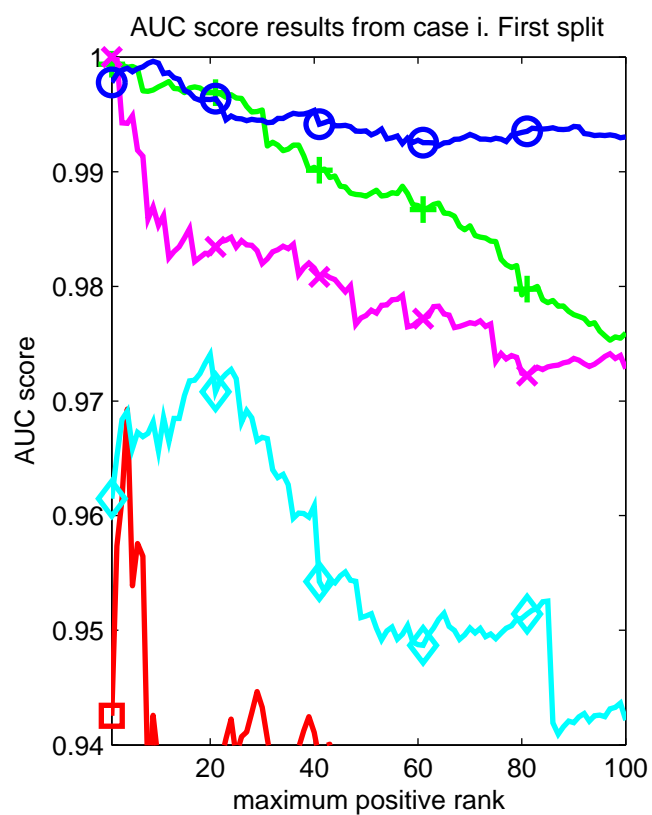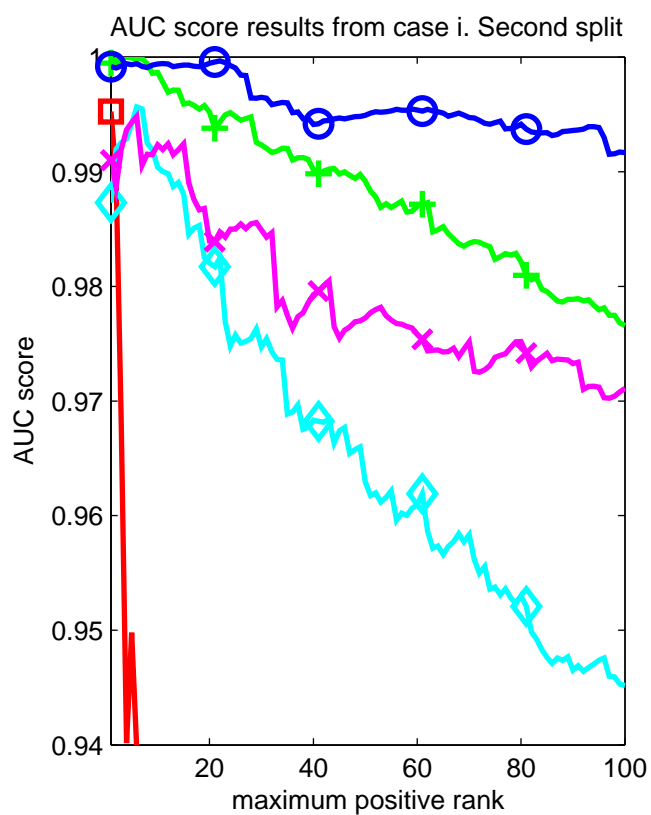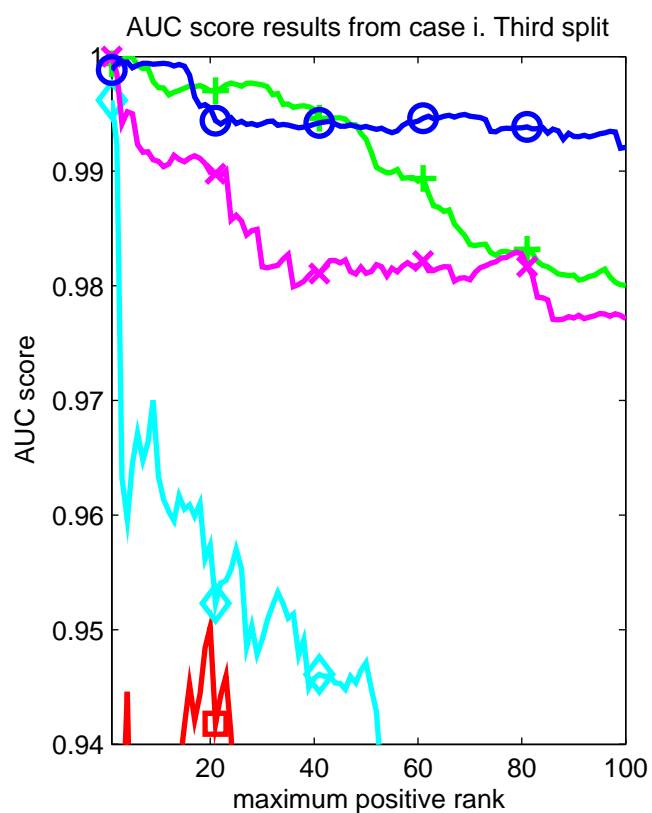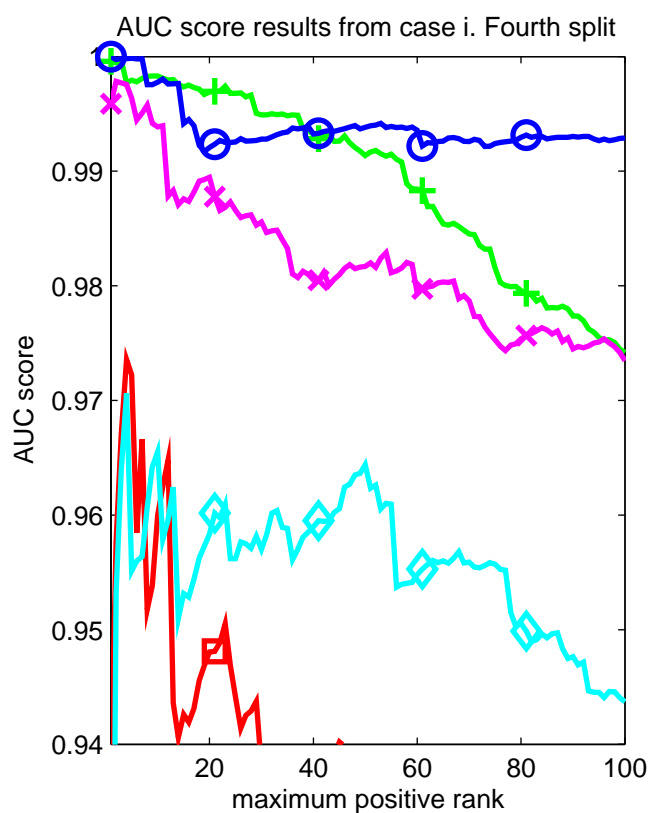

Supplement: Additional file 5 — Supplementary figure S3: Performance comparison in case (i) with each split analyzed separately. Performance of the methods in each split in case i. Figure represents the AUC score for each evaluated method as the rank limit of the positive GO classes is increased. Note that AUC is calculated here using the whole evaluated GO class list, and it is the size of the used positive GO class set that varies. Methods represented are GSZ-score: blue line with circles, t-test: green line with cross, KS test: red line with box, modKS test: cyan line with diamond, iGA: magenta line with x. Notice that although the signal levels vary between the replicates, the differences between the methods are stable. GSZ-score and t-test show equal performance among the smallest ranks, whereas the GSZ-score is clearly the best among the larger ranks. Other methods show weaker signal. Figure is zoomed to the upper signal area so that most of the curve for the KS is left outside. [file 1471-2105-10-307-S5.PDF]
