# Supplementary material for: Robust extraction of functional signals from gene set analysis using a generalized threshold free scoring function
Source: BMC Bioinformatics. 2009 Sep 23;10:307. doi: 10.1186/1471-2105-10-307 (PMC2761411; doi:10.1186/1471-2105-10-307)
Supplement: Additional file 6 — Supplementary figure S4: Performance comparison in case (ii) with each split analyzed separately. Performance of methods in each split in the case ii. The figure represents the AUC score for each evaluated method as the rank limit of the positive GO classes is increased. Methods are coloured identically to the earlier figure. Here, GSZ-score and iGA show the best performance at very top ranks, with GSZ-score slightly surpassing iGA. Note that performance of t-test and modKS varies considerably across the replicates. After rank 20 GSZ-score is constantly best in all the replicates. [file 1471-2105-10-307-S6.PDF]

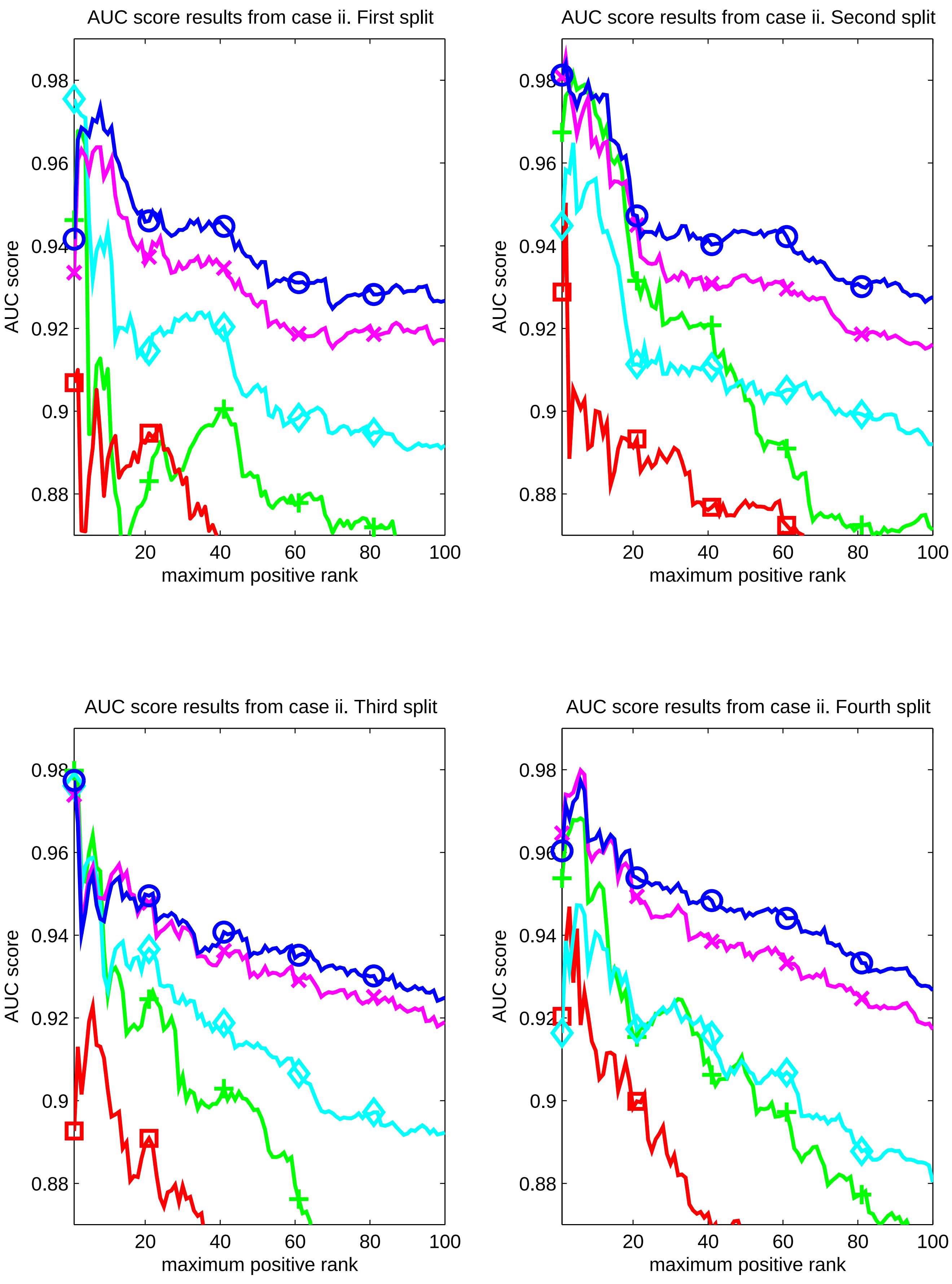
AUC score results from case ii. First split
AUC score results from case ii. Second split
AUC score results from case ii. Third split
AUC score results from case ii. Fourth split
AUC score
maximum positive rank
0.98
0.96
0.94
0.92
0.9
0.88
20
40
60
80
100
